# Endothelial ADGRF5(GPR116) governs vascular adaptation required for sustained thermogenic remodeling of brown adipose tissue

**DOI:** 10.1016/j.molmet.2026.102346

**Published:** 2026-03-06

**Authors:** Rabih El-Merahbi, Vasiliki Karagiannakou, Ronja Kardinal, Lea Seep, Richard Lindner, Michelle Ynonne Jäckstein, Staffan Hildebrand, Mersiha Hasic, Eylül Korkmaz, Ankush Kumar Jha, Aspasia Thodou Krokidi, Kenneth Dyar, Felix Meissner, Stephan Grein, Jörg Heeren, Martin Klingenspor, Alexander Pfeifer, Jan Hasenauer, Dagmar Wachten, Stephan Herzig, Anastasia Georgiadi

**Affiliations:** 1 Institute for Diabetes and Cancer (IDC), Helmholtz Diabetes Center Munich, Neuherberg, Germany; 2 Helmholtz Center Munich, German Research Center for Environmental Health, Computational Health Center, Munich, Germany; 3 German Center for Diabetes Research (DZD), Neuherberg, Germany; 4 Biophysical Imaging, Institute of Innate Immunity, Medical Faculty, University of Bonn, Bonn, Germany; 5 Life and Medical Sciences (LIMES) Institute and Bonn Center for Mathematical Life Sciences, University of Bonn, Bonn, Germany; 6 Helmholtz Center Munich, Core Facility Pathology and Tissue Analytics, Germany; 7 Department of Biochemistry and Molecular Cell Biology, University Medical Center Hamburg-Eppendorf, Hamburg, Germany; 8 Institute of Pharmacology and Toxicology, University Hospital, University of Bonn, Bonn, Germany; 9 Chair of Molecular Nutritional Medicine, TUM School of Life Sciences, Technical University of Munich, Freising, Germany; 10 Systems Immunology and Proteomics, Institute of Innate Immunity, Medical Faculty, University of Bonn, Bonn, Germany; 11 Joint Heidelberg-IDC Translational Diabetes Program, Inner Medicine 1, Heidelberg University Hospital, Heidelberg, Germany; 12 Molecular Metabolic Control, Medical Faculty, Technical University of Munich, Germany

**Keywords:** Single-nucleus RNA sequencing, Adhesion GPCRs, ADGRF5(GPR116), Brown adipose tissue, Endothelial paracrine signaling

## Abstract

**Objectives:**

Brown adipose tissue (BAT) dissipates energy via non-shivering thermogenesis, but durable thermogenic benefit requires sustained cold remodeling that stabilizes a cold-adapted tissue state. While most studies have focused on adipocyte-intrinsic pathways that drive acute activation, how stromal niche cells—particularly the vasculature—sense and coordinate long-term adaptation remains poorly defined. Because GPCRs are key sensors of extracellular and neurohumoral cues, we mapped GPCR expression across mouse and human BAT at single-nucleus resolution and identified adhesion GPCRs as a prominent family enriched in vascular cells, with endothelial ADGRF5(GPR116) emerging as a leading candidate regulator.

**Methods:**

Single-nucleus RNA sequencing of mouse and human BAT was used to map GPCR expression across cell types. Global, inducible endothelial-specific, and adipocyte-specific ADGRF5(GPR116) knockout mouse models were each challenged with acute and prolonged cold exposure. Endothelial and adipocyte states were analyzed using single-nucleus RNA sequencing transcriptional profiling, functional vascular assays, and cell–cell communication modeling.

**Results:**

Endothelial deletion of ADGRF5(GPR116) impaired the ability of mice to sustain thermogenesis during prolonged cold exposure, whereas adipocyte-specific deletion did not affect thermogenic capacity in vivo. Loss of endothelial ADGRF5(GPR116) did not alter endothelial cell abundance, but induced endothelial transcriptional reprogramming characterized by disrupted quiescent remodeling programs, shifts in endothelial state with EndMT-like features, and context-dependent alterations in barrier-associated pathways, occurring in the absence of immune cell infiltration or overt fibrosis. Adipocyte reclustering revealed a failure to acquire a fully cold-adapted thermogenic state, with thermogenically inefficient programs and adrenergic hyporesponsiveness, despite preserved sympathetic input. CellChat and NicheNet analyses predicted altered endothelial-derived paracrine signaling capable of reshaping adipocyte identity.

**Conclusions:**

Endothelial ADGRF5(GPR116) is a critical regulator of vascular adaptation during sustained cold exposure and supports full acquisition of the thermogenic adipocyte state through endothelial identity and paracrine signaling.

## Highlights

• Adhesion GPCRs constitute the second most abundant GPCR family in mouse and human brown adipose tissue.
• Adhesion GPCRs are enriched in non-adipocyte cell types, with highest expression in vascular endothelial cells.
• Endothelial ADGRF5(GPR116) is essential for maintaining thermogenic adaptation during prolonged cold exposure.
• ADGRF5(GPR116) is essential for the acquisition of cold-remodeled adipocyte states.
• Loss of ADGRF5(GPR116) disrupts endothelial quiescence during cold-induced vascular remodeling.

## Graphical abstract

During prolonged cold exposure, endothelial ADGRF5(GPR116) is required to maintain a homeostatic angiocrine environment that stabilizes thermogenic adipocyte identity and sustains energy expenditure. In wild-type brown adipose tissue, ADGRF5(GPR116)-expressing endothelial cells support adaptive intercellular communication and long-term thermogenic competence. Loss of endothelial ADGRF5(GPR116) during cold adaptation leads to altered endothelial signaling and maladaptive intercellular communication within the adipose microenvironment, resulting in impaired maintenance of thermogenesis despite preserved tissue structure.

**Figure:**
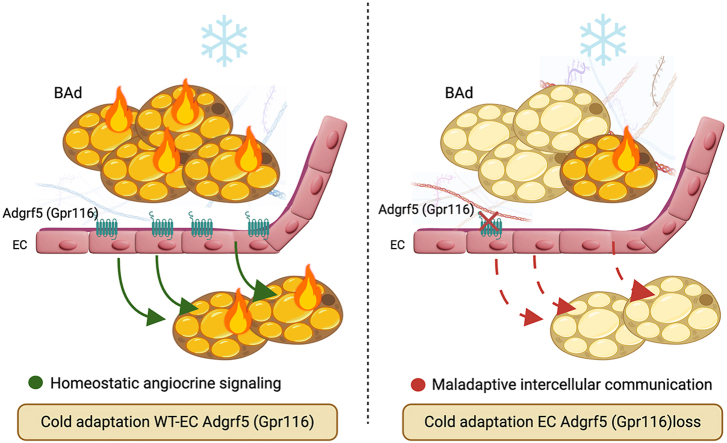

## 1 Introduction

Brown adipose tissue (BAT) is a specialized thermogenic organ that dissipates energy as heat via non-shivering thermogenesis (NST). It has garnered increasing attention not only for its potential to reduce adiposity but also for its endocrine functions that improve systemic metabolism [1,2]. Functional BAT is present in adult humans and is activated by cold exposure or β-adrenergic stimulation [[3], [4], [5]]. Importantly, BAT mass inversely correlates with the incidence of prediabetes, hyperlipidemia, and hypertension, suggesting a protective role in cardiometabolic health [6]. Additionally, white adipose tissue (WAT) can acquire brown-like characteristics in response to environmental or hormonal cues, giving rise to beige adipocytes with thermogenic capacity [7]. These findings underscore the therapeutic potential of enhancing BAT or beige fat activity to address obesity and related metabolic diseases.

Prolonged residence in thermoneutral environments and aging are associated with a progressive decline in brown adipose tissue (BAT) volume and activity in humans, limiting the persistence of thermogenic adipocytes over time [6,[8], [9], [10]]. As a result, meaningful therapeutic strategies aimed at harnessing BAT for cardiometabolic benefit must extend beyond transient activation and instead address the mechanisms that establish, remodel, and maintain a functional thermogenic tissue. While acute activation of brown adipocytes is sufficient to initiate thermogenesis, sustained thermogenic competence requires broader tissue remodeling that stabilizes a cold-adapted state [11]. From a physiological and translational perspective, this distinction is critical, as long-term metabolic benefit is unlikely to arise from transient activation alone but instead from the establishment of a remodeled BAT program capable of maintaining thermogenic capacity beyond acute stimulation [12].

Accumulating evidence indicates that such remodeling depends on coordinated adaptations within the stromal niche, particularly the vasculature, which supports thermogenesis by regulating perfusion, vascular tone, neurovascular coupling, and the local paracrine environment [13]. Previous studies have shown that manipulation of endothelial- or niche-derived factors can profoundly enhance or impair BAT thermogenic recruitment, often in parallel with changes in vascular marker abundance and sympathetic innervation [[14], [15], [16]]. These findings underscore the importance of vascular contributions to BAT remodeling, but largely emphasize structural or early adaptive responses. By contrast, how endothelial cells adapt their identity and signaling programs to sustain thermogenic remodeling over time—and thereby maintain a permissive environment for efficient adipocyte thermogenesis—remains less well understood.

G protein–coupled receptors (GPCRs) constitute a central signaling framework through which cells sense extracellular cues and coordinate adaptive responses to neural, metabolic, and environmental stimuli. In thermogenic adipose tissue, GPCR research has predominantly focused on adipocyte-intrinsic pathways, including but not limited to β-adrenergic signaling, with additional GPCR systems shown to modulate thermogenic activity [17,18]. These pathways have mostly provided important insight into the acute activation of thermogenesis, but do not fully explain how thermogenic competence is established and maintained during long-term tissue remodeling. In the vasculature, individual GPCR pathways—such as vasoactive endothelin receptors and barrier-associated sphingosine-1-phosphate receptors—have been implicated in processes relevant to thermogenic adipose remodeling [19,20]. However, how GPCR signaling within endothelial and vascular compartments contributes to sustained thermogenic adaptation remains incompletely defined. This gap motivated an unbiased, cell-type–resolved analysis to identify GPCRs enriched in non-adipocyte compartments of brown adipose tissue, that may regulate long-term thermogenic remodeling.

In this study, we performed an unbiased single-nucleus transcriptomic screen to map GPCR expression across cell types in mouse and human brown adipose tissue. This analysis identified adhesion GPCRs (aGPCRs) as a prominent GPCR family within vascular compartments and revealed *Adgrf5(Gpr116)* as a highly enriched endothelial receptor. Using a combination of global, endothelial-specific, and adipocyte-specific genetic deletion mouse models together with acute and prolonged cold exposure, we investigated the role of endothelial ADGRF5(GPR116) in thermogenic adipose tissue adaptation. Our findings uncover an essential contribution of endothelial ADGRF5(GPR116) to sustained thermogenic remodeling and the acquisition of fully thermogenically competent adipocytes.

## 2 Materials and methods

### 2.1 Animal models and in vivo experimental procedures

All mice (Mus musculus) were born and maintained in a temperature-controlled (22 °C) room on a 12-h light/dark cycle. Mice were fed a regular unrestricted chow diet (Kliba Nafag #3437, Provimi Kliba AG, Kaiseraugust). Chow-fed mice were housed 4–5 mice per cage. Mice were single-housed for the duration of the indirect calorimetry experiments. Body weight was measured with a table scale, and body composition (lean and fat mass) was measured via nuclear magnetic resonance technology (EchoMRI). All animal handling procedures were performed under the European Union directives and the German Animal Welfare Act and have been approved by the institutional animal welfare officer and local authorities under animal licenses (ROB-55.2-2532.Vet_02-21-133).

#### 2.1.1 Transgenic mouse models

*Global ADGRF5(GPR116) knockout KO mice*: Global *ADGRF5(GPR116)* knockout (KO) mice have been previously described by Yang et al. [21] and Fukuzawa T et al. [22] which carry a deletion of exon 2, which encodes for the start codon and signal peptide (SP) of the *Adgrf5(Gpr116)* gene. The mice were a gift from Brad Croix. They were backcrossed ten generations, upon arrival, and they were on a clean C57Bl6N genetic background. Genotyping was done with primers: WT: GGAGGCTCTGTGCGTTTC, R1: CTGTGGACATGATGAAGGGTG, R2: CTCCCTGAATCATAGTCTAGTCTCC. PCR reaction was performed with DreamTaq (ThermoScientific) using the following PCR protocol: Denaturation 95 °C (5min), (denaturation 95 °C 30sec, annealing: 60 °C 30sec, elongation: 72 °C 1min) for 35 x cycles and final extension 72 °C for 10min.

*Ucp1Cre x ADGRF5(GPR116)f/f* *mice*: Conditional ADGRF5(GPR116) floxed mice, (B6N.Cg-Adgrf5^tm1.1Bstc/J^, Jackson Laboratory #:022505, in which loxP sites flank exon 2 of the *Adgrf5(Gpr116)* gene, were crossed with Ucp1Cre (B6.FVB-Tg(Ucp1-cre)1Evdr/J, Jackson Laboratory strain #024670), to generate Ucp1CrexADGRF5(GPR116)f/f mice. PCR genotyping was done with primers: For Ucp1Cre forward 1: CAAGGGGCTATATAGATCTCCC, Cre reverse 1: ATCAGAGGTGGCATCCACAGGG, Cre reverse 2: GTTCTTCAGCCAATCCAAGGG). *Adgrf5(Gpr116)flox* genotyping was performed using the same primers as in the global ADGRF5(GPCR116) KO. PCR was run with DreamTaq (ThermoScientific) with the following protocol: Denaturation 95 °C (5min), (denaturation 95 °C 30sec, annealing: 60 °C 30sec, elongation: 72 °C 1min) for 35 x cycles and final extension 72 °C for 10min. PCR products were assessed on 2% agarose gels (UCP1Cre band: 336bp, WT band: 554bp; ADGRF5(GPR116)flox/flox band 340bp and WT band 292bp).

*AdiponectinCre × ADGRF5(GPR116)f/f mice:* Conditional ADGRF5(GPR116) floxed mice (B6N.Cg-Adgrf5ˆtm1.1Bstcˆ/J; Jackson Laboratory strain #022505), in which loxP sites flank exon 2 of the *Adgrf5(Gpr116)* gene, were crossed with B6; FVB-Tg(Adipoq-cre)1Evdr/J mice (Jackson Laboratory strain #010803) to generate AdiponectinCre × ADGRF5(GPR116)f/f mice. Genotyping for the Adiponectin-Cre transgene was performed by PCR using the following primers: forward 5′-GAACCTGATGGACATGTTCAGG-3′ and reverse 5′-AGTGCGTTCGAACGCTAGAGCCTGT-3′. Primers for Myogenin were included as an internal control (forward 5′-TTACGTCCATCGTGGACAGC-3′ and reverse 5′-TGGGCTGGGTGTTAGCCTTA-3′).

PCR amplification was performed using DreamTaq DNA polymerase with the following cycling conditions: initial denaturation at 95 °C for 5 min, followed by 35 cycles of denaturation at 95 °C for 30 s, annealing at 60 °C for 30 s, and extension at 72 °C for 1 min, with a final extension at 72 °C for 10 min.

*Cdh5CreERT2 x ADGRF5(GPR116)f/f* *mice*: Cdh5-CreERT2 mice (C57BL/6-Tg(Cdh5-cre/ERT2)1Rha) were generated by Ralf H Adams [23], and obtained from the Mary Lyon Centre (EMMA ID: EM14891). Genotyping was performed using primers forward TCCTGATGGTGCCTATCCTC and reverse CCTGTTTTGCACGTTCACCG. PCR was performed using DreamTaq DNA polymerase with an initial denaturation at 95 °C for 1 min, followed by 29 cycles of 95 °C for 10 s, 60 °C for 10 s, and 72 °C for 1 min, and a final extension at 72 °C for 30 s. The Cdh5-CreERT2 band was detected at 594 bp.

#### 2.1.2 Inducible endothelial recombination

The Cdh5Cre expression was induced by intraperitoneal (i.p.) injections of tamoxifen (Sigma, T5648) at 100 mg/kg/day for 5 consecutive days. Tamoxifen was prepared as a 20 mg/mL working solution by dissolving 60 mg in 0.5 mL pure ethanol, followed by the addition of 2.5 mL corn oil. Mice were allowed a 2-week washout period prior to cold exposure. To maintain efficient recombination during prolonged cold exposure, tamoxifen was administered again (100 mg/kg/day, i.p.) for 3 consecutive days starting on day 7 of cold exposure. Recombination efficiency was assessed by qPCR in CD31^+^ BAT endothelial cells using *Adgrf5(Gpr116)-*specific primers (FW: TTCTTCAGAAGCTGCGCTGA; RV: CCCGGCTAGCTCATGTTCTT).

#### 2.1.3 Dextran perfusion (vascular permeability test)

Vascular permeability was assessed by intravenous injection of fluorescein isothiocyanate–conjugated dextran (70 kDa; FITC–dextran, Sigma–Aldrich, cat. no. 46945-100 MG-F). For intravenous administration, mice were briefly removed from cold housing and tail-warmed for approximately 3–4 min by immersion in warm water to facilitate venous access. FITC–dextran was then injected intravenously via the tail vein. Immediately after injection, mice were returned to 8 °C and maintained under cold conditions for 13–15 min to allow tracer circulation. Animals were subsequently transferred to room temperature and anesthetized with ketamine/xylazine. After loss of reflexes (approximately 5 min), mice were transcardially perfused with 30 mL phosphate-buffered saline (PBS) supplemented with heparin (final concentration ∼10 IU/mL; heparin sodium, 100 IU/mL stock, Sanofi/Aventis) at a flow rate of 8 mL/min to remove intravascular tracer. The total circulation time of FITC–dextran prior to perfusion was approximately 20 min.

Brown adipose tissue (BAT) and inguinal white adipose tissue (iWAT) and other organs were harvested immediately after perfusion and flash-frozen in liquid nitrogen. Tissues were homogenized in PBS containing 0.05% Triton X-100, 5 mM EDTA, and 20 mM HEPES (10% w/v) using two stainless-steel beads per sample in a TissueLyser (1.5 min at 30 Hz). Homogenates were transferred to fresh tubes without beads and centrifuged for 20 min at 10,000 rpm at 4 °C. FITC–dextran extravasation was quantified from the clarified supernatant by fluorescence measurement, using a plate reader (VarioscanLux) and normalized to total protein content as determined by a bicinchoninic acid (BCA) assay.

### 2.2 Isolation of BAT endothelial cells for qPCR analysis

Endothelial cells were isolated from freshly harvested mouse BAT using magnetic-activated cell sorting (MACS). BAT depots were dissected, minced, and enzymatically dissociated using the Adipose Tissue Dissociation Kit (Miltenyi Biotec, #130-105-808) in gentleMACS™ C tubes. Homogenization was performed using the gentleMACS™ Dissociator, program 37C_mr_ATDK_1. Cell suspensions were filtered through 100 μm and 40 μm strainers, subjected to red blood cell lysis (ACK buffer) for 1min, and incubated with Fc receptor blocking reagent (Miltenyi). CD31^+^ cells were labeled with CD31 microbeads (Miltenyi, #130-097-418) and isolated using LS columns. CD31^+^ and flow-through fractions were centrifuged at 300×*g* for 20 min at 4 °C and stored in TRIzol at −80 °C for RNA extraction.

### 2.3 Cold challenge, adrenergic stimulation, and indirect calorimetry

Energy expenditure, locomotor activity, respiratory exchange ratio, and food intake were measured using indirect calorimetry over indicated periods (PhenoMaster; TSE Systems). Mice were acclimated to the system for 7 days prior to measurements.

For acute cold exposure, ambient temperature was reduced from 22 °C to 4 °C over 30 min and maintained for up to 8 h. For prolonged cold exposure, temperature was gradually reduced from 22 °C to 8 °C over 7 days (2 °C every 12 h), followed by maintenance at 8 °C for the indicated duration. Indirect calorimetry data were analyzed using the CalR platform.

For pharmacological activation of β3-adrenergic signaling, mice were injected intraperitoneally with CL-316,243 (2 mg/kg; Sigma, C5976). All challenges were performed in adult mice 9–12 weeks old.

### 2.4 Body temperature measurements

Core body temperature was monitored using telemetric probes (TA-F10, Data Sciences International) implanted intraperitoneally under isoflurane anesthesia. Mice received perioperative analgesia with buprenorphine and carprofen. After at least one week of recovery, temperature data were recorded continuously and analyzed in 60-min intervals. Surface temperature and tail heat loss were additionally assessed by infrared thermal imaging.

### 2.5 Histology and microscopy

#### 2.5.1 Formalin-fixed paraffin-embedded tissue processing

Excised tissue specimens were fixed in 4% (w/v) neutral-buffered formalin, embedded in paraffin, and sectioned at a thickness of 3 μm. Sections were used for hematoxylin and eosin (H&E) staining, Sirius Red/Fast Green collagen staining, immunohistochemistry (IHC), and immunofluorescence (IF) analyses. For UCP1 immunohistochemistry, 3 μm sections of BAT and iWAT were stained on the DISCOVERY ULTRA automated stainer (Roche Diagnostics), using a rabbit anti-UCP1 antibody (1:1000; Abcam, ab10983). Signal detection was performed using the ImmPRESS HRP Goat Anti-Rabbit IgG Polymer Detection Kit (Vector Laboratories, MP-7451-15), and visualization was achieved with ImmPACT DAB EqV Substrate (Vector Laboratories, SK-4103-100).

#### 2.5.2 Immunofluorescence staining of formalin-fixed, paraffin-embedded (FFPE) sections

Immunofluorescence co-stainings were performed on 3 μm paraffin sections following standard deparaffinization and antigen retrieval procedures. Endothelial cells were detected using a rat anti-CD31 antibody (1:20; Dianova, DIA-310, clone SZ31), followed by goat anti-rat IgG (H + L) DyLight™ 755–conjugated secondary antibody (1:100; Invitrogen, SA5-10023). For co-staining experiments, the following primary antibodies were used: Rabbit anti-Ki67 (1:1000; Abcam, ab15580) to assess cellular proliferation, Rabbit anti-CD68 (1:1000; Abcam, ab125212) to identify macrophages, secondary antibodies included goat anti-rabbit IgG Alexa Fluor™ 488 (1:400; Invitrogen, A-11034) and goat anti-rabbit IgG Alexa Fluor™ 750 (1:100; Invitrogen, A-21039), respectively. Nuclei were stained with Hoechst 33342 (Biomol, CDX-B0030). Stained sections were scanned using an AxioScan 7 digital slide scanner (Zeiss) equipped with a 20 × objective.

#### 2.5.3 Tissue clearing and immunofluorescence labeling of iWAT

For whole-mount imaging of iWAT vasculature, iWAT was processed using a methanol-based tissue clearing protocol. Briefly, tissues were dehydrated through a graded methanol series (20–100%), bleached in methanol containing 5% H_2_O_2_, rehydrated, and incubated in B1n buffer (0.3 M glycine, 0.1% Triton X-100). Antigen retrieval was performed using a urea-based buffer overnight at 4 °C. Tissues were digested with 0.2% collagenase A for 30 min at 37 °C, blocked in SUMIC blocking buffer, and incubated with rat anti-CD31 antibody (1:400; BD Pharmingen, #553370) for 2–3 days at 4 °C. Following extensive washing, tissues were incubated with donkey anti-rat Alexa Fluor™ 488 secondary antibody (1:500; Dianova, #712-545-153) for 2–3 days. Samples were dehydrated through an isopropanol series and cleared in ethyl cinnamate/PEGM. Imaging was performed using a Leica Stellaris 8 confocal microscope with 10 × , 20 × (water immersion), and 63 × (water immersion) objectives, and images were processed using LAS X v4.5.0 software.

#### 2.5.4 Vascular parameters

Confocal images were converted to maximum-intensity projections and analyzed using AngioTool v0.5 to identify vessel elements, junctions, endpoints, and vessel borders. Tissue area and vessel-covered area were quantified using ImageJ, and vascular maturation parameters were calculated accordingly.

#### 2.5.5 Quantitative image analysis

Automated digital image analysis was performed using Visiopharm software (Hørsholm, Denmark). Regions of interest (ROIs) were defined as tissue-containing areas excluding background and artefacts; all quantitative measurements (including % UCP1-positive area, % CD31-positive area and mean adipocyte cross-sectional area) were normalized to the total ROI area.

### 2.6 Immunoblotting

Frozen tissue was homogenized in lysis buffer containing 10 mM Tris–HCl (pH 8.0), 1 mM EDTA, 0.5 mM EGTA, 1% Triton X-100, 0.1% sodium deoxycholate, 0.1% SDS, and 140 mM NaCl, supplemented with protease and phosphatase inhibitors (Thermo Fisher Scientific). Adipose tissue lysates were further centrifuged to remove the lipid layer. Protein concentrations were determined using a bicinchoninic acid (BCA) assay (Thermo Fisher Scientific).

Proteins were resolved on precast Tris–glycine SDS–PAGE gels (Novex™, Thermo Fisher Scientific) and transferred to PVDF membranes (Bio-Rad). Membranes were probed with antibodies against tyrosine hydroxylase (Sigma–Aldrich, MAB318) and VCP (Abcam, ab11433) as a loading control.

### 2.7 ELISA kits

For measurement of serum thyroid hormone levels (T3) we used the mouse Triiodothyronine (T3) ELISA Kit from BIOZOL, cat. Number MBS262762-96.

### 2.8 Serum analyser

Measurements of serum lipids and lactate were performed using the serum analyzer (AU480 Beckman Coulter).

### 2.9 Measurement of norepinephrine in mouse plasma and tissue

Plasma norepinephrine was quantified using high-performance liquid chromatography (HPLC) coupled with electrochemical detection (EcD), as previously described [24]. Sample preparation followed the protocol provided by RECIPE Chemicals + Instruments GmbH for catecholamine extraction in human plasma with the ClinRep® complete kit, which includes all necessary reagents and materials for the extraction of the desired analytes. Due to the limited volume of mouse plasma/serum, modifications to the standard protocol were required. Specifically, 50 μL of mouse plasma/serum was mixed with 100 μL of 0.3 M perchloric acid (in water) and 5 μL of internal standard (DHBA, 1 ng/μL). After thorough mixing, the sample was centrifuged at 9,000 rpm for 5 min. The resulting supernatant was charged to the sample preparation column, which was then shaken for 10 min. Solvent was removed using a vacuum manifold, and the column was washed three times with 1 mL of wash solution to eliminate interfering substances. After drying the column, 150 μL of elution reagent was added. The column was shaken for 5 min, and then the catecholamines were eluted from the extraction column via centrifugation. A 20 μL aliquot of the final eluate was injected into the HPLC-EcD system for analysis.

For tissue norepinephrine measurements, snap-frozen BAT and iWAT samples (100 mg weight) were processed using the same extraction and analytical procedure as described above. Frozen tissue samples were homogenized in 0.3 M perchloric acid containing internal standard (DHBA), centrifuged, and the resulting supernatants were subjected to catecholamine extraction and HPLC-EcD analysis as for plasma samples.

### 2.10 Hydroxyproline assay

Collagen content was quantified using a hydroxyproline assay (QuickZyme, QZBTISCOL1). Tissues were hydrolyzed in 6 M HCl (10% w/v) at 95 °C for 20 h. Samples were centrifuged at 13,000×*g* for 10 min, diluted, and processed according to the manufacturer's instructions. Absorbance was measured at 570 nm.

### 2.11 Nuclei isolation and fixation

BAT or iWAT from two mice per ambient temperature condition was pooled for each sample. All steps were performed on ice using pre-cooled reagents, tubes, and a swing-bucket centrifuge to preserve nuclear integrity. Snap-frozen adipose tissues were first fragmented into small pieces using a mortar and pestle pre-cooled with liquid nitrogen. The frozen tissue fragments were immediately transferred into 50 mL gentleMACS™ C Tubes (Miltenyi Biotec, 130-128-024) prefilled with nuclei extraction buffer (Miltenyi Biotec) at a ratio of 2 mL per 200 mg of tissue, supplemented with 10,000 units of RNasin® Plus RNase Inhibitor (Promega, N2615). Tissue homogenization was carried out using the gentleMACS™ Dissociator device, running a 5-minute program optimized for nuclei extraction (4C_nuclei_1). The program was repeated twice to ensure complete dissociation of the tissue. The resulting homogenate (typically 2–4 mL per sample) was brought up to a final volume of 10 mL using resuspension buffer consisting of 1% BSA, 2 mM MgCl_2_, and 0.2 U/μL RNase inhibitor. The homogenate was then filtered through a 70 μm cell strainer (Miltenyi Biotec, 130-098-462) into a fresh 15 mL conical tube and centrifuged at 500×*g* for 5 min at 4 °C. The supernatant was removed using a vacuum pump, and the pellet was resuspended in 1 mL of resuspension buffer, filled again to 10 mL, and centrifuged under the same conditions to perform a second wash. The final pellet was resuspended in 500 μL of resuspension buffer, filtered through a 40 μm cell strainer, and transferred to a 1.5 mL low-binding microcentrifuge tube. Nuclei were counted manually using a hemocytometer (Sigma–Aldrich, BR717805-1 EA) and subsequently fixed according to the Parse Biosciences Nuclei Fixation Protocol v2. Following fixation, each nuclei suspension was counted again and divided into two aliquots: one for pre-barcoding quantification and one reserved for the barcoding step. Fixed nuclei were stored at - 80 °C until further processing.

### 2.12 Single-nucleus library preparation and sequencing

Fixed nuclei suspensions from each experimental condition were processed for single-nucleus transcriptomic profiling using the Split-Seq combinatorial barcoding method, as implemented in the Parse Biosciences Evercode™ Whole Transcriptome v2 kit. For each condition, we targeted the recovery of approximately 25,000 to 30,000 barcoded nuclei. Following the barcoding steps, cDNA concentration from each library was measured using the Qubit High Sensitivity DNA Kit (Life Technologies, Q33230). The fragment size distribution of the cDNA was assessed with the Agilent Bioanalyzer using the High Sensitivity DNA Kit (Agilent Technologies, 5067-4626). Library preparation steps, including cDNA fragmentation and Illumina adaptor and index ligation, were performed according to the manufacturer's instructions provided with the Parse Biosciences kit. Final libraries were submitted to the Helmholtz Munich Genomics Core Facility for sequencing on an Illumina NovaSeq X Plus system.

### 2.13 Single-nucleus RNA sequencing data analysis

Raw FASTQ files generated for each sub-library were processed using the demultiplexing pipeline provided by Parse Biosciences (v2, Parse Biosciences, Inc., 2024). Reads were aligned to the mouse reference genome GRCm39. Subsequently, the eight sub-libraries were merged using the library-combination function implemented in the same Split-seq pipeline version. The resulting count matrices for each sample were used as input for downstream analysis in R (v4.x), primarily using the Seurat package v5 [25].

Count matrices from the different experimental conditions were merged using the merge() function in Seurat while retaining condition-specific metadata. Quality control filtering was applied to retain nuclei with fewer than 5,000 detected features, fewer than 20,000 total counts, and less than 15% mitochondrial gene content. These thresholds were chosen to minimize the contribution of low-quality nuclei, doublets, and ambient RNA contamination. Filtered gene expression values were normalized and scaled using the ScaleData() function.

Dimensionality reduction was performed using principal component analysis (PCA), followed by graph-based clustering using the FindNeighbors() and FindClusters() functions. Dataset integration across conditions was achieved using Harmony [26], and cluster identification was performed using the Leiden algorithm [27] with a resolution of 0.25. Uniform Manifold Approximation and Projection (UMAP) was applied to visualize the resulting joint embedding and to represent the major cell populations.

Cluster-specific marker genes were identified using the FindMarkers() function with the MAST statistical framework. To improve computational efficiency, differential expression analyses across clusters were parallelized using a custom bash script deployed on a high-performance computing (HPC) cluster. Cell type annotation was performed based on cluster-enriched marker genes, established literature, and reference single-cell and single-nucleus datasets.

Differential gene expression analyses comparing conditions and genotypes were also performed using FindMarkers() with MAST, applying an adjusted p-value cutoff of <0.05 and no predefined fold-change threshold.

To achieve higher-resolution characterization of thermogenic and vascular heterogeneity, adipocyte and vascular cell populations were subset from the main dataset and independently re-clustered using the same analytical pipeline. Subclustering was performed separately for BAT and iWAT. Adipocyte subclusters were identified using a clustering resolution of 0.5, while vascular subclusters were identified using a resolution of 1. Differentially expressed genes between adipocyte and vascular subclusters were determined using the MAST framework [28].

Finally, predefined gene signature scoring was performed using the AddModuleScore() function in Seurat. Module scores were calculated based on curated marker gene sets representing adipocyte thermogenic states, vascular endothelial subtypes, and functional programs, allowing quantitative comparison of transcriptional states across conditions and genotypes.

### 2.14 GPCRs expression analysis

GPCR classification was based on IUPHAR/BPS and HGNC databases. GPCR expression was quantified using scCustomize, considering receptors expressed in >1% of nuclei. For downstream analyses focusing on robust, cell type–enriched receptors, a more stringent cutoff of ≥30% expression within a given cell population was applied (related to Results 3.2). Comparative analyses were performed using a published human BAT snRNA-seq dataset.

### 2.15 Gene Ontology analysis

Gene Ontology (GO) enrichment analysis was performed using the *clusterProfiler* package [29]. Differentially expressed genes identified for each condition and genotype comparison were used as input for over-representation analysis and analyzed using the compareCluster() function with a p value cutoff of 0.05. To reduce redundancy among enriched GO terms, the simplify() function was applied to the resulting GO categories.

### 2.16 Cell–cell interaction and secreted factor analysis

Cell–cell communication analysis was performed using CellChat [30] and NicheNet [31] on the integrated single-nucleus dataset. A new identity column was added to the main Seurat object to incorporate the higher-resolution subclustering of adipocytes and vascular cells, allowing refined cell-type–specific interaction analyses across tissues. CellChat analysis was applied separately for each condition (WT_22 °C, WT_8 °C, KO_22 °C, KO_8 °C) to infer intercellular communication networks based on known ligand–receptor pairs. Individual CellChat objects were subsequently merged to identify condition- and genotype-dependent changes in signaling interactions as well as interactions shared across conditions.

To further resolve endothelial-to-adipocyte signaling relationships, NicheNet analysis was performed with endothelial subtypes defined as sender cells and adipocyte subtypes defined as receiver cells. Analyses were conducted for genotype-driven effects (KO cold vs WT cold) and cold-driven effects (WT cold vs WT RT). The top_n_ligands and top_n_targets parameters were set to 20 and 50, respectively.

In parallel, differentially expressed genes (adjusted p-value <0.05, no fold-change cutoff) derived from each vascular subtype for genotype- and condition-dependent comparisons were annotated using biomaRt [32] to identify genes encoding secreted factors and extracellular matrix–associated proteins. As an additional validation step, candidate genes were cross-checked against UniProt annotations to confirm subcellular localization, and only proteins annotated as secreted, extracellular, or extracellular matrix–associated were retained for downstream secretome analyses.

### 2.17 Regulons analysis

We performed transcription factor regulon analysis using the decoupler [33] library and the DoRothEA gene regulatory network [34], which contains information for transcription factors and their target genes from mouse and human. The endothelial cell types were subset from the Vascular subclustered dataset for each tissue and the analysis was performed in condition and cell type level. Subsequently, transcription factors were visualized in a heatmap with the ComplexHeatmap library in R.

### 2.18 Software and algorithms details, please see Supplementary Table 1

#### 2.18.1 Statistics and graphs generation

Energy expenditure was calculated using the Weir equation. Data analysis and statistics (which include ANOVA and ANCOVA) were performed using GraphPad Prism 10. The CalR platform was used to analyze the indirect calorimetry data.

### 2.20 Data availability

A web application app for data exploration is available at: https://shiny.iaas.uni-bonn.de/Shiny-GPCR/The raw files and analyzed objects can be shared upon request to the corresponding author.

### 2.21 Use of AI tools

OpenAI's ChatGPT (5.2 version) was used to assist with code troubleshooting and language editing. The results were checked and approved by the authors.

## 3 Results

### 3.1 Single-nuclei RNA sequencing screen identifies adhesion GPCRs as the second most abundant GPCR class in brown adipose tissue

**Figure 1:**
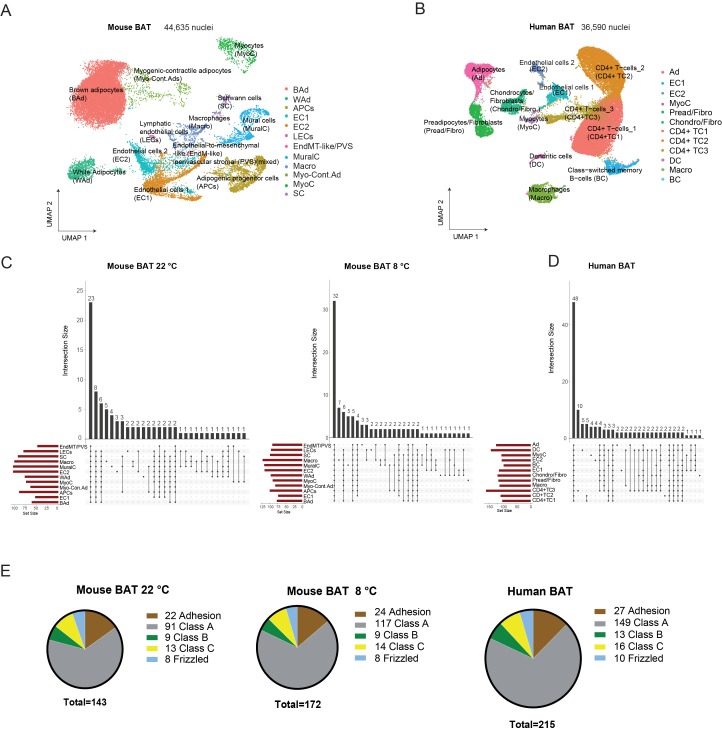
**Single-nucleus analysis of GPCRs in mouse and human BAT.** UMAP plots showing clustering of nuclei from (A) mouse BAT, (B) human BAT. (C–D) Upset plots showing the number of common and unique GPCRs across different cell types for mouse BAT (C) and human BAT (D). (E) Pie charts showing the percentage of represented classes of GPCRs in mouse and human single-nucleus datasets. Mice were single housed at 22 °C or 8 °C for 14 days. Single-nucleus RNAseq is a pool of BAT from two mice per ambient temperature condition. Mice were males, C57Bl6N, 10–12 weeks old.

Because sustained thermogenic remodeling requires coordinated adaptation across adipocyte and stromal compartments, we sought to define GPCR expression across all BAT cell types at single-nucleus resolution. Previous efforts to screen for GPCRs in BAT have been performed via pre-designed primers for qPCR or multiplexing arrays, on either whole mouse BAT tissue [35,36] or in vitro differentiated brown adipocytes [37]. However, a single-cell level unbiased screen is missing. To comprehensively characterize the distribution of GPCRs across different cell types in mouse and human BAT, we utilized single-nuclei RNA sequencing (snRNA-seq). To further investigate the effect of cold exposure on GPCR expression in different cell types, we utilized BAT tissue from mice housed at 22 °C (room temperature) and mice housed at 8 °C for 14 days. In total, we obtained 44,635 high-quality nuclei from mouse BAT (Figure 1A). For single-nucleus GPCR analysis in human BAT, we used a publicly available dataset [38] containing 36,590 nuclei (Fig. 1B).

Cell clustering of mouse BAT resolved 12 major cell populations, including brown adipocytes (BAd), white adipocytes (WAd), adipogenic progenitor cells (APCs), endothelial cells (ECs; two subclusters, EC1 and EC2), lymphatic endothelial cells (LECs), endothelial-to-mesenchymal–like/perivascular stromal population (EndMT-like/PVS), mural cells (MuralC; encompassing vascular smooth muscle cells and pericytes), macrophages (Macro), Schwann cells (SC), myocytes (MyoC), and a distinct population of myogenic-contractile adipocytes (Myo-Cont. Ad) (Fig. 1A). Cluster annotation was performed using canonical lineage markers, which showed clear, cluster-specific expression patterns consistent with published BAT single-cell datasets (Suppl. Fig. 1A; Extended File 1). Brown adipocytes (BAd) were defined by *Ucp1* and *Cidea*, white adipocytes (WAd) by *Lep* and *Cfd*, adipogenic progenitor cells (APCs) by *Pdgfra* and *Dpp4*, endothelial cells (ECs) by *Pecam1* and *Cdh5*, lymphatic endothelial cells (LECs) by *Prox1* and *Lyve1*, mural cells (MuralC) by *Rgs5* and *Acta2*, macrophages (Macro) by *Ptprc* and *Csf1r*, Schwann cells (SC) by *Sox10* and *Mpz*, and myocytes (MyoC) by muscle-associated contractile genes. The endothelial-to-mesenchymal-like/perivascular stromal (EndMT-like/PVS) population was characterized by enrichment of mesenchymal and transition-associated transcripts (e.g., *Vim*, *Zeb1*, *Snai1/2*, *Tgfbr1*), together with perivascular/stromal features, consistent with a mixed transition/perivascular stromal state (Suppl. Fig. 1A, Extended File 1). Myogenic-contractile adipocytes (Myo.-Cont. Ad) expressed mixed adipocyte and muscle-associated contractile gene programs and were therefore considered part of the adipocyte compartment in downstream analyses. One endothelial subcluster (EC2) displayed low-level enrichment of adipocyte-associated transcripts, a reproducible feature across independent BAT single-nucleus datasets [39], although whether this reflects rare transitional states or technical admixture cannot be conclusively resolved.

For the human dataset, we identified cell populations largely overlapping with those observed in mouse BAT, including adipocytes (Ad), myocytes (MyoC), endothelial cells (EC1/EC2), and macrophages (Macro). In addition, human BAT contained two mixed stromal populations comprising preadipocytes and fibroblasts (Pread/Fibro) and chondrocyte- and fibroblast-like cells (Chondro/Fibro), as well as a substantial immune compartment including T cells, class-switched memory B cells, and dendritic cells (Figure 1B and Suppl. Fig. 1B; Extended File 2).

We detected 143 GPCRs in mouse BAT at room temperature (22 °C), 172 GPCRs after 14 days of cold exposure (8 °C), and 215 GPCRs in human BAT (Extended File 3). Notably, in mouse BAT, several GPCRs were not uniquely expressed in a single cell type. Specifically, in mouse BAT several GPCRs showed macrophage-enriched expression at 22 °C (e.g., *Cnr2, Gpr141, Gpr183, C5ar1,* and *Adgre4*) and at 8 °C (*Cnr2, Cxcr5, Gpr171, Gpr183,* and *C3ar1*) (Figure 1C and Extended File 4). In human BAT, immune CD4^+^ T cells exhibited the highest number of cell-type–specific GPCRs, including *CHRM2, GPR139, GPR149, LPAR3, RXFP2, GHRHR, GRM4,* and *GPRC5D* (Figure 1D and Extended File 4).

To benchmark our dataset, we compared the detected GPCRs with previously reported profiles from total mouse BAT [36] and from isolated brown adipocytes and preadipocytes [37]. While some GPCRs expressed at very low levels—such as *Gpr3*—were not captured in our dataset, we recovered the vast majority of receptors previously identified in those studies (Extended File 5). The broad coverage and overlap with prior qPCR and bulk RNA-seq analyses underscore the sensitivity and specificity of our single-nucleus RNA-seq approach.

Finally, we classified the detected GPCRs according to standard GPCR family nomenclature into Class A, Class B, Class C, Frizzled, and Adhesion GPCRs (aGPCRs). Across both mice and human BAT, Class A GPCRs were predominant (Figure 1E and Extended File 3), followed by aGPCRs, which represented the second most abundant GPCR family (Fig. 1E).

### 3.2 Cellular distribution and dynamic regulation of adhesion GPCRs in mouse brown adipose tissue upon cold exposure

**Figure 2:**
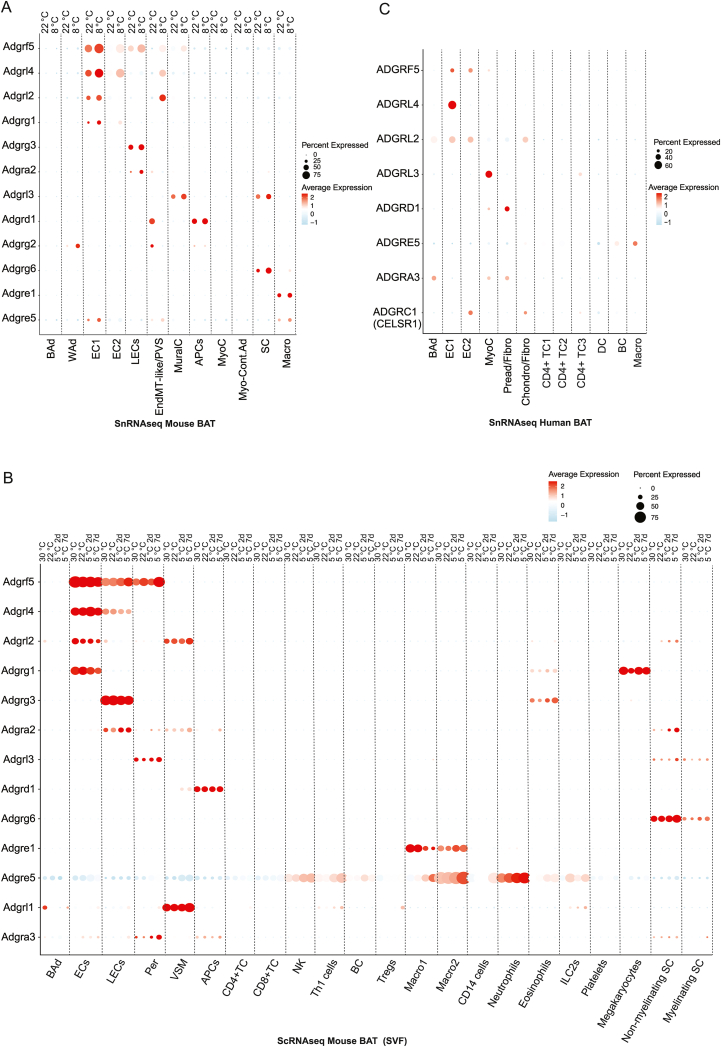
**Cellular distribution and cold-associated regulation of adhesion GPCRs in mouse and human brown adipose tissue.** (A) Dot plot showing expression of adhesion GPCRs (aGPCRs) across major cell populations in mouse BAT, profiled by single-nucleus RNA sequencing (snRNA-seq) at room temperature (22 °C) and after prolonged cold exposure (8 °C). (B) Dot plot depicting aGPCR expression across cell populations in a published mouse BAT single-cell RNA sequencing (scRNA-seq) dataset generated from the stromal vascular fraction (SVF). (C) Dot plot showing aGPCR expression across cell populations in human BAT snRNA-seq data. Dot size indicates the percentage of cells expressing each gene, and color intensity represents average normalized expression. Per: Pericytes, VSM: vascular smooth muscle cells, NK: NK cells, BC: B cells.

Of the 33 members of the aGPCR family, we detected 24 aGPCRs, using a permissive detection threshold (expression in ≥1% of nuclei within at least one cell population), indicating broad representation of this receptor family in mouse BAT (Fig. 1E). To prioritize receptors with robust and cell type–relevant expression for downstream analyses, we subsequently applied a more stringent cutoff, requiring expression in ≥30% of nuclei within a given cell population. Using this criterion, 12 aGPCRs were retained for detailed analysis (Figure 2A).

Among these, *Adgrf5* and *Adgrl4* were the most highly expressed and were restricted to vascular compartments, with peak signal in endothelial cells (ECs) and EndMT-like/PVS subclusters. *Adgrl2* and *Adgrg1* showed similar vascular-biased distributions, but lower level of expression. *Adgrg3* and *Adgra2* were enriched in lymphatic ECs (LECs), whereas *Adgrd1* localized to adipogenic progenitor cells (APCs), consistent with its reported role in regulating preadipocyte adipogenic potential in white adipose tissue [40] (Fig. 2A).

To explore whether aGPCR–expressing cell populations might also participate in intercellular communication within the BAT microenvironment, we performed CellChat-based ligand–receptor inference analyses, which are summarized in Suppl. Fig. 2. These analyses indicated that vascular and stromal cell populations constitute prominent communication hubs in BAT, particularly during cold exposure (Suppl. Fig. 2A–B), engaging extensively through cell–cell contact and ECM–receptor signaling categories (Suppl. Fig. 2C). Several predicted cold-associated interactions involved aGPCR family members, with a notable enrichment within vascular cell populations, including endothelial and mural cells, across non-adipocyte lineages (Suppl. Fig. 2D). However, because aGPCRs primarily function as mechanosensors and integrators of cell–ECM interactions, rather than classical ligand–receptor signaling receptors, CellChat-based predictions were not used as a criterion for receptor candidate prioritization.

To assess modality and dataset consistency, we compared our snRNA-seq findings to a published mouse BAT single-cell RNA-seq (scRNA-seq) dataset from the stromal vascular fraction (SVF) [41]. We detected 13 aGPCRs in scRNA-seq and observed high concordance in both expression levels and cell-type assignments across the two platforms (Fig. 2B). The scRNA-seq dataset contained proportionally more immune cells than our snRNA-seq, likely reflecting differences in SVF isolation and nuclei vs. whole-cell capture. In human BAT snRNA-seq, we identified 8 aGPCRs; *ADGRF5*, *ADGRLl4*, *ADGRL2*, *ADGRL3*, *ADGRD1*, and *ADGRE5*, which overlapped with mouse BAT and showed strong cross-species agreement in cell-type distribution and relative abundance, whereas *ADGRC1* (*CELSR1*) was specific to human BAT in our datasets (Fig. 2C). Overall, aGPCR expression patterns were conserved across species and robust to nuclei vs cell profiling.

Because cold exposure induces profound vascular remodeling in BAT, including endothelial state transitions and extracellular matrix reorganization, we prioritized aGPCRs that were highly expressed in vascular compartments and dynamically regulated during cold adaptation. Among the vascular-enriched aGPCRs identified, *Adgrf5*, *Adgrl4*, and *Adgrl2* emerged as leading candidates. Prior studies have established pro-remodeling and angiogenesis-associated roles for *Adgrl4(Eltd1)* [42], and context-dependent endothelial functions for *Adgrl2(Lphn2)* [43], whereas *Adgrf5(Gpr116)* has been primarily linked to endothelial barrier integrity and vascular patterning in non-metabolic tissues [44,45]. Notably, *Adgrf5(Gpr116)* displayed conserved endothelial expression across organs (as assessed using publicly available single-cell atlases, including the Tabula Muris resource) and robust induction in cold-exposed BAT ECs and EndMT-like cells in our datasets, suggesting a potential role in fine-tuning vascular remodeling rather than serving as an essential driver of vessel growth. Consistent with this interpretation, genetic interaction between ADGRF5 and ADGRL4 during development reveals that while combined loss is perinatal-lethal, single deletions are viable and endothelial-restricted double deletion does not impair viability [46], supporting a modulatory rather than indispensable role for ADGRF5 in vascular remodeling programs. Together with the availability of conditional alleles and established in-house models, these considerations motivated our focus on ADGRF5(GPR116) in the context of BAT vascular adaptation to cold, which we investigated functionally in the following sections.

### 3.3 Global deletion of ADGRF5(GPR116) attenuates thermogenic efficiency during prolonged cold adaptation

To determine whether ADGRF5(GPR116) plays a physiological role in cold-induced thermogenesis, we subjected global ADGRF5(GPR116) knockout mice to both acute and prolonged cold exposure.

**Figure 3:**
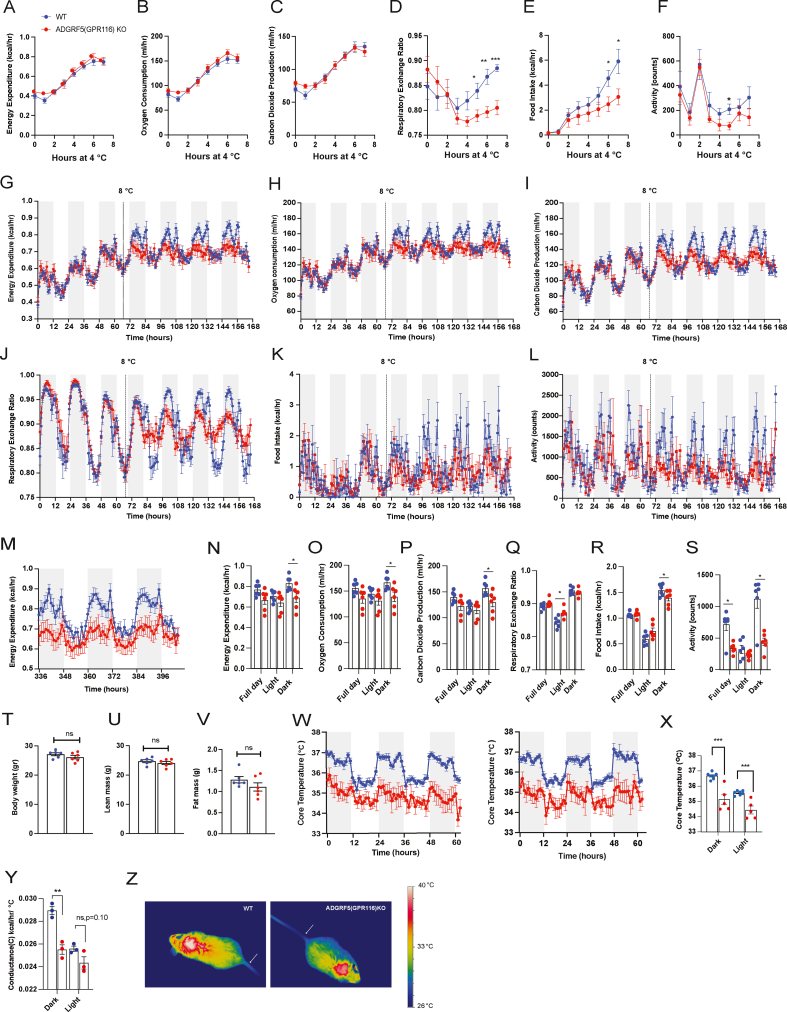
**Global deletion of ADGRF5(GPR116) impairs thermogenic energy expenditure during prolonged cold exposure**. (A–F) Indirect calorimetry measurements of energy expenditure, oxygen consumption, carbon dioxide production, respiratory exchange ratio (RER), food intake, and locomotor activity in ADGRF5(GPR116) knockout (KO) mice and wild-type (WT) controls during acute cold exposure at 4 °C for 8 h. Mice were male C57BL6N, 9–10 weeks old (n = 5–6 per group). (G–L) Indirect calorimetry during cold acclimation, in which ambient temperature was gradually reduced from 22 °C to 8 °C over 7 days (2 °C every 12 h). The last 3 days of the temperature ramp are shown, followed by maintenance at 8 °C for the indicated duration. (M−S) Indirect calorimetry measurements during prolonged cold exposure at 8 °C for 14 days. Data shown represent the last 3 days of cold exposure. Mice were male C57BL6N, 11–12 weeks old (n = 6 per group). (T–V) Body composition parameters (body weight, lean mass, and fat mass) measured at the end of 14 days at 8 °C. (W) Continuous recordings of core body temperature over 3 days at beginning of cold exposure 8 °C (left) and 3 last days at the end of 14 days (right) of cold exposure at 8 °C. (X) Mean core body temperature and (Y) thermal conductance calculated from the same 3-day period at the end of 14 days of cold exposure at 8 °C. (Z) Representative infrared thermography images of WT and ADGRF5(GPR116)KO mice. The arrow indicates a position 0.5 cm from the base of the tail. All mice were single-housed. Grey shaded areas indicate the dark phase. Statistical analyses for indirect calorimetry data were performed using CalR, with group differences assessed using general linear models, with or without ANCOVA where appropriate to account for body weight or lean mass differences, as implemented in the CalR platform. Body composition parameters, mean core body temperature, and thermal conductance were analyzed using unpaired two-tailed Student's t test. Data are presented as mean ± SEM. ∗p < 0.05, ∗∗p < 0.01, ∗∗∗p < 0.001.

During an acute cold challenge (4 °C for 8 h), WT and ADGRF5(GPR116)KO mice exhibited comparable increases in energy expenditure (EE), oxygen consumption (VO_2_), and carbon dioxide production (VCO_2_), indicating an intact rapid thermogenic response (Figure 3A–C). In contrast, knockout mice displayed a lower respiratory exchange ratio (RER) and a trend toward reduced food intake and locomotor activity during acute cold exposure (Figure 3D–F).

Under prolonged cold exposure (8 °C for 14 days), ADGRF5(GPR116)KO mice progressively diverged from WT controls, exhibiting impaired cold adaptation that became apparent after transition to sustained cold and persisted throughout the exposure period. Statistical analyses were therefore performed on averaged values from the final days of cold exposure, corresponding to the stabilized maintenance phase of thermogenesis. Specifically, knockout mice showed significantly reduced EE, VO_2_, and VCO_2_, particularly during the dark (active) phase (Figure 3G–I, M−P). RER was largely comparable between genotypes during the dark phase but was modestly elevated during the light phase in knockout mice (Figure 3J,Q). Food intake was slightly reduced during the dark phase, and locomotor activity was markedly lower in knockout mice during the dark phase (Fig. 3K–L, R–S). These differences were independent of body weight, lean mass, and fat mass, which were comparable between genotypes after 14 days of cold exposure (Figure 3T–V).

Consistent with these findings, ADGRF5(GPR116)KO mice exhibited a significantly reduced core body temperature during prolonged cold exposure while maintaining circadian rhythmicity (Figure 3W–X). Core temperature remained near the hypothermia threshold during both dark and light phases, indicating impaired thermogenic efficiency. Calculation of thermal conductance revealed no increase in heat loss; instead, conductance was modestly reduced in knockout mice during the dark phase and comparable between genotypes during the light phase (Fig. 3Y), consistent with reduced heat production rather than enhanced heat dissipation. Infrared thermography qualitatively revealed reduced heat emission from the interscapular region in knockout mice, with no apparent increase in heat loss from the tail or distal extremities (Fig. 3Z).

The impaired thermogenic phenotype was also observed in female ADGRF5(GPR116)KO mice (Suppl. Fig. 3A–C), whereas heterozygous mice and knockout mice housed at room temperature (22 °C) showed no differences in energy expenditure, substrate utilization, food intake, or locomotor activity (Suppl. Fig. 3D–L). As reported previously, homozygous knockout mice displayed increased heart and spleen mass [44] (Suppl. Fig. 3M).

Together, these results demonstrate that global deletion of ADGRF5(GPR116) does not impair the rapid initiation of cold-induced thermogenesis but leads to an inability to mount and sustain a full thermogenic response during prolonged cold exposure.

### 3.4 ADGRF5(GPR116) is essential for acquiring cold remodeled adipocyte identity

During prolonged cold exposure, classical BAT constitutes the primary site of non-shivering thermogenesis, while inguinal iWAT contributes to adaptive heat production through the recruitment of beige thermogenic adipocytes. Because global ADGRF5(GPR116)KO mice exhibited impaired thermogenic efficiency during prolonged cold exposure, we next sought to determine how loss of ADGRF5(GPR116) affected adipocyte remodeling across these thermogenic depots. To this end, we performed snRNA-seq of BAT and iWAT from WT and ADGRF5(GPR116)KO mice maintained at room temperature (22 °C) or exposed to prolonged cold (8 °C, 14days).

Across both depots, snRNA-seq resolved the expected major parenchymal and stromal populations, including adipocytes, endothelial and mural cells, Schwann cells, and immune cells (Suppl. Fig. 4 and Extended File 6). Among all cell types, adipocytes displayed the most pronounced and consistent genotype-dependent transcriptional remodeling during cold exposure, whereas non-adipocyte populations showed comparatively modest shifts (Suppl. Figs. 4D and 4H and Suppl. Tables 2 and 3). This observation prompted us to focus subsequent analyses on adipocyte identity and state. At the population level, KO BAT already exhibited altered adipocytes composition at room temperature, with a redistribution away from canonical brown adipocytes toward alternative adipocyte states. During prolonged cold exposure, this imbalance became more pronounced: brown adipocytes expanded to ∼59% of nuclei in WT BAT, but reached only ∼42% in KO BAT, whereas white-like adipocytes (WAd) increased disproportionately in KO (from ∼2.6% in WT to ∼8% in KO) (Suppl. Fig. 4D and Suppl. Table 2). In parallel, KO BAT showed a marked expansion of Myogenic-contractile adipocytes (Myo-Cont.Ad), increasing from ∼2 to 3% in WT to ∼16 % at RT and remaining elevated during cold exposure (∼1.3% in WT to ∼5% in KO) (Suppl. Fig. 4D and Suppl. Table 2). These data indicate that KO BAT does not undergo adipocyte loss, but instead accumulates adipocytes adopting alternative, thermogenically inefficient states.

A related yet depot-specific imbalance was observed in iWAT. During prolonged cold exposure, beige adipocytes (UCP1+) expanded from ∼14% in WT to ∼46% in KO, accompanied by a reduction in white adipocytes (from ∼49% to ∼28%), revealing an exaggerated accumulation of UCP1-expressing adipocytes in KO iWAT (Suppl Fig. 4H and Suppl. Table 3).

To determine whether these transcriptional changes were accompanied by gross structural alterations, we performed histological and morphometric analyses of different fat depots; BAT, iWAT, and gonadal white adipose tissue (gWAT). H&E staining revealed no evidence of adipocyte loss or gross structural abnormalities across depots in ADGRF5(GPR116)KO mice compared to WT controls following prolonged cold exposure. In BAT, KO mice displayed less well-defined brown adipocyte borders in KO vs WT upon cold exposure (Suppl. Fig. 5A). In iWAT, KO mice exhibited fewer multilocular beige-like adipocytes compared to WT under cold, whereas gWAT did not show obvious genotype-dependent differences (Suppl. Fig. 5A). After cold exposure, BAT and iWAT weights did not differ between genotypes, while gWAT appeared smaller in KO vs WT (Suppl. Fig. 5B). Quantification of lipid content and adipocyte size distributions revealed no significant genotype-dependent differences across depots (Suppl. Fig. 5C–G). Moreover, in vitro differentiation of SVF from KO and WT mice yielded comparable adipogenic capacity (Suppl. Fig. 5H), supporting intact adipocyte differentiation potential in the global KO. These findings indicate that impaired thermogenesis in ADGRF5(GPR116)KO mice is not driven by adipocyte loss or gross lipid storage defects.

**Figure 4:**
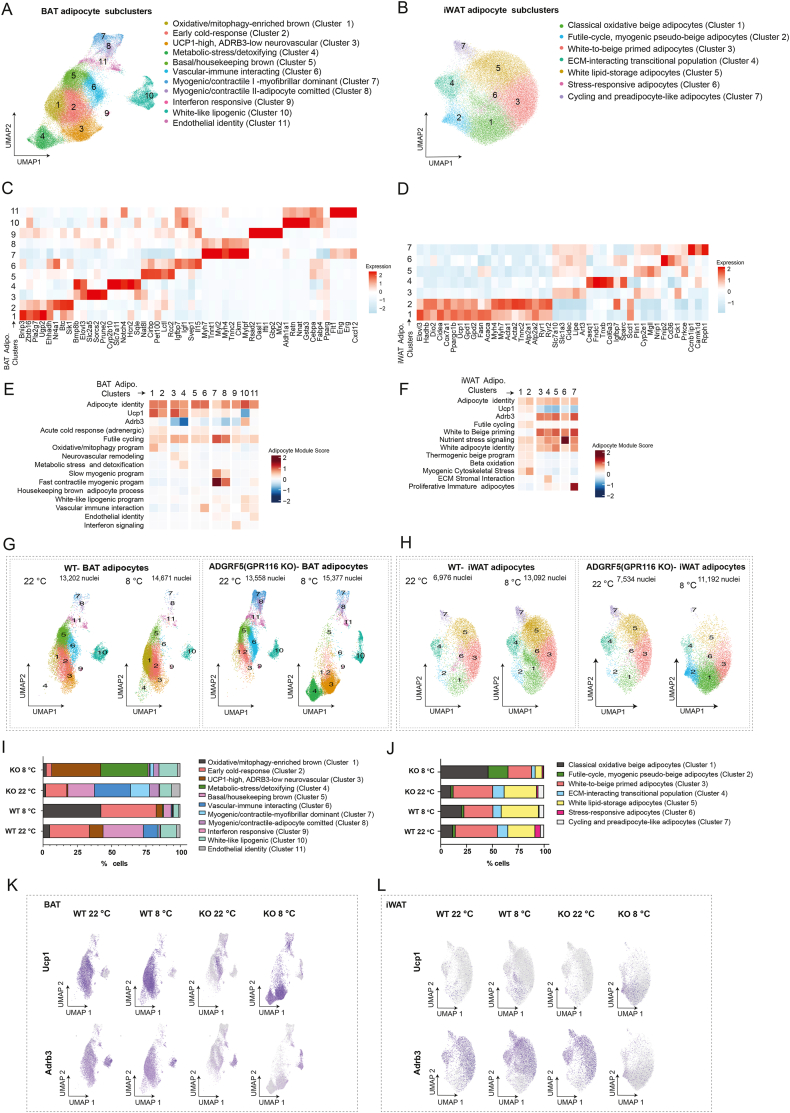
**Single-nucleus transcriptomics reveals depot-specific adipocyte subpopulations in BAT and iWAT across cold exposure and ADGRF5(GPR116) deletion.** (A–B) UMAP visualization of adipocyte subclusters identified by single-nucleus RNAseq in BAT (A) and iWAT (B). Clusters are colored and numbered/annotated based on transcriptional signatures. (C–D) Heatmaps showing scaled expression of representative marker genes used to annotate adipocyte subclusters in BAT (C) and iWAT (D). Genes are ordered by cluster specificity. (E–F) Module score analysis summarizing key adipocyte functional programs across BAT (E) and iWAT (F) subclusters. (G–H) UMAP projections of adipocyte subclusters in BAT (G) and iWAT (H). Nuclei numbers for each condition are indicated. (I–J) Quantification of adipocyte subcluster composition in BAT (I) and iWAT (J) across genotypes and temperatures, shown as the percentage of cells per cluster. (K–L) Feature plots showing expression of canonical thermogenic markers *Ucp1* and *Adrb3* across adipocyte populations in BAT (K) and iWAT (L) under all experimental conditions.

To further dissect adipocyte heterogeneity, we reclustered adipocytes from BAT and iWAT, resolving eleven transcriptionally distinct adipocyte subclusters in BAT and seven in iWAT (Figure 4A–B). Detailed marker gene profiles (Extended File 7) and module-scoring–based annotation (Suppl. Table 4), for of each adipocyte subcluster are shown in Figure 4C–F and detailed description on annotation is provided in the Supplementary Results (Section S3.4). These subclusters encompassed oxidative brown and beige adipocytes, early cold-response adipocytes, UCP1-high but adrenergically attenuated neuro-vascular remodeling adipocytes, metabolic-stress/detoxifying adipocytes, white-like lipogenic adipocytes, and multiple myogenic/futile-cycle adipocyte states.

In WT BAT, cold exposure drove a robust thermogenic remodeling response, with oxidative/mitophagy-enriched brown adipocytes increasing (from ∼5.0% at room temperature to ∼42.4% in cold) together with an increase in early cold-response adipocytes (from ∼28.9% to ∼40.3%). In contrast, ADGRF5(GPR116)KO BAT failed to expand oxidative/mitophagy-enriched brown adipocytes, which remained severely reduced (∼2.1–2.5%). Instead, KO BAT accumulated UCP1-high but Adrb3-low neuro-vascular remodeling adipocytes, which increased dramatically from ∼0.8% to ∼35.7%, along with elevated myogenic/futile-cycle adipocytes. This shift reflects an adrenergically uncoupled and thermogenically inefficient adipocyte state (Figure 4G,I and Suppl. Table 5).

Similarly, WT iWAT responded to cold by expanding classical oxidative beige adipocytes (from ∼11.9% to ∼20.4%). Although KO animals showed and exaggerated expansion of classical oxidative beige adipocytes (from ∼10.2% to ∼46.2%) and accumulated white-to-beige primed adipocytes, these cells did not upregulate *Adrb3* and therefore remained thermogenically incompetent (Fig. 4F). Instead, KO iWAT showed a strong increase in futile-cycle/myogenic pseudo-beige adipocytes (from ∼2.2% to ∼19.3%), accompanied by a reduction in ECM-interacting, cycling, and white adipocyte populations (Figure 4H,J and Suppl. Table 6).

Together, these analyses demonstrate that ADGRF5(GPR116) is essential for the acquisition of adrenergically competent, oxidative thermogenic adipocyte identity in both BAT and iWAT during cold adaptation. In its absence, adipocytes divert into Adrb3-low (Fig. 4K-L), thermogenically inefficient states dominated by UCP1-independent, ATP-consuming futile-cycle and myogenic programs. These adipocyte state changes provide a cellular explanation for the inability of ADGRF5(GPR116)KO mice to mount a full thermogenic response during prolonged cold exposure.

### 3.5 Non-adipocyte deletion of ADGRF5(GPR116) limits adrenergic thermogenic activation despite intact adipocyte-autonomous function

**Figure 5:**
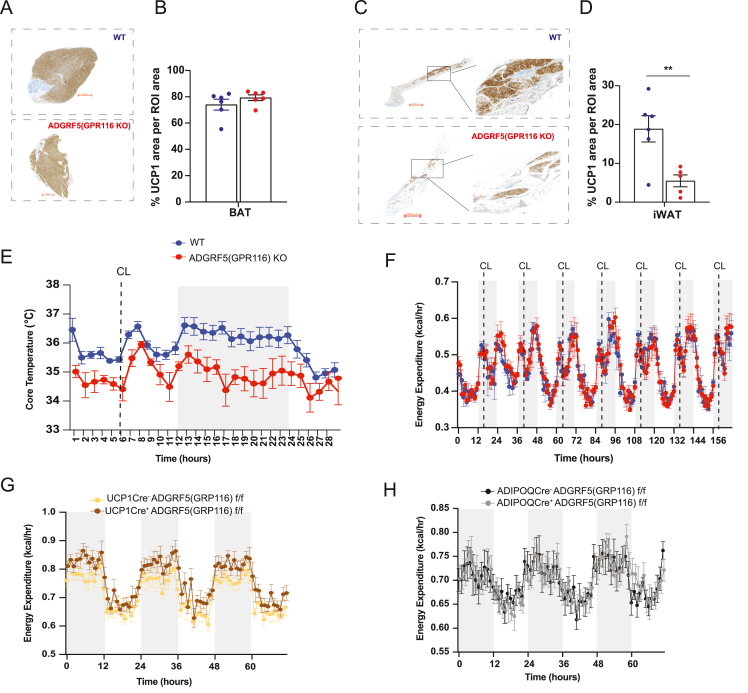
**Adipocyte-autonomous thermogenic capacity is preserved upon ADGRF5(GPR116) deletion.** (A) Representative UCP1 immunohistochemistry of BAT from WT and ADGRF5(GPR116)KO mice following cold exposure (8 °C, for 14 days). (B) Quantification of UCP1-positive area in BAT. (C) Representative UCP1 immunohistochemistry of iWAT from WT and ADGRF5(GPR116)KO mice after cold exposure (8 °C, for 14 days), with magnified insets highlighting UCP1-positive regions. (D) Quantification of UCP1-positive area in iWAT. (E) Core body temperature of WT and ADGRF5(GPR116)KO mice, housed at 8 °C for 14 days and given an single i.p injection of CL-316,243, 2 mg/kg on day 12, at 8 °C. n = 6, WT and n = 5, ADGRF5(GPR116)KO mice. (F) Energy expenditure measured by indirect calorimetry during repeated injections of CL (CL-316,243, 2 mg/kg), WT (n = 4) and ADGRF5(GPR116)KO mice (n = 5), housed at room temperature, 22 °C. (G) UCP1-Cre–driven ADGRF5(GPR116) deletion, n = 5, Ucp1Cre^−^ADGRF5(GPR116)f/f and n = 6, Ucp1Cre^+^ADGRF5(GPR116)f/f mice. and (H) Adipoq-Cre–driven ADGRF5(GPR116) deletion, n = 6, AdipoqCre^−^ADGRF5(GPR116)f/f and n = 6, AdipoqCre^+^ADGRF5(GPR116)f/f mice. Grey shaded areas on graphs indicate the Dark phase. Mice were single-caged. Mice were males, 11–12 weeks old. Data are shown as mean ± SEM; each dot represents one mouse. Statistics were analyzed using unpaired two-tailed Student's t test, ∗∗p < 0.01.

The transcriptional analyses revealed that loss of ADGRF5(GPR116) prevented adipocytes from acquiring fully competent thermogenic identities during cold remodeling. Consistent with the transcriptional shift toward Adrb3-low, thermogenically incompetent adipocyte states in KO mice, BAT UCP1 positive area remained unchanged (Figure 5A–B), while iWAT displayed markedly reduced UCP1 induction (Figure 5C–D). Functionally, KO animals exposed to cold (8 °C) for 12 days were unable to maintain core body temperature, and an acute β_3_-adrenergic stimulation, with the specific agonist, CL-316,243 (CL), during cold exposure elicited only a minimal, transient response in KO mice, failing to restore thermogenesis (Fig. 5E).

To test whether the impaired thermogenic response reflected an adipocyte-autonomous defect or instead arose from the cold-induced tissue microenvironment, we bypassed endogenous sympathetic signaling by administering daily injections of the β_3_-adrenergic agonist CL316,243 to mice housed at room temperature. Under these non-cold conditions, WT and KO mice exhibited indistinguishable increases in energy expenditure (Fig. 5F), as well as comparable VO_2_, VCO_2_, and RER responses (Suppl. Fig. 6A–E), demonstrating that adipocyte-intrinsic adrenergic signaling capacity is preserved, when the defective cold-induced microenvironment is removed.

To determine whether impaired sympathetic input could account for the phenotype observed during cold exposure, we assessed circulating and tissue norepinephrine (NE) levels and examined sympathetic innervation. Circulating and tissue NE content and tyrosine hydroxylase (TH) immunoblotting were comparable between genotypes (Suppl. Fig. 7A–C), excluding defective neuronal drive as a cause of the hypothermia. Moreover, circulating thyroid hormone (T3) levels—an essential systemic regulator of thermogenesis—were unchanged between genotypes (Suppl. Fig. 7D). Serum lipid profiles were also similar (Suppl. Fig. 7E–G), further supporting intact systemic metabolic responses to cold. Importantly, serum lactate levels were not elevated in ADGRF5(GPR116)KO mice compared to WT controls (Suppl. Fig. 7H), indicating preserved systemic oxygenation and absence of hypoxia. This is consistent with prior reports showing that pulmonary dysfunction in ADGRF5(GPR116)KO mice manifests only after 18 weeks of age [21], whereas all cold-exposure experiments in this study were performed well before this time point (9–12 weeks old mice).

To directly test whether adipocyte-intrinsic ADGRF5(GPR116) contributes to thermogenic capacity, we generated brown adipocyte–specific (Ucp1-Cre) and pan-adipocyte (Adipoq-Cre) KO models. Neither model displayed defects in EE or metabolic responses during cold exposure (Figure 5G–H and Suppl. Fig. 6F-U), confirming that adipocyte-intrinsic Adgrf5(Gpr116) is dispensable for thermogenic activation.

Collectively, these data suggest that prolonged cold exposure in ADGRF(GPR116)KO mice leads to maladaptive tissue remodeling that impairs the brown adipocyte microenvironment, ultimately limiting full thermogenic activation, despite intact neuroendocrine signaling and preserved adipocyte-intrinsic adrenergic responsiveness.

### 3.6 Loss of ADGRF5(GPR116) induces maladaptive endothelial transcriptional reprogramming

Because ADGRF5(GPR116) was identified as a vascular-enriched aGPCR and prioritized from our GPCR screen (Fig. 2), we next examined how its loss affects endothelial cell states during cold-induced adipose tissue remodeling. To this end, we isolated vascular-lineage nuclei from BAT and performed high-resolution reclustering to resolve endothelial, mural, and perivascular cell states during prolonged cold exposure.

Reclustering of BAT vascular-lineage nuclei identified multiple transcriptionally distinct populations encompassing arterial, venous, capillary, lymphatic, mural, and mesenchymal-transition–associated states, consistent with the known heterogeneity of the adipose vasculature. Detailed cluster annotation and canonical marker expression are provided in Suppl. Fig. 8, Suppl. Results S3.6 and Extended File 8. Importantly, loss of ADGRF5(GPR116) did not give rise to novel endothelial populations, but instead resulted in genotype-dependent redistribution and transcriptional remodeling within pre-existing endothelial states.

**Figure 6:**
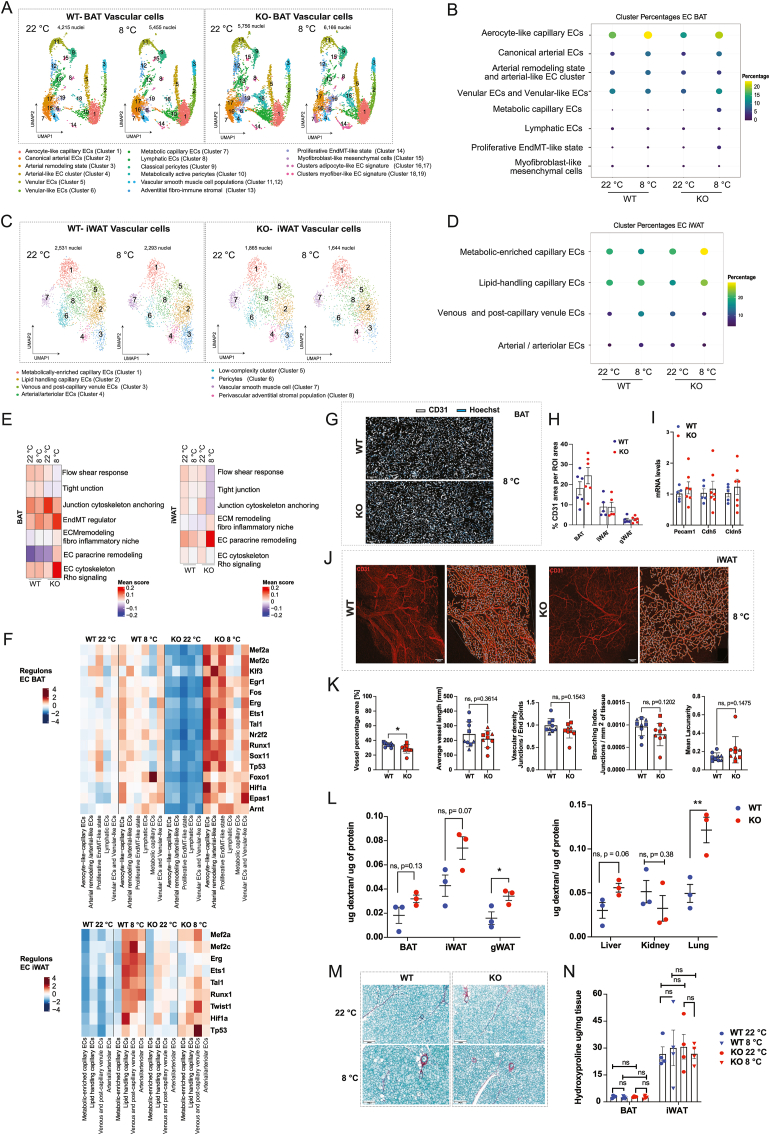
**Endothelial reprogramming in BAT and iWAT upon ADGRF5(GPR116) deletion.** (A) UMAP representation of vascular cell populations from BAT of WT and ADGRF5(GPR116)KO mice housed at 22 °C or 8 °C (14 days), based on integrated single-nucleus RNA-seq analysis. (B) Dot plot showing relative proportions of endothelial subpopulations in BAT across genotypes and temperatures. Dot size indicates the percentage of cells per condition. (C) UMAP representation of vascular and perivascular cell populations from iWAT of WT and ADGRF5(GPR116)KO mice at 22 °C and 8 °C (14 days), highlighting depot-specific differences in endothelial organization compared with BAT. (D) Dot plot showing relative proportions of major endothelial subtypes in iWAT across genotypes and temperatures. Nuclei numbers for each condition are indicated. (E) Module score analysis of endothelial functional programs in BAT (left) and iWAT (right), comparing WT and KO conditions. (F) Heatmaps showing transcription factor regulon activity in BAT (top) and iWAT (bottom) endothelial subtypes across genotypes and temperatures. (G) Representative immunofluorescence images of BAT sections stained for CD31 (endothelial cells) and Hoechst in WT and ADGRF5(GPR116)KO mice under cold exposure. (H) Quantification of CD31-positive area normalized to total region of interest (ROI) area, in BAT, iWAT, and gonadal WAT (gWAT), (I) qPCR analysis of endothelial marker expression (*Pecam1*, *Cdh5,* Cldn5) in whole BAT tissue, from WT and ADGRF5(GPR116)KO mice, exposed to 8 °C for 14 days. Dots indicate individual mice. (J) Whole-mount CD31 immunostaining of iWAT vasculature from WT and ADGRF5(GPR116)KO mice following cold exposure, illustrating vascular network organization. (K) Quantitative analysis of vascular morphology parameters. Each symbol represents a different mouse. (L) FITC–dextran permeability assay showing endothelial barrier function in BAT, iWAT, gWAT, liver, kidney, and lung of WT and ADGRF5(GPR116)KO mice. (M) Representative Sirius Red/Fast Green–stained sections of BAT from WT and ADGRF5(GPR116)KO mice at 22 °C and 8 °C (14days). (N) Quantification of hydroxyproline content in BAT and iWAT, indicating total collagen content across genotypes and temperatures. Data are shown as mean ± SEM. Statistical analyses were performed using unpaired two-tailed Student's t test, Statistical significance is indicated as ∗ p < 0.05, ∗∗p < 0.01, otherwise, differences are not significant (ns).

To assess how loss of ADGRF5(GPR116) alters vascular cell composition during cold adaptation, we quantified changes in vascular subcluster abundance across genotypes and temperatures in BAT. Genotype-dependent remodeling was largely confined to endothelial populations, whereas mural and perivascular cells exhibited only modest changes in abundance (Suppl. Table 7). The most pronounced genotype-dependent effect was observed in the proliferative EndMT-like state, which remained rare in WT BAT across temperatures (∼0.6%) but expanded markedly in KO mice during cold exposure (3.4%). Notably, the myofibroblast-like mesenchymal cells did not show a corresponding expansion, suggesting that endothelial reprogramming in the absence of ADGRF5(GPR116) may preferentially engage early EndMT-like states, rather than overt fibrotic cell accumulation. In addition, venular and venular-like ECs (Clusters 5 and 6, combined) were increased in KO mice during cold exposure, whereas arterial remodeling and arterial-like EC cluster (Clusters 3 and 4, combined) were reduced, despite preserved cold-induced expansion of canonical arterial ECs. A metabolic capillary EC population, nearly absent in WT BAT, emerged selectively in KO mice during cold exposure. In contrast, pericytes, vascular smooth muscle cells, and other mural populations varied by less than ∼1–2% between genotypes, indicating that loss of ADGRF5(GPR116) primarily affects endothelial, rather than mural, vascular composition (Figure 6A–B and Suppl. Table 7).

To determine whether endothelial remodeling upon loss of ADGRF5(GPR116) extends beyond thermogenic adipose tissue, we performed an analogous high-resolution reclustering of vascular-lineage nuclei from iWAT. This analysis resolved eight transcriptionally distinct vascular and perivascular populations, encompassing capillary, venous, arterial, mural, and adventitial stromal states, consistent with the known vascular organization of white adipose tissue. Detailed cluster annotation, marker gene expression, and integrated visualization are provided in Suppl. Fig. 8, Suppl. Results S3.6, and Extended File 8. Notably, and in contrast to BAT, no discrete endothelial-to-mesenchymal transition (EndMT)-like populations were detected in iWAT (Figure 6C–D).

Quantification of vascular subcluster abundance in iWAT revealed genotype-dependent endothelial redistribution during cold exposure, although with a pattern distinct from BAT (Suppl. Table 8). In WT mice, cold exposure induced a marked expansion of venous and post-capillary venule ECs, increasing from 7.23% at RT to 15.13% in cold, alongside an expansion of arterial/arteriolar ECs from 3.75% to 7.15%, consistent with recruitment of venous and arterial programs during cold-associated vascular remodeling. In contrast, ADGRF5(GPR116)KO mice failed to mount this venous and arterial enrichment. Venous and post-capillary ECs decreased during cold exposure (11.26% at RT to 5.66% in KO cold), and arterial/arteriolar ECs were similarly reduced (6.38%–4.01%). Instead, KO mice exhibited a redistribution toward capillary endothelial states, with metabolically enriched capillary ECs expanding from 20.54% at RT to 28.47% in KO cold, and lipid-handling capillary ECs increasing from 16.41% to 23.54%. Thus, loss of ADGRF5(GPR116) in iWAT promoted a cold-associated shift toward capillary endothelial predominance, without induction of EndMT-like endothelial states. These findings highlight depot-specific endothelial remodeling responses to ADGRF5(GPR116) loss, with BAT exhibiting endothelial state destabilization and early EndMT-like transitions, whereas iWAT displayed altered endothelial balance without overt endothelial lineage conversion.

To functionally interpret these genotype-dependent endothelial changes, we applied pathway and module scoring to assess endothelial state programs during cold adaptation. In BAT, loss of ADGRF5(GPR116) was associated with reduced activity of flow- and shear-responsive programs, diminished junctional stability signatures, and increased cytoskeletal stress and Rho GTPase–associated signaling. In parallel, mesenchymal transition–associated programs (EndMT regulator) were selectively induced, consistent with the expansion of EndMT-like endothelial states. In iWAT, ADGRF5(GPR116) loss primarily enhanced extracellular matrix remodeling and fibro-inflammatory niche programs. Across both depots, endothelial paracrine and secretory programs were increased, suggesting altered angiocrine communication (Figure 6E, Suppl. Table 9)

Because these pathway-level changes imply coordinated transcriptional control, we next examined transcription factor regulon activity to identify regulatory programs underlying ADGRF5(GPR116)-dependent endothelial remodeling. In BAT regulon analysis revealed a genotype-dependent amplification of cold-induced regulatory activity, with knockout endothelial cells displaying broadly increased regulon activation at 8 °C compared with WT controls (Fig. 6F). This included induction of endothelial and flow-associated transcription factors (MEF2A/MEF2C, ERG, ETS1, KLF3), immediate-early response factors (EGR1, FOS), and stress- and transition-associated regulators (RUNX1, TP53, FOXO1), as well as hypoxia-responsive transcription factors (HIF1A, EPAS1, ARNT). Endothelial regulon responses in iWAT differed from those observed in BAT. In WT mice, cold exposure was associated with induction of several endothelial regulatory programs, including MEF2A/MEF2C, ERG, ETS1, and TAL1, although with generally lower magnitude compared with BAT (Fig. 6F). In ADGRF5(GPR116)-deficient iWAT, this cold-associated regulon activation was attenuated, with knockout endothelial cells showing reduced engagement of these programs across multiple endothelial states. In contrast, stress-associated and early mesenchymal reprogramming–linked regulons, including TP53 and TWIST1, remained detectable or were selectively induced in KO iWAT endothelial cells.

To validate transcriptional predictions at the tissue level, we first assessed vascular abundance and architecture. CD31 immunostaining revealed comparable vascular area in BAT between genotypes, indicating no gross difference in vascular density at the analyzed time point (Figure 6G–H). Consistent with this, qPCR analysis of canonical endothelial markers (*Pecam1*, *Cdh5*, and *Cldn5*) showed no significant differences in expression between WT and ADGRF5(GPR116)KO BAT (Fig. 6I). In iWAT and gWAT, section-based CD31 area measurements were not significantly altered (Fig. 6H), whereas whole-mount vascular network analysis revealed a modest reduction in vessel area fraction, in iWAT, in knockout mice; however, additional network parameters—including average vessel length, branching density, and lacunarity—were unchanged, arguing against overt vascular pruning or structural collapse (Figure 6J–K). Importantly, Ki67 staining revealed minimal endothelial proliferation in both depots at the time of tissue collection (after 14 days, at 8 °C) (Suppl. Fig. 9A). Given the near-background signal and lack of CD31 co-localization, Ki67 was not quantified and is presented as representative images, consistent with analysis during the post-angiogenic maintenance phase rather than active vascular expansion. Accordingly, CD31 measurements at this stage reflect endothelial density and vascular maintenance rather than ongoing vessel growth.

Consistent with transcriptional predictions of impaired barrier integrity, FITC–dextran assays revealed a reproducible trend toward increased vascular permeability in BAT and iWAT of knockout mice, and signficant increase in gWAT. In contrast, dextran leakage was significantly increased in the lung, consistent with the established role of ADGRF5(GPR116) in pulmonary endothelial barrier maintenance [44], while liver and kidney permeability were unaffected (Fig. 6L). These findings indicate tissue-selective endothelial barrier dysfunction rather than catastrophic vascular failure.

Despite induction of endothelial inflammatory and leukocyte-recruiting transcriptional programs, CD68 immunostaining revealed minimal macrophage accumulation in BAT across genotypes and temperatures (Suppl.Fig. 9B), consistent with single-nucleus RNA-seq showing no expansion of immune cell populations (Suppl. Fig. 4 and Suppl. Tables 2 and 3). Thus, endothelial activation occurred without overt immune cell infiltration at the tissue level.

Finally, although endothelial cells exhibited pro-fibrotic and mesenchymal-biased transcriptional reprogramming, Sirius Red/Fast Green staining (Fig. 6M) and hydroxyproline quantification (Fig. 6N) revealed no genotype differences in collagen deposition in BAT or iWAT. Together, these data demonstrate that loss of ADGRF5(GPR116) induces maladaptive endothelial remodeling characterized by loss of quiescent endothelial identity, partial junctional destabilization, and early EndMT-like transitions, without progression to overt tissue fibrosis or inflammation-driven tissue infiltration at the analyzed stage of cold exosure duraration.

### 3.7 Deletion of ADGRF5(GPR116) remodels endothelial intercellular communication and paracrine signaling during cold exposure

**Figure 7:**
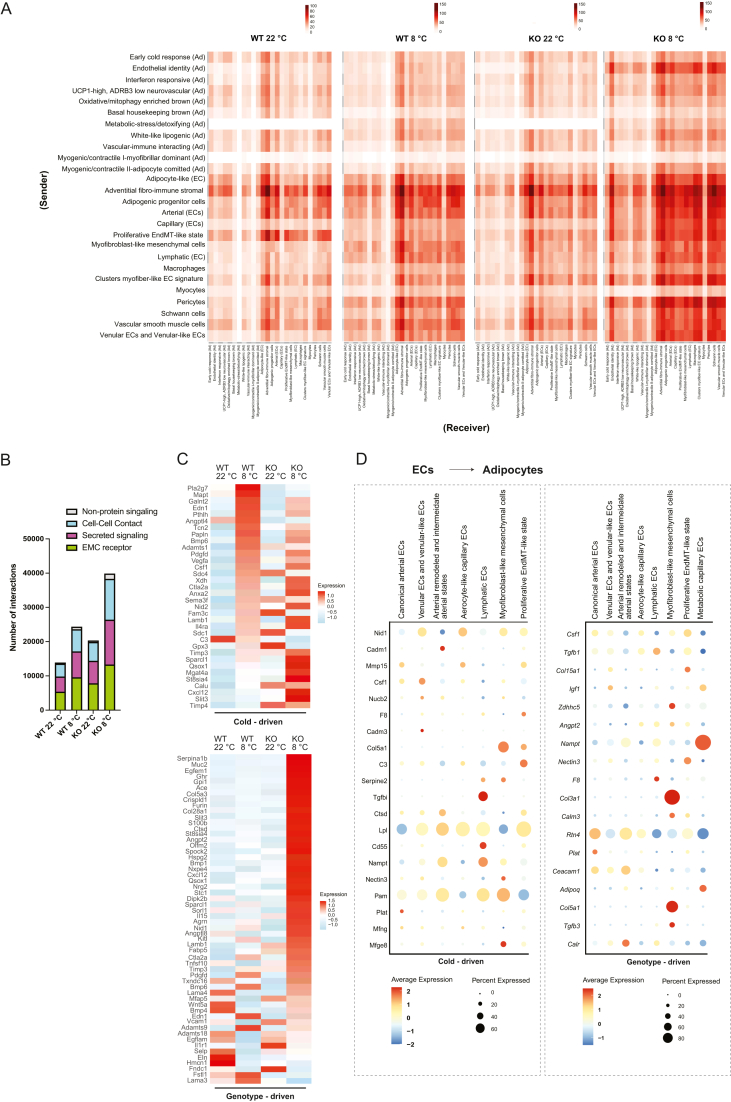
**Endothelial reprogramming upon ADGRF5(GPR116) deletion reshapes intercellular communication in BAT during prolonged cold exposure.** (A) CellChat interaction heatmaps showing predicted intercellular communication strength across major cell populations in BAT from WT and ADGRF5(GPR116) KO mice housed at room temperature (RT, 22 °C) or prolonged cold (8 °C, 14 days). Rows represent sender cell types and columns represent receiver cell types. Color intensity reflects aggregated communication probability across all signaling pathways. (B) Quantification of total predicted intercellular interactions in BAT under each condition, stratified by signaling mode (non-protein signaling, cell–cell contact, secreted signaling, and ECM–receptor interactions) (CellChat). (C) Heatmaps of differentially expressed endothelial-derived secreted and extracellular factors. Top: cold-driven endothelial factors induced in WT BAT upon cold exposure (WT 8 °C vs WT 22 °C). Bottom: genotype-driven endothelial factors selectively induced in ADGRF5(GPR116)KO BAT during cold exposure (KO 8 °C vs WT 8 °C). Factors were selected based on adjusted p < 0.05 and |log_2_FC| > 1 and ranked by expression change. (D) NicheNet analysis of endothelial-to-adipocyte communication. Dot plots show prioritized endothelial ligands predicted to regulate adipocyte gene programs. Left: cold-driven ligands. Right: genotype-driven ligands. Dot size reflects the fraction of expressing endothelial cells, and color indicates average ligand expression.

To investigate whether endothelial reprogramming induced by loss of ADGRF5(GPR116) alters intercellular communication within thermogenic adipose tissue, we performed CellChat analysis on the integrated single-nucleus RNA-seq dataset (Suppl. Fig. 10) from BAT across genotypes and temperatures. Under basal conditions (22 °C), overall communication strength and pathway distribution were comparable between WT and KO mice. In contrast, prolonged cold exposure markedly increased the number and diversity of predicted cell–cell interactions in ADGRF5(GPR116)KO BAT, exceeding those observed in WT cold (Figure 7A–B). This increase was driven predominantly by endothelial and stromal populations, which emerged as major signaling hubs in the KO cold condition, consistent with the transcriptional activation of angiocrine and secretory programs described above (Fig. 6E).

To further define endothelial-derived paracrine programs associated with ADGRF5(GPR116)-dependent remodeling, we analyzed secreted and extracellular factors differentially expressed in endothelial cells under prolonged cold exposure and/or ADGRF5(GPR116) deletion (adjusted p < 0.05; |log2FC| > 1). While cold exposure in WT BAT induced a shared set of endothelial angiocrine factors involved in vascular remodeling and tissue adaptation that were largely preserved in KO mice, ADGRF5(GPR116) deletion during cold exposure elicited a distinct endothelial secretory signature (Figure 7C, Extended File 9). KO cold endothelium selectively upregulated a broad spectrum of extracellular matrix components and matrix-modifying enzymes (*Col5a3*, *Col28a1*, *Lamb1*, *Nid1*, *Hspg2*, *Qsox1*, *Bmp1*, *Crispld1*, *Furin*, *Ctsd*), alongside vascular activation and permeability-associated mediators (*Angpt2*, *Ace*, *Cxcl12*), consistent with an enhanced remodeling and fibro-inflammatory angiocrine state. Notably, KO cold endothelium also showed prominent induction of axon–vascular guidance and neurovascular remodeling ligands, including *Slit3*, *Kitl*, *Olfm1*, and *Nrg2*. SLIT3 has been implicated in cold-induced neurovascular expansion and thermogenic adipose remodeling [47], while endothelial-derived KITL (SCF) has been shown to signal to brown adipocytes and regulate adipocyte state through c-Kit–dependent mechanisms [48].

To link altered endothelial communication to adipocyte transcriptional responses, we applied NicheNet analysis using endothelial subclusters as signal senders and adipocytes as receivers. This analysis identified a set of endothelial ligands whose predicted activity was selectively enhanced in ADGRF5(GPR116)KO mice during cold exposure, including *Csf1, Tgfb1*, *Igf1*, *Nectin3*, *Rtn4*, *Ceacam1*, and *Tgfb3* (Figure 7D, Suppl. Fig. 11). These ligands were predicted to regulate adipocyte gene programs associated with extracellular matrix organization, stress responses, and metabolic adaptation, consistent with the altered adipocyte states observed in knockout mice.

Collectively, these results indicate that loss of ADGRF5(GPR116) promotes a qualitatively distinct endothelial secretory and regulatory landscape, enriched for extracellular matrix remodeling, neurovascular guidance, and niche-patterning cues, thereby providing a framework through which endothelial reprogramming may reshape adipocyte identity during prolonged cold adaptation. Intercellular communication analysis was not further expanded for iWAT, as we identified only a very limited number of cold- and genotype-dependent secreted signaling interactions in this depot, in contrast to BAT; these analyses are therefore presented in Extended File 9.

### 3.8 Inducible endothelial deletion of ADGRF5(GPR116) impaired maintenance of the thermogenic response

**Figure 8:**
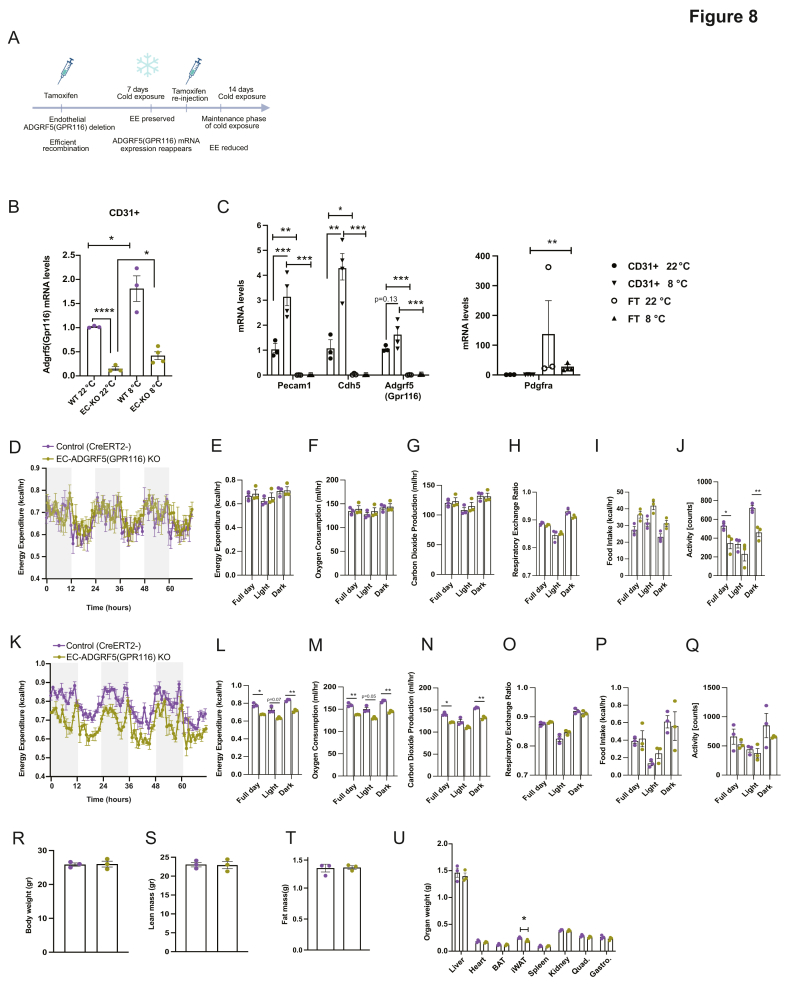
**Endothelial ADGRF5(GPR116) is required to sustain thermogenesis during prolonged cold exposure.** (A) Experimental schematic of prolonged cold exposure in tamoxifen-inducible EC-ADGRF5(GPR116)KO mice. Tamoxifen was administered prior to cold exposure to induce endothelial recombination, followed by a second tamoxifen injection after 7 days, 8 °C, to ensure continued endothelial ADGRF5(GPR116) deletion during the maintenance phase of cold adaptation. (B) qPCR analysis of *Adgrf5(Gpr116)* mRNA levels in CD31^+^ endothelial cells isolated from BAT of WT and endothelial inducible KO mice at 22 °C and after 7 days, 8 °C. (C) qPCR analysis of endothelial markers (*Pecam1*, *Cdh5, Gpr116*) and stromal marker (*Pdgfra*) in CD31^+^ endothelial fractions and flow-through (FT) fractions, demonstrating endothelial specificity of *Adgrf5(Gpr116)* expression and recombination. Data points in (B,C) represent individual mice. (D) Time-resolved energy expenditure during the first week (7 days), 8 °C. (E–J) Metabolic cage analyses, for the last 3 days of week 1 (7 days), 8 °C. (K) Time-resolved energy expenditure during the second week (14 day), at 8 °C, following tamoxifen re-injection. (L–Q) Metabolic cage analyses, for the last 3 days of week 2 (14 days), 8 °C. (R) Body weight, (S) lean mass and (T) fat mass, (U) organ weights of control and EC-Adgrf5(Gpr116) KO mice at the end 14 days, at 8 °C protocol. Mice were females, n = 3 per group, 10–12 weeks old. All mice were single-housed. Grey shaded areas indicate the dark phase. Statistical analyses for indirect calorimetry data were performed using CalR, with group differences assessed using general linear models, with or without ANCOVA, where appropriate to account for body weight or lean mass differences, as implemented in the CalR platform. Body composition parameters were analyzed using unpaired two-tailed Student's t tests. Data are presented as mean ± SEM. ∗p < 0.05, ∗∗p < 0.01, ∗∗∗p < 0.001, otherwise, differences are not significant (ns).

To confirm the endothelial origin of the thermogenic defects observed in the global ADGRF5(GPR116) deletion and, importantly, to temporally define the requirement for endothelial ADGRF5(GPR116) during cold adaptation, we performed prolonged cold exposure experiments in inducible Cdh5-CreERT2; Adgrf5(Gpr116)fl/fl mice (EC- ADGRF5(GPR116)KO) (Figure 8A). Prior to cold exposure, qPCR analysis of isolated endothelial cells confirmed efficient and near-complete deletion of ADGRF5(GPR116), indicating robust recombination following tamoxifen administration (Fig. 8B).

During the first week of cold exposure, energy expenditure, VO_2_, CO_2_, RER, food intake were comparable between WT and inducible KO animals (Figure 8D–I). We only noticed lower activity levels for the inducible KO animals compared to WT (Fig. 8J). qPCR analysis after 7 days, at 8 °C revealed partial reappearance of *Adgrf5(Gpr116)* expression in endothelial cells, indicating partial recovery of receptor expression during this early phase of cold (Fig. 8B). Consistently, analysis of CD31 bead–isolated endothelial cells and flow-through fractions confirmed endothelial specificity of *Adgrf5(Gpr116)* expression and its induction by cold, in parallel with endothelial markers such as *Pecam1* and *Cdh5* (Fig. 8C). This observation is consistent with the absence of a phenotype in heterozygous animals in earlier experiments (Suppl. Fig. 3D–E), indicating that full and sustained endothelial deletion of ADGRF5(GPR116) is required to elicit a thermogenic defect.

To maintain endothelial ADGRF5(GPR116) deletion during the later phase of cold exposure, mice were re-dosed with tamoxifen at the end of 7 days (Fig. 8A). Under these conditions, inducible KO animals exhibited a significant reduction in energy expenditure during week 2 of cold exposure (Fig. 8K–P), phenocopying the impaired maintenance of thermogenesis observed in the constitutive knockout. This effect occurred without differences in locomotor activity (Fig. 8Q) and was observed in both male and female mice, indicating a sex-independent endothelial requirement for ADGRF5(GPR116) in sustaining cold-induced energy expenditure.

We observed no differences in body weight or body composition between genotypes (Fig. 8R–T). To exclude secondary systemic contributions to the thermogenic phenotype, we assessed organ weights and tissue morphology. Heart and spleen weights were comparable between genotypes (Fig. 8U), indicating that the increased heart and spleen mass observed in the global ADGRF5(GPR116)KO does not account for the reduction in energy expenditure in the inducible endothelial model. iWAT weight was modestly reduced in EC- ADGRF5(GPR116)KO mice compared to controls (Fig. 8U); however H&E staining did not reveal overt histological abnormalities in BAT or iWAT (Suppl. Fig. 12A) and UCP1 tended to be lower in iWAT of EC- ADGRF5(GPR116)KO compared to control mice (Suppl. Fig. 12B–C). These findings indicate that the thermogenic defect observed in the inducible model is not driven by gross adipose tissue loss or secondary organ pathology, but rather reflects a functional impairment emerging under sustained endothelial ADGRF5(GPR116) deletion during prolonged cold exposure.

In contrast to the constitutive knockout, FITC–dextran permeability was unchanged in EC- ADGRF5(GPR116)KO mice (Suppl. Fig. 12D). CD31 immunostaining and quantification revealed no differences in vascular abundance at the end of the cold exposure protocol (Suppl. Fig. 12E), and Sirius Red/Fast Green staining (Suppl. Fig. 12F) and hydroxyproline quantification (Suppl. Fig. 12G) showed no evidence of fibrotic remodeling in EC-ADGRF5(GPR116)KO mice. Together, these data demonstrate that endothelial ADGRF5(GPR116) is dispensable for the acute initiation of the cold response, but is required to sustain adaptive thermogenesis during prolonged cold exposure.

## 4 Discussion

In this study, we identify the adhesion GPCR, ADGRF5(GPR116) as a critical endothelial regulator required for the maintenance—but not the initiation—of thermogenic adaptation in adipose tissue. Using integrated single-nucleus transcriptomic profiling of mouse and human brown adipose tissue, we show that ADGRF5(GPR116) is highly enriched in vascular endothelial cells and dynamically regulated by cold exposure. Functional analyses demonstrate that loss of ADGRF5(GPR116) does not impair acute cold-induced activation of thermogenesis, but instead results in a progressive failure to sustain energy expenditure and body temperature during prolonged cold exposure. Importantly, inducible endothelial-specific deletion of ADGRF5(GPR116) phenocopies this defect selectively during the maintenance phase of cold adaptation, establishing an endothelial-intrinsic requirement for ADGRF5(GPR116) in sustaining thermogenic function. Together, these findings position endothelial ADGRF5(GPR116) as a transcriptional and signaling checkpoint that preserves the cold-remodeled vascular niche necessary for long-term thermogenic competence.

These findings extend current models of thermogenic regulation by identifying the vascular endothelium as an active determinant of thermogenic persistence, rather than a passive conduit supporting adipocyte activation. Cold exposure is known to induce robust vascular remodeling in thermogenic adipose tissue, including angiogenesis and endothelial transcriptional reprogramming, highlighting vascular adaptation as an integral component of the thermogenic response [49,50]. Beyond structural remodeling, accumulating evidence demonstrates that endothelial cells actively instruct adipose tissue adaptation through angiocrine signaling, shaping adipocyte recruitment, lipid handling, and metabolic competence [14,48]. Our data advance this framework by demonstrating that endothelial cells must not only initiate vascular remodeling, but also maintain a quiescent yet adaptive endothelial state to stabilize the cold-remodeled tissue environment over time. Loss of ADGRF5(GPR116) disrupts this endothelial homeostasis, leading to maladaptive transcriptional and paracrine reprogramming that compromises the ability of adipocytes to sustain a fully thermogenic identity during prolonged cold exposure. Notably, parallel analyses in iWAT revealed substantially more modest and less coordinated endothelial transcriptional responses to ADGRF5(GPR116) deletion during cold exposure, without emergence of EndMT-like states or robust secretory reprogramming.

Thermogenic remodeling further requires coordinated neurovascular adaptation, with endothelial-derived cues contributing to sympathetic patterning and niche organization within brown fat [15,16]. In this context, our data indicate that endothelial ADGRF5(GPR116) is required to preserve the integrity of this remodeled neurovascular niche during sustained thermogenic demand. Endothelial loss of ADGRF5(GPR116) leads to destabilization of endothelial identity and induction of stress-, matrix-, and transition-associated programs, accompanied by marked changes in angiocrine and paracrine signaling. Thus, durable thermogenesis emerges not simply from repeated adrenergic stimulation of adipocytes, but from continuous endothelial governance of the adipose tissue microenvironment during prolonged cold adaptation.

From a translational perspective, these findings are particularly relevant given the limited durability of brown adipose tissue activity in adult humans. BAT mass and thermogenic capacity decline with aging, prolonged thermoneutral exposure, obesity, and metabolic disease, yet the mechanisms governing this functional involution remain incompletely understood. While most therapeutic strategies have focused on repeated activation of adipocyte-intrinsic thermogenic pathways, comparatively little attention has been given to the vascular and stromal programs required to stabilize a cold-adapted thermogenic tissue state over time. Although, the present study does not directly model aging or thermoneutral housing, the phenotype observed upon endothelial ADGRF5(GPR116) deletion mirrors several functional features associated with BAT involution, including impaired thermogenic efficiency during prolonged cold exposure, failure to sustain a fully oxidative adipocyte state, and preservation of tissue structure despite functional decline. Notably, these defects arise in the absence of impaired sympathetic input, adipocyte loss, overt inflammation, or fibrosis, suggesting that early functional deterioration of thermogenic adipose tissue can occur through disruption of endothelial adaptation rather than terminal tissue degeneration.

More broadly, microvascular dysfunction is increasingly recognized as an early driver of organ decline in chronic disease states. In organs such as the kidney and heart, endothelial injury, altered barrier function, and microvascular remodeling often precede overt parenchymal damage and contribute to progressive loss of tissue function [[51], [52], [53]]. While such mechanisms have not been formally established for BAT involution, these observations provide a conceptual framework in which endothelial resilience and adaptive capacity may represent upstream determinants of long-term thermogenic competence. In this context, our data support a model in which endothelial ADGRF5(GPR116) contributes to the maintenance of a quiescent yet adaptive vascular state required to sustain thermogenic remodeling under prolonged cold demand. Disruption of this endothelial program leads to maladaptive transcriptional and paracrine reprogramming that compromises adipocyte identity and thermogenic efficiency, even in the absence of overt structural pathology.

The molecular mechanisms through which ADGRF5(GPR116) regulates endothelial behavior remain incompletely defined, reflecting the broader context-dependent roles of this receptor across tissues and physiological systems. ADGRF5(GPR116) has been most extensively characterized in epithelial and barrier-associated compartments, where it is essential for pulmonary surfactant homeostasis and barrier integrity, with constitutive deletion leading to progressive lung pathology [21,54]. Beyond the lung, ADGRF5(GPR116) has been implicated in renal filtration and vascular barrier maintenance, suggesting roles at tissue interfaces exposed to mechanical and metabolic stress, although direct endothelial signaling mechanisms remain poorly defined [44,55]. In metabolic tissues, we previously identified ADGRF5(GPR116) as a functional receptor for the hepatokine FNDC4 and demonstrated that FNDC4– ADGRF5(GPR116) signaling in white adipose tissue enhances glucose clearance and improves systemic insulin sensitivity, without affecting energy expenditure or thermoregulation [56]. Consistent with this context specificity, adipocyte-specific deletion of ADGRF5(GPR116) in the present study did not impair cold-induced thermogenesis in vivo, indicating that adipocyte ADGRF5(GPR116) signaling is dispensable for adaptive energy expenditure. Instead, our findings identify endothelial ADGRF5(GPR116) as a critical determinant of sustained thermogenic adaptation, acting through preservation of endothelial identity, vascular integrity, and angiocrine communication during prolonged cold exposure. Together, these observations support a model in which ADGRF5(GPR116) functions not as a universal metabolic switch, but as a context-dependent regulatory receptor whose downstream effects are shaped by cell type, tissue environment, and physiological demand.

Given the strong endothelial enrichment of ADGRF5(GPR116) in BAT, we initially examined its physiological role using a constitutive global knockout model. Several ADGRF5(GPR116) mouse lines have been generated owing to the gene's large size (29 exons), and we selected an exon 2 deletion model that removes the signal peptide and translation start site, producing a non-functional receptor. This model phenocopies the progressive pulmonary abnormalities reported in other endothelial-specific and large-deletion ADGRF5(GPR116) models, confirming that exon 2 deletion is sufficient to abolish receptor function [21]**.** Importantly, pulmonary pathology in these models emerges only after ∼18 weeks of age, whereas all cold-exposure experiments in this study were conducted at 9–12 weeks, well before the onset of lung disease and potenial systemic hypoxia. While the initial discovery of the thermogenic phenotype was made in the global knockout, multiple independent lines of evidence support an endothelial origin of this defect, including the marked endothelial-specific transcriptional reprogramming observed by single-nucleus RNA sequencing, the absence of adipocyte-autonomous thermogenic impairment in vivo, and the phenocopying of impaired thermogenic maintenance in inducible endothelial-specific ADGRF5(GPR116)KO mice. Notably, systemic features observed in the constitutive knockout, such as increased heart and spleen mass, were absent in the inducible endothelial model, further arguing against secondary systemic drivers of the phenotype. Together, these findings indicate that loss of ADGRF5(GPR116) creates a context-specific vulnerability of BAT endothelium during sustained thermogenic demand, rather than a generalized vascular defect.

Endothelial-to-mesenchymal transition (EndMT) has been widely linked to fibro-inflammatory tissue remodeling in diverse pathological contexts, including fibrosis, vascular rarefaction, and chronic inflammation [[57], [58], [59]]**.** In line with this literature, loss of ADGRF5(GPR116) was associated with an increased representation of EndMT-like endothelial states and induction of mesenchymal and extracellular matrix–related transcriptional programs. However, despite these transcriptional and cellular shifts, we did not observe overt fibrosis, collagen accumulation, or immune cell infiltration in BAT of ADGRF5(GPR116)-deficient mice under cold exposure, as assessed by Sirius Red/Fast Green staining (which predominantly labels fibrillar collagens such as types I and III), hydroxyproline content, and macrophage markers. This dissociation suggests that, in this context, EndMT-like reprogramming reflects a partial or early endothelial state transition rather than progression to terminal fibrotic remodeling.

Cold exposure itself induces substantial and reversible extracellular matrix remodeling in BAT, including increases in collagen deposition that accompany thermogenic tissue expansion and regression [60]. Such physiological ECM remodeling may mask or buffer additional collagen accumulation driven by endothelial dysfunction, particularly when assessed using bulk histological approaches. Notably, unlike metabolic stressors such as high-fat diet, cold exposure has not been associated with pathological BAT fibrosis, consistent with the idea that the cold-adapted thermogenic niche tolerates a high baseline level of ECM turnover. In this setting, the functional consequences of endothelial reprogramming are more likely mediated through altered intercellular communication and angiocrine signaling rather than structural fibrosis or inflammatory infiltration. Indeed, loss of ADGRF5(GPR116) profoundly reshaped endothelial secretory and paracrine programs, enriching for extracellular matrix remodeling cues, neurovascular guidance signals, and niche-patterning ligands that collectively redefine the BAT microenvironment. We propose that this altered endothelial microenvironment, rather than fibrosis per se, destabilizes adipocyte identity and compromises the efficiency of sustained thermogenic adaptation during prolonged cold exposure.

In summary, our unbiased single-nucleus transcriptomic screening of mouse and human brown adipose tissue identifies adhesion GPCRs as a prominent and previously underappreciated GPCR class enriched within the thermogenic adipose niche. Among these, ADGRF5(GPR116) emerged as a highly endothelial-enriched receptor whose expression is dynamically regulated by cold exposure. Through complementary constitutive and inducible genetic models, we demonstrate that endothelial ADGRF5(GPR116) is dispensable for acute thermogenic initiation, but functions as a critical gatekeeper of endothelial adaptation required to sustain thermogenic output during prolonged cold exposure. Loss of ADGRF5(GPR116) destabilizes endothelial adaptive reprogramming, alters angiocrine and paracrine signaling, and reshapes the adipose microenvironment in a manner that ultimately compromises thermogenic efficiency. Together, these findings establish endothelial adhesion GPCR signaling as a key regulatory axis in brown fat remodeling and function and provide a conceptual framework for targeting vascular–endothelial pathways to preserve or restore thermogenic capacity under chronic metabolic stress.

## Authors’ contributions

RE.M and V.K, designed and performed experiments, analyzed data, and prepared the manuscript figures. R.K, L.S, R.L, S.G, MY.J, S.Hi, M.H, E,K, AK.J and AT.K, provided material, and performed experiments and provided input that improved the manuscript during preparation. K.D and F.M provided conceptual input and experimental advice, J.H, M.K, A.P, J.Ha, D.W, S.H provided strategic input, critically reviewed the manuscript, supervised students. A.G conceived the study, supervised the project development, analyzed the data, and wrote the manuscript. All authors read and approved the manuscript.

## Funding

This work was supported by funding acquired by the Deutscher Akademischer Austauschdienst (DAAD) and by the Helmholtz Association - Initiative and Networking Fund (IVF) as part of the International Helmholtz Research School for Diabetes to AK.J and A.G This work was supported by the Deutsche Forschungsgemeinschaft (DFG) grant no. 450149205-TRR333/1 (BAT Energy) to K.D, F.M, S.G, J.H, M.K, A.P, J.Ha, D.W. This work was supported by the Deutsche Forschungsgemeinschaft (DFG) grant no. 450149205-TRR333/1 (BAT Energy) to S.H and A.G (Project P03).

## Declaration of competing interest

The authors declare that they have no known competing financial interests or personal relationships that could have appeared to influence the work reported in this paper.

## Appendix A Supplementary data

Multimedia component 1 Multimedia component 1

Multimedia component 10**Supplementary Figure 9, related to**Figure 6**: Assessment of endothelial proliferation and immune cell infiltration in BAT.** (A) Representative immunofluorescence images of BAT sections of WT mice, exposed to 8 °C for 14 days, stained for CD31 (endothelial cells) and Ki67 (proliferation marker), showing minimal endothelial proliferation under the indicated conditions. (B) Representative immunofluorescence image of BAT section stained for Cd68 (macrophages) and Hoechst, showing low immune cell infiltration in BAT and 14days at 8 °C. (C) Positive control tissue stained for Cd68 and Hoechst, demonstrating antibody specificity and robust detection of macrophages. Nuclei were counterstained with Hoechst. Macrophages in the subcapsular sinus and cortex of an inguinal lymph node were used as positive control tissue.

Multimedia component 11**Supplementary Figure 10, related to**Figure 7**: Integrated single-nucleus transcriptomic map of adipocyte and vascular cell subclusters in brown adipose tissue.** UMAP representation of the integrated single-nucleus RNA-seq dataset comprising adipocyte, endothelial, mural, stromal, and immune cell populations from mouse BAT. Distinct adipocyte subclusters and vascular subclusters (including are shown and annotated based on established marker gene expression. This integrated visualization highlights the transcriptional proximity and continuum between adipocyte and vascular compartments, providing a reference framework for interpreting cell–cell interactions and niche remodeling described in the main figures.

Multimedia component 12 **Supplementary Figure 11, related to Figure7: Extended NicheNet analysis of endothelial–adipocyte communication under cold exposure and upon ADGRF5(GPR116) deletion.** (A) Cold-driven endothelial–adipocyte interactions. Heatmap showing predicted adipocyte target genes regulated by endothelial-derived ligands identified by NicheNet analysis under cold exposure (WT 8 °C vs WT 22 °C). Rows represent sender endothelial cell–expressed ligands, and columns represent predicted target genes in adipocytes. Color intensity indicates regulatory potential, with accompanying log fold change (LFC) values reflecting cold-induced gene regulation. (B) Genotype-driven endothelial–adipocyte interactions. Heatmap showing predicted adipocyte target genes regulated by endothelial-derived ligands identified by NicheNet analysis comparing WT and ADGRF5(GPR116)KO conditions after cold exposure (8 °C, 14 days) (KO 8 °C vs WT 8 °C). Rows represent sender endothelial cell–expressed ligands, and columns represent predicted adipocyte target genes. Color intensity indicates regulatory potential. Predicted interactions are based on established ligand–target prior knowledge implemented in the NicheNet framework. Heatmaps display scaled regulatory potential scores. Multimedia component 12

Multimedia component 13**Supplementary Figure 12, related to**Figure 8**: Endothelial-specific ADGRF5(GPR116) deletion does not markedly alter vascular permeability, collagen deposition, or UCP1 staining after cold exposure.** (A) Representative H&E-stained sections of BAT and iWAT from control (Cdh5-CreERT2^-^) and endothelial-specific knockout (EC-ADGRF5(GPR116)KO) mice following cold exposure (8 °C, 14 days). (B) Representative UCP1 immunohistochemistry in BAT and iWAT from control and EC-ADGRF5(GPR116)KO mice after cold exposure (8 °C, 14 days). (C) Quantification of UCP1-positive area normalized to total region of interest (ROI) area in BAT and iWAT, from control and EC-ADGRF5(GPR116)KO mice after cold exposure (8 °C, 14 days). (D) Quantification of FITC–dextran extravasation in BAT, iWAT, gWAT, liver, and lung from control and EC- ADGRF5(GPR116)KO mice following cold exposure. (E) Quantification of CD31-positive area per ROI area in BAT from control and EC-ADGRF5(GPR116)KO mice. (F) Representative Sirius Red/Fast Green–stained BAT sections from control and EC-ADGRF5(GPR116)KO mice following cold exposure. (G) Quantification of hydroxyproline content in BAT and iWAT. All measurements were done after 14 days at 8 °C, at indicated genotypes. For (D), experiments were conducted in male mice; all other panels were generated using female mice. Data are shown as mean ± SEM. Each dot represents one mouse. Statistical significance is indicated where applicable; comparisons without annotation are not significant (ns).

Multimedia component 11 Multimedia component 11

Multimedia component 2 **Supplementary Figure 1, related to**Figure 1: Heatmaps showing expression levels of canonical marker genes across cell clusters in (A) mouse and (B) human BAT. Mouse BAT data were generated in this study. Human BAT data were obtained from a previously published study by *Sun W.* et al. [38]. Multimedia component 2

Multimedia component 14 Multimedia component 14

Extended File 1 Extended File 1

Extended File 2 Extended File 2

Extended File 3 Extended File 3

Extended File 4 Extended File 4

Extended File 5 Extended File 5

Extended File 6 Extended File 6

Extended File 7 Extended File 7

Extended File 8 Extended File 8

Extended File 9 Extended File 9

Multimedia component 3 **Supplementrary Figure 2, related to**Figure 2**: Temperature-related interactions between cell types in BAT.** (A) Heatmaps showing the interaction score between indicating cell types, as senders or receivers. (B) Number of interactions of different categories (cell–cell contact, ECM-receptor, non-protein signaling, and secreted signaling involving endothelial cell (EC) as sender or receiver at indicated ambient temperatures. (C) Bar plots showing the relative information flow for significant communication pathways between all BAT cell types.∗ indicates the aGPCRs pathways. (D) Dot plots presenting significant ligand-aGPCRs receptors predicted to participate in the communication of indicated cell types. The colours of the circles correspond to communication probability. ECs are highlighted in red colour. Multimedia component 3

Multimedia component 4 **Supplementary Figure 3, related to**Figure 3**: Metabolic characterization of ADGRF5(GPR116) KO mice at room temperature (22 °C), including cold challenged female and heterozygous cohorts.** (A-C) *Female mice.* Time-resolved energy expenditure (A) and mean energy expenditure (B) measured by indirect calorimetry in female WT and ADGRF5(GPR116) KO mice housed at 8 °C for 14 days. The last 3 days are shown. (D–E) *Male mice*. Time-resolved energy expenditure (D) and locomotor activity (E) in WT, ADGRF5(GPR116) heterozygous (HET), and ADGRF5(GPR116) KO male mice housed at 8 °C for 14 days. The last 5 days are shown. (F–L) Male mice at room temperature (22 °C). Time-resolved energy expenditure (F) and average metabolic parameters including energy expenditure (G), oxygen consumption (H), carbon dioxide production (I), respiratory exchange ratio (J), food intake (K), and locomotor activity (L), analyzed across full day, light, and dark phases. Grey shaded areas indicate the dark phase. (M) Organ weights from male WT and ADGRF5(GPR116) KO mice at the end of the experimental protocol. Data are shown as mean ± SEM. Each dot represents one mouse. Statistical significance is indicated as ∗ p < 0.05, ∗∗p < 0.01**;** otherwise, differences are not significant (ns). Multimedia component 4

Multimedia component 5 **Supplementary Figure 4, related to**Figure 4, Figure 6**: Single-nucleus transcriptomic atlas of brown and white adipose tissue across genotypes and temperatures.** (A) UMAP representation of integrated single-nucleus RNA-seq data from brown adipose tissue (BAT)**,** showing major adipocyte, endothelial, stromal, immune, and mural cell populations. Cell types were annotated based on established marker gene expression. (B) Heatmap showing scaled expression of canonical marker genes used to define the major BAT cell populations shown in (A). (C) UMAP representations of BAT nuclei from WT and ADGRF5(GPR116) KO mice housed at 22 °C or 8 °C, illustrating the distribution of major cell populations across genotypes and temperature conditions. The number of nuclei analyzed per condition is indicated. (D) Stacked bar plot showing the relative proportions of major BAT cell populations across genotypes and temperatures. (E) UMAP representation of integrated single-nucleus RNA-seq data from inguinal white adipose tissue (iWAT), showing major adipocyte, endothelial, stromal, immune, epithelial, mural, and niche-associated cell populations. (F) Heatmap showing scaled expression of canonical marker genes used to annotate iWAT cell populations shown in (E). (G) UMAP representations of iWAT nuclei from WT and ADGRF5(GPR116) KO mice housed at 22 °C or 8 °C, demonstrating comparable cellular organization across genotypes and thermal conditions. The number of nuclei analyzed per condition is indicated. (H) Stacked bar plot showing the relative proportions of major iWAT cell populations across genotypes and temperatures. Multimedia component 5

Multimedia component 6**Supplementary Figure 5, related to**Figure 4: **Adipose tissue morphology and adipocyte size distribution in ADGRF5(GPR116) KO mice. (**A) Representative hematoxylin and eosin (H&E)–stained sections of BAT, iWAT, and gWAT from WT and ADGRF5(GPR116) KO mice housed at 22 °C or 8 °C for 14days. Low- and high-magnification images are shown. (B) Organ weights from WT and ADGRF5(GPR116) KO mice at the end of the experimental protocol (8 °C for 14 days). (C) Quantification of lipid content in BAT from WT and ADGRF5(GPR116) KO mice under 22 °C and 8 °C. (D–E) iWAT adipocyte morphology. Mean adipocyte area, at 22 °C and 8 °C (14 days) (D) and adipocyte size distribution, at the end of the study, 8 °C for 14 days (E) in iWAT from WT and ADGRF5(GPR116) KO mice. (F–G) gWAT adipocyte morphology. Mean adipocyte area at 22 °C and 8 °C (14 days) (F) and adipocyte size distribution, at the end of the study, 8 °C for 14 days (G) in gWAT from WT and ADGRF5(GPR116) KO mice. (H) Representative brightfield images of SVF-derived adipocytes differentiated from BAT (left) and iWAT (right) of WT, ADGRF5(GPR116) heterozygous (HET), and KO mice, shown separately for male and female mice. Data are shown as mean ± SEM. Each dot represents one mouse. Statistical significance, calculated with unpaired two-tailed Student's t-test is indicated as ∗ p < 0.05, ∗∗p < 0.01; otherwise, differences are not significant (ns).

Multimedia component 7**Supplementary Figure 6, related to**Figure 5**: Adipocyte-specific deletion of Adgrf5(Gpr116) does not alter basal metabolic parameters.** (A–E) Indirect calorimetry parameters in WT and global ADGRF5(GPR116)KO mice housed at room temperature (22 °C) and injected once per day with CL-316,243, 2 mg/kg for 7 days. (F–J) Indirect calorimetry and body composition analysis: body weight (K), lean mass (L), and fat mass (M), in Ucp1-Cre^-^ADGRF5(GPR116)f/f (control) and Ucp1-Cre^+^ADGRF5(GPR116)f/f mice. (N–R) Indirect calorimetry and body weight (S), lean mass (T), and fat mass (U), in Adipoq-Cre^-^ADGRF5(GPR116)f/f (control) and Adipoq-Cre^+^ADGRF5(GPR116)f/f mice. Data are shown as mean ± SEM. Statistical analyses were performed using unpaired two-tailed Student's t test, Statistical significance is indicated as ∗ p < 0.05, ∗∗p < 0.01, otherwise, differences are not significant.

Multimedia component 8**Supplementary Figure 7: Neuroendocrine and systemic circulation parameters in ADGRF5(GPR116)KO mice.** (A) Plasma norepinephrine (NE) levels measured by HPLC in WT and ADGRF5(GPR116)KO mice housed at 22 °C or after 14 days of cold exposure at 8 °C. (B) Tissue norepinephrine content measured by HPLC in BAT and iWAT from WT and ADGRF5(GPR116)KO mice, following 14 days at 8 °C. (C) Western blot analysis of tyrosine hydroxylase (TH) in BAT total lysates from WT and ADGRF5(GPR116)KO mice at 22 °C and 8 °C. VCP was used as a loading control. (D) Serum triiodothyronine (T3) levels in WT and ADGRF5(GPR116)KO mice at 22 °C and after 14 days at 8 °C. (E–H) Serum metabolite measurements following 14 days at 8 °C, including non-esterified fatty acids (NEFA) (E), triglycerides (TG) (F), cholesterol (G), and lactate (H). Mice were male C57BL6N, 10–12 weeks old. Data are shown as mean ± SEM, with each dot representing one mouse. Statistical significance is indicated as ∗ p < 0.05, ∗∗p < 0.01; otherwise, differences are not significant (ns). Statistical analyses were performed using unpaired two-tailed Student's t test.

Multimedia component 9**Supplementary Figure 8, related to**Figure 6**: Annotation and marker gene expression of vascular cell populations in BAT and iWAT.** (A) UMAP representation of integrated single-nucleus RNA-seq data from BAT vascular cells, showing the full set of annotated clusters. Cluster identities are indicated based on established marker gene expression. (B) Heatmap showing scaled expression of representative marker genes across the annotated BAT vascular clusters, supporting cluster identity assignment shown in (A). (C) UMAP representation of integrated single-nucleus RNA-seq data from iWAT vascular cells. (D) Heatmap showing scaled expression of representative marker genes across iWAT vascular clusters, confirming cell-type annotations shown in (C).

## Footnotes

Appendix A Supplementary data to this article can be found online at https://doi.org/10.1016/j.molmet.2026.102346.
