# Supplementary material for: Endothelial ADGRF5(GPR116) governs vascular adaptation required for sustained thermogenic remodeling of brown adipose tissue
Source: Mol Metab. 2026 Mar 6;107:102346. doi: 10.1016/j.molmet.2026.102346 (PMC13053757; doi:10.1016/j.molmet.2026.102346)
Supplement: Multimedia component 1 [file mmc1.docx]

**Supplementary Results**

**S 3.4 Annotation of adipocyte subclusters in mouse BAT and iWAT**

Subsetting and reclustering the adipocyte populations revealed eleven transcriptionally distinct adipocyte subclusters in BAT and seven in iWAT (Fig. 4A,4B). To annotate these subclusters, we integrated their most representative marker genes, and a curated scoring framework capturing key functional categories relevant to thermogenic remodeling. In BAT, we identified **oxidative/mitophagy-enriched brown adipocytes** (Cluster 1), characterized by elevated expression of *Bnip3 (1, 2)*, *Zbtb16 (3)*, *Pla2g7 (4)*, *Ugp2*, and *Ehhadh*, reflecting enhanced mitochondrial turnover and oxidative metabolism. **Early cold-response adipocytes** (Cluster 2) were enriched for immediate early cold-inducible genes such as *Nr4a1 (5)*, *Btc (6)*, and *Sik1 (7, 8)*. A distinct group of **UCP1-high but adrenergic-attenuated (Adrb3 low) neuro-vascular remodeling adipocytes** (Cluster 3) expressed *Bmp8b (9)*, *Elovl3 (10)*, *Slc2a5 (11)*, *Sorcs 2(12)*, and *Prune2 (13)*. We further identified **metabolic-stress/detoxifying adipocytes** (Cluster 4), enriched for *Cyp2b10 (14, 15)*, *Slc7a11 (16)*, *Notch4*, *Hcn2*, and *Sqle*, as well as a population of **basal/housekeeping brown adipocytes** (Cluster 5), marked by *Nat8 l(17)*, *Cirbp (18, 19)*, *Pet100 (20)*, *Lctl*, and *Rcc2*, indicative of a quiescent brown state with low thermogenic activation. Another distinct cluster represented **vascular–immune interacting adipocytes** (Cluster 6), expressing *Igfbp7*, *Igf1 (21, 22)*, *Svep1 (Polydom) (23, 24)* and *Il15 (25, 26)*, consistent with active paracrine crosstalk with endothelial and immune cells (Fig. 4A, 4C).

Two transcriptionally distinct myogenic-like adipocyte populations were also present: **myogenic-contractile adipocytes I (myofibrillar dominant)** (Cluster 7) enriched for *Myh7*, *Tnnt1*, *Myl2*, *Myh4*, *Tnnc2*, *Ckm*, and *Mylpf* and **myogenic-contractile adipocytes II (adipocyte-comitted)** (Cluster 8)**,** exhibiting higher levels of the myofribrillar dominant markers and myogenic program score, while having low UCP1 score (Fig. 4E). Consistent with this annotation, both clusters showed the highest scores for the **futility module**, indicating robust engagement of ATP-consuming sarcomeric and cytoskeletal programs (Fig. 4E). We additionally identified an **interferon-responsive adipocyte cluster (Cluster 9)**, marked by Rsad2(Viperin) (27), Oasl1, Ifit1, Gbp2, and Mx2, reflecting activation of type-I interferon signaling and cellular stress pathways rather than a distinct thermogenic lineage. There was a clear cluster of **white-like lipogenic adipocytes** (Cluster 10), characterized by *Aldh1a1 (28)*, *Retn*, *Nnat (29)*, and *Gsta3 (30)*. Finally, a population of **endothelial-origin adipocytes** (Cluster 11) emerged, defined by the co-expression of canonical adipocyte identity markers (*Cebpa*, *Fabp4, Pparg*) and endothelial signature genes (*Flt1*, *Eng*, *Erg*, *Cxcl12 (31)*), suggesting endothelial-to-adipocyte identity influence or lineage drift (Fig. 4C). Notably, low-level endothelial marker expression were observed in the contractile/myogenic adipocyte clusters may reflect partial fate plasticity or local transcriptional convergence with adjacent vascular cells (Fig.4C).

The curated module-scoring analysis independently validated these identities by revealing selective enrichment of *Ucp1*, *Adrb3*, futile cycles, oxidative/mitophagy, early cold-response, neuro-vascular remodeling, metabolic-stress, vascular–immune interaction, white-like lipogenic, and endothelial identity modules across the corresponding clusters (Fig. 4E, Suppl. Table 4).

Reclustering of iWAT adipocytes resolved seven transcriptionally distinct populations that together captured the spectrum of cold-induced remodeling states (Fig. 4B). Two subtypes represented bona fide thermogenic programs. One consisted of **classical oxidative beige adipocytes** (Cluster 1), defined by strong expression of thermogenic and mitochondrial genes including *Elovl3*, *Hadhb*, *Dio2*, *Cidea*, *Cox7a1*, and *Ppargc1b*, together with detectable *Ucp1* and moderate expression of substrate-metabolism genes (Gpd1, Gpd2, Fasn, Acaca), consistent with canonical oxidative beige identity. A second thermogenic state comprised **futile-cycle** and **myogenic pseudo-beige adipocytes** (Cluster 2). These cells also expressed *Ucp1* but were dominated by ATP-consuming myogenic and Ca²⁺-cycling programs, including *Myh4*, *Myh7*, *Acta1*, *Acta2*, *Tnnc2*, *Atp2a1*, *Atp2a2*, *Ryr1*, and *Ryr2*, along with lipid and glycerol-phosphate cycle genes (*Gpd1*, *Gpd2*, *Fasn*, *Acaca*). This reflects activation of UCP1-independent thermogenesis via SERCA-mediated Ca²⁺ cycling and futile substrate cycles. In addition, we identified **white-to-beige primed adipocytes** (Cluster3**)** enriched for early beige-competency and nutrient-sensing markers including *Slc7a10* (*Asc-1*), *Slc1a3*, *Cidec* and *Art3* and with low Ucp1 but high Adrb3 in WT mice, positioning them as a population poised for adrenergic-dependent beige recruitment. A distinct **ECM-interacting transitional population** (Cluster 4**)** expressed matrix-associated and profibrotic genes such as *Casq1*, *Fndc1*, *Tnxb*, *Col6a3*, *Igfbp7*, and *Sparc*, consistent with stromal reorganization during cold adaptation. In contrast, **white lipid-storage adipocytes** (Cluster 5**)** retained hallmark white identity markers, *Scd1*, *Plin1*, *Cyp2e1*, *Mgll*, and *Nrip1*, representing the classical energy-storing adipocyte identity that persists in iWAT even under cold exposure. We also identified a population of **stress-responsive adipocytes** (Cluster 6)**,** characterized by metabolic and cellular stress-associated genes such as *Fnip2(32)*, *Cd36*, *Pck1*, and *Prkce*, reflecting adaptive nutrient and stress responses activated during cold challenging. Finally, **cycling and preadipocyte-like adipocytes** (Cluster 7) expressed proliferative and immature markers including *Ccnb1ip1*, *Camk1d*, and *Rpph1*, consistent with adipocyte turnover and regeneration during tissue remodeling (Fig. 4D). These seven identities were supported by module-scoring analysis, which revealed selective enrichment of oxidative/beige, UCP1-independent futile-cycle, myogenic/cytoskeletal-stress, ECM-interaction, white-identity, stress-response, and proliferative signatures across the respective subclusters (Fig. 4F, Suppl. Table 4).

**S 3.6 Annotation of vascular subclusters in mouse BAT and iWAT**

Subsetting and reclustering the vascular populations, including endothelial and mular cells resolved multiple transcriptionally distinct EC populations, spanning the vascular hierarchy. The largest EC population corresponded to **aerocyte-like capillary endothelial cells** (Cluster 1), defined by high expression of Car4 together with core microvascular and barrier genes (Kdr, Emcn, and Robo4), consistent with a capillary subtype specialized for tissue perfusion and oxygen exchange. Arterial ECs were represented by a **canonical arterial cluster** (Cluster 2) enriched for the Notch/arterial gene program (Efnb2, Dll4, Sox17, and Gja5), as well as a closely related **arterial remodeling state** (Cluster 3) (*Vcam1*, *Fn1*, *Jag1*, *Dkk2*) sharing the same core arterial signature, whereas an additional **arterial-like EC cluster** (Cluster 4) exhibited weaker arterial marker expression without a clearly distinct transcriptional program, consistent with an intermediate or transitional arterial EC state.

On the venous side, **venular ECs** (Cluster 5) were defined by Nr2f2, Vwf, and Selp, with strong enrichment of permeability- and activation-associated markers (Plvap, Nt5e, Il33, and Thsd7a), while a second **venular-like cluster** (Cluster 6) expressed the same venular marker axis at lower levels, suggesting a low-identity or intermediate venular EC state. A distinct **metabolic capillary ECs** (Cluster 7) was characterized by selective expression of Mfsd2a, Ptgds, and Ffar4, consistent with a specialized capillary subtype involved in lipid and metabolic exchange, and **lymphatic ECs** (Cluster 8) were unambiguously identified by expression of Prox1, Lyve1, Pdpn, and Flt4. In addition to endothelial populations, reclustering resolved multiple **mural and perivascular cell types**, including **classical pericytes** (Cluster 9; Kcnj8, Abcc9, Rgs5, and Pdgfrb), **metabolically active pericytes** with high mitochondrial and lipid metabolic gene expression (Cluster 10; Rgs5, Scd1, and Fasn), two **vascular smooth muscle cell populations** corresponding to arterial and arteriolar SMCs (Cluster 11 and Cluster 12; Acta2, Tagln, Myh11, and Cnn1), and an **adventitial fibro-immune stromal population** (Cluster 13) marked by Pdgfra, Dcn, Lama2, and Ptprc. Notably, two mesenchymal transition–associated populations were also detected, including a **proliferative EndMT-like state** (Cluster 14) marked by cell cycle genes (Mki67, Top2a, Snai1,Snai2, Zeb1,Vim), EMT-associated transcription factors (Snai1 and Zeb2), and mesenchymal markers (Vim), as well as a **myofibroblast-like mesenchymal population** (Cluster 15) enriched for extracellular matrix and contractile genes (Col1a1, Postn, Acta2, Tagln, and Twist2). Several small clusters exhibited **adipocyte**- (Cluster 16,17) or **myofiber-associated transcriptional signatures (**Cluster 18,19**)** with **low vascular marker expression**; while these may reflect technical contamination, ambient RNA, or rare transitional states, their identity could not be conclusively resolved. Accordingly, these populations were retained in global analyses but were not used as the basis for endothelial subtype–specific mechanistic interpretations.

To determine whether endothelial diversity and ADGRF5(GPR116)-dependent remodeling extend beyond BAT, we performed an analogous high-resolution reclustering of vascular-lineage nuclei from iWAT. This analysis resolved eight transcriptionally distinct populations (clusters 1–8), which together define a compact but structured vascular architecture. The endothelial compartment comprised multiple EC states, including two capillary EC populations distinguished by metabolic versus lipid-handling programs. A **metabolically-enriched capillary EC cluster** (Cluster 1) was characterized by high expression of *Slc1a3*, *Ghr*, *Cidec*, and solute carrier genes, consistent with close coupling to adipocyte substrate exchange, while a second **lipid handling** **capillary EC population** (Cluster 2) selectively expressed *Cd36*, *Mgll*, *Cdh13*, and related lipid-handling genes. In addition, **a venous and post-capillary venule EC cluster** (Cluster 3) was defined by enrichment of *Vwf*, *Thsd7a*, *Ptprb*, and *Entpd1*, consistent with permeability and immune-associated venular functions, and an **arterial/arteriolar EC population** (Cluster 4) expressed regulatory arterial-associated genes including *Sema3g*, *Heg1*, *Smad6*, *Efna5*, and extracellular matrix-related transcripts. Beyond endothelial populations, reclustering resolved **canonical mural cell types**, including Rgs5⁺/Pdgfrb⁺ **pericytes** (Cluster 6) enriched for ion-channel and contractile genes (*Rgs5*, *Pdgfrb*, *Notch3*, *Abcc9*, *Ano1*, *Kcnq5*, *Prkg1*), and a distinct Acta2⁺/Myh11⁺ **vascular smooth muscle cell population** (Cluster 7) corresponding to arterial and arteriolar smooth muscle (*Myh11*, *Acta2*, *Myocd*, *Carmn*, *Mylk*, *Kcnma1*). In addition, **a perivascular adventitial stromal population** (Cluster 8) was identified, characterized by enrichment of ECM-, signaling-, and inflammation-associated genes, indicative of a fibro-inflammatory niche adjacent to vessels (*Rassf2, Fndc1, Egfr, Gas7, Gpc6, Tnxb, Frmd4b, Unc93b1*). Finally, a **Low-complexity cluster** (Cluster 5) exhibited predominant enrichment of mitochondrial and ribosomal transcripts with minimal enrichment of endothelial or mural lineage markers. As this population lacked a clear vascular identity, it was retained for completeness in visualization, but was not interpreted as a discrete endothelial lineage and was excluded from downstream vascular functional analyses. Notably, and in contrast to BAT, no discrete endothelial-to-mesenchymal transition (EndMT) populations were detected in iWAT; instead, fibrotic or matrix-associated transcriptional features were confined to stromal compartments rather than reflecting overt endothelial lineage conversion.

**References Supplementary Results**

1. Choi JW, Jo A, Kim M, Park HS, Chung SS, Kang S, et al. BNIP3 is essential for mitochondrial bioenergetics during adipocyte remodelling in mice. Diabetologia. 2016;59(3):571-81.

2. Song Y, Li D, Su D, Jiang T, Li L, Zhan S, et al. Short-term heat exposure affects thermogenesis and mitophagy in goat brown adipocytes. BMC Genomics. 2025;26(1):272.

3. Plaisier CL, Bennett BJ, He A, Guan B, Lusis AJ, Reue K, et al. Zbtb16 has a role in brown adipocyte bioenergetics. Nutr Diabetes. 2012;2(9):e46.

4. Spadaro O, Youm Y, Shchukina I, Ryu S, Sidorov S, Ravussin A, et al. Caloric restriction in humans reveals immunometabolic regulators of health span. Science. 2022;375(6581):671-7.

5. Cooper E, Spaulding SW. Effects of thyrotropin on the phosphorylation of histones and nonhistone phosphoproteins in micrococcal nuclease-sensitive and resistant thyroid chromatin. Endocrinology. 1983;112(5):1816-22.

6. Ramos CC, Pires J, Gonzalez E, Garcia-Vallicrosa C, Reis CA, Falcon-Perez JM, et al. Extracellular vesicles in tumor-adipose tissue crosstalk: key drivers and therapeutic targets in cancer cachexia. Extracell Vesicles Circ Nucl Acids. 2024;5(3):371-96.

7. Paulo E, Wu D, Wang Y, Zhang Y, Wu Y, Swaney DL, et al. Sympathetic inputs regulate adaptive thermogenesis in brown adipose tissue through cAMP-Salt inducible kinase axis. Sci Rep. 2018;8(1):11001.

8. Shi F, de Fatima Silva F, Liu D, Patel HU, Xu J, Zhang W, et al. Salt-inducible kinase inhibition promotes the adipocyte thermogenic program and adipose tissue browning. Mol Metab. 2023;74:101753.

9. Pellegrinelli V, Peirce VJ, Howard L, Virtue S, Turei D, Senzacqua M, et al. Adipocyte-secreted BMP8b mediates adrenergic-induced remodeling of the neuro-vascular network in adipose tissue. Nat Commun. 2018;9(1):4974.

10. Westerberg R, Mansson JE, Golozoubova V, Shabalina IG, Backlund EC, Tvrdik P, et al. ELOVL3 is an important component for early onset of lipid recruitment in brown adipose tissue. J Biol Chem. 2006;281(8):4958-68.

11. Reinisch I, Klymiuk I, Michenthaler H, Moyschewitz E, Galhuber M, Krstic J, et al. p53 Regulates a miRNA-Fructose Transporter Axis in Brown Adipose Tissue Under Fasting. Front Genet. 2022;13:913030.

12. Subkhangulova A, Malik AR, Hermey G, Popp O, Dittmar G, Rathjen T, et al. SORCS1 and SORCS3 control energy balance and orexigenic peptide production. EMBO Rep. 2018;19(4).

13. Iwama E, Tsuchimoto D, Iyama T, Sakumi K, Nakagawara A, Takayama K, et al. Cancer-related PRUNE2 protein is associated with nucleotides and is highly expressed in mature nerve tissues. J Mol Neurosci. 2011;44(2):103-14.

14. Koga T, Yao PL, Goudarzi M, Murray IA, Balandaram G, Gonzalez FJ, et al. Regulation of Cytochrome P450 2B10 (CYP2B10) Expression in Liver by Peroxisome Proliferator-activated Receptor-beta/delta Modulation of SP1 Promoter Occupancy. J Biol Chem. 2016;291(48):25255-63.

15. Heintz MM, Kumar R, Rutledge MM, Baldwin WS. Cyp2b-null male mice are susceptible to diet-induced obesity and perturbations in lipid homeostasis. J Nutr Biochem. 2019;70:125-37.

16. Yang M, Chen X, Hu X, Li H, Huang H, Fang Y, et al. The NF-kappaB-SLC7A11 axis regulates ferroptosis sensitivity in inflammatory macrophages. Cell Insight. 2025;4(4):100257.

17. Felix JB, Saha PK, de Groot EL, Tan L, Sharp R, Anaya ES, et al. N-acetylaspartate from fat cells regulates postprandial body temperature. Nat Metab. 2025;7(8):1524-35.

18. Gotic I, Omidi S, Fleury-Olela F, Molina N, Naef F, Schibler U. Temperature regulates splicing efficiency of the cold-inducible RNA-binding protein gene Cirbp. Genes Dev. 2016;30(17):2005-17.

19. Logan SM, Storey KB. Cold-inducible RNA-binding protein Cirp, but not Rbm3, may regulate transcript processing and protection in tissues of the hibernating ground squirrel. Cell Stress Chaperones. 2020;25(6):857-68.

20. Vidoni S, Harbour ME, Guerrero-Castillo S, Signes A, Ding S, Fearnley IM, et al. MR-1S Interacts with PET100 and PET117 in Module-Based Assembly of Human Cytochrome c Oxidase. Cell Rep. 2017;18(7):1727-38.

21. Boucher J, Mori MA, Lee KY, Smyth G, Liew CW, Macotela Y, et al. Impaired thermogenesis and adipose tissue development in mice with fat-specific disruption of insulin and IGF-1 signalling. Nat Commun. 2012;3:902.

22. Chang HR, Kim HJ, Xu X, Ferrante AW, Jr. Macrophage and adipocyte IGF1 maintain adipose tissue homeostasis during metabolic stresses. Obesity (Silver Spring). 2016;24(1):172-83.

23. Morooka N, Futaki S, Sato-Nishiuchi R, Nishino M, Totani Y, Shimono C, et al. Polydom Is an Extracellular Matrix Protein Involved in Lymphatic Vessel Remodeling. Circ Res. 2017;120(8):1276-88.

24. Elenbaas JS, Jung IH, Coler-Reilly A, Lee PC, Alisio A, Stitziel NO. The emerging Janus face of SVEP1 in development and disease. Trends Mol Med. 2023;29(11):939-50.

25. Barra NG, Reid S, MacKenzie R, Werstuck G, Trigatti BL, Richards C, et al. Interleukin-15 contributes to the regulation of murine adipose tissue and human adipocytes. Obesity (Silver Spring). 2010;18(8):1601-7.

26. Lee BC, Lee J. Cellular and molecular players in adipose tissue inflammation in the development of obesity-induced insulin resistance. Biochim Biophys Acta. 2014;1842(3):446-62.

27. Eom J, Kim JJ, Yoon SG, Jeong H, Son S, Lee JB, et al. Intrinsic expression of viperin regulates thermogenesis in adipose tissues. Proc Natl Acad Sci U S A. 2019;116(35):17419-28.

28. Kiefer FW, Vernochet C, O'Brien P, Spoerl S, Brown JD, Nallamshetty S, et al. Retinaldehyde dehydrogenase 1 regulates a thermogenic program in white adipose tissue. Nat Med. 2012;18(6):918-25.

29. Gburcik V, Cleasby ME, Timmons JA. Loss of neuronatin promotes "browning" of primary mouse adipocytes while reducing Glut1-mediated glucose disposal. Am J Physiol Endocrinol Metab. 2013;304(8):E885-94.

30. Jowsey IR, Smith SA, Hayes JD. Expression of the murine glutathione S-transferase alpha3 (GSTA3) subunit is markedly induced during adipocyte differentiation: activation of the GSTA3 gene promoter by the pro-adipogenic eicosanoid 15-deoxy-Delta12,14-prostaglandin J2. Biochem Biophys Res Commun. 2003;312(4):1226-35.

31. Lee D, Benvie AM, Steiner BM, Kolba NJ, Ford JG, McCabe SM, et al. Smooth muscle cell-derived Cxcl12 directs macrophage accrual and sympathetic innervation to control thermogenic adipose tissue. Cell Rep. 2024;43(5):114169.

32. Ramirez Reyes JMJ, Cuesta R, Pause A. Folliculin: A Regulator of Transcription Through AMPK and mTOR Signaling Pathways. Front Cell Dev Biol. 2021;9:667311.
