# Supplementary figures and images for: Endothelial ADGRF5(GPR116) governs vascular adaptation required for sustained thermogenic remodeling of brown adipose tissue
Source: Mol Metab. 2026 Mar 6;107:102346. doi: 10.1016/j.molmet.2026.102346 (PMC13053757; doi:10.1016/j.molmet.2026.102346)

A

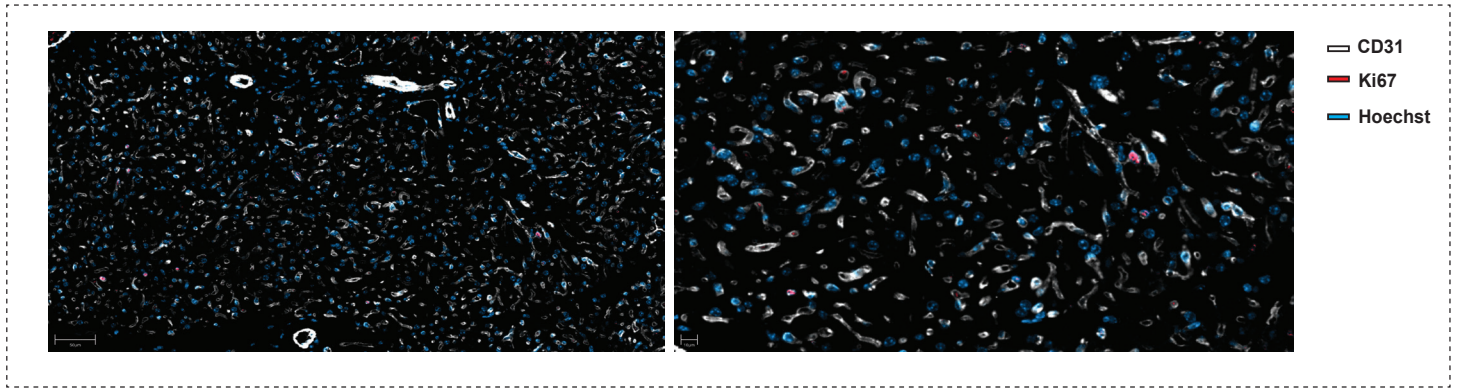

B

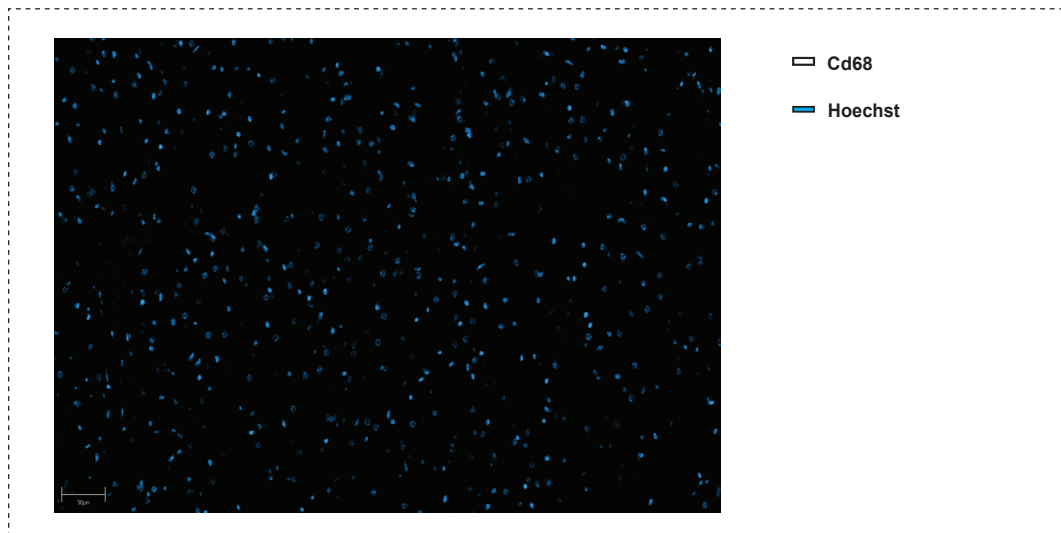

C

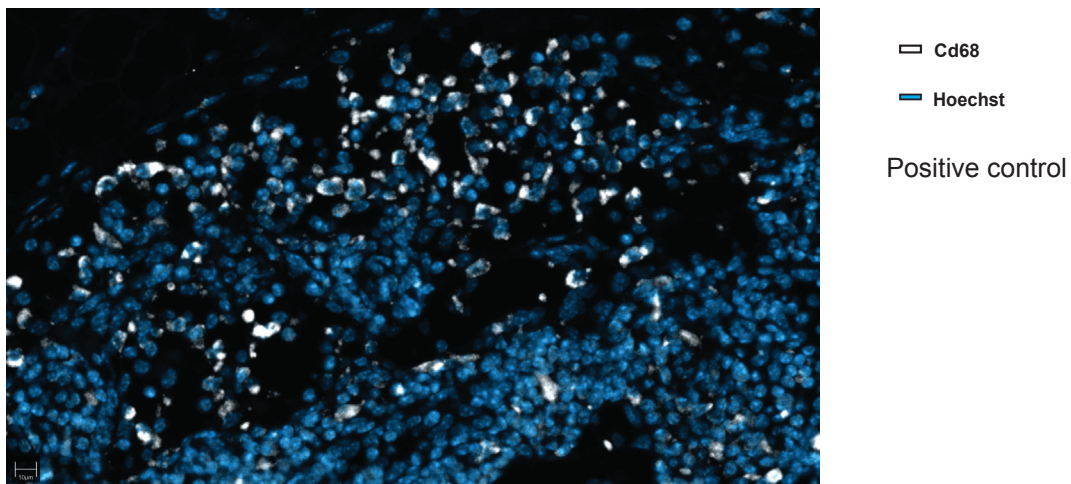

Supplement: Supplementary file 2 — Multimedia component 10Supplementary Figure 9, related toFigure 6: Assessment of endothelial proliferation and immune cell infiltration in BAT. (A) Representative immunofluorescence images of BAT sections of WT mice, exposed to 8 °C for 14 days, stained for CD31 (endothelial cells) and Ki67 (proliferation marker), showing minimal endothelial proliferation under the indicated conditions. (B) Representative immunofluorescence image of BAT section stained for Cd68 (macrophages) and Hoechst, showing low immune cell infiltration in BAT and 14days at 8 °C. (C) Positive control tissue stained for Cd68 and Hoechst, demonstrating antibody specificity and robust detection of macrophages. Nuclei were counterstained with Hoechst. Macrophages in the subcapsular sinus and cortex of an inguinal lymph node were used as positive control tissue. [file mmc10.pdf]

Supplementary Figure 10

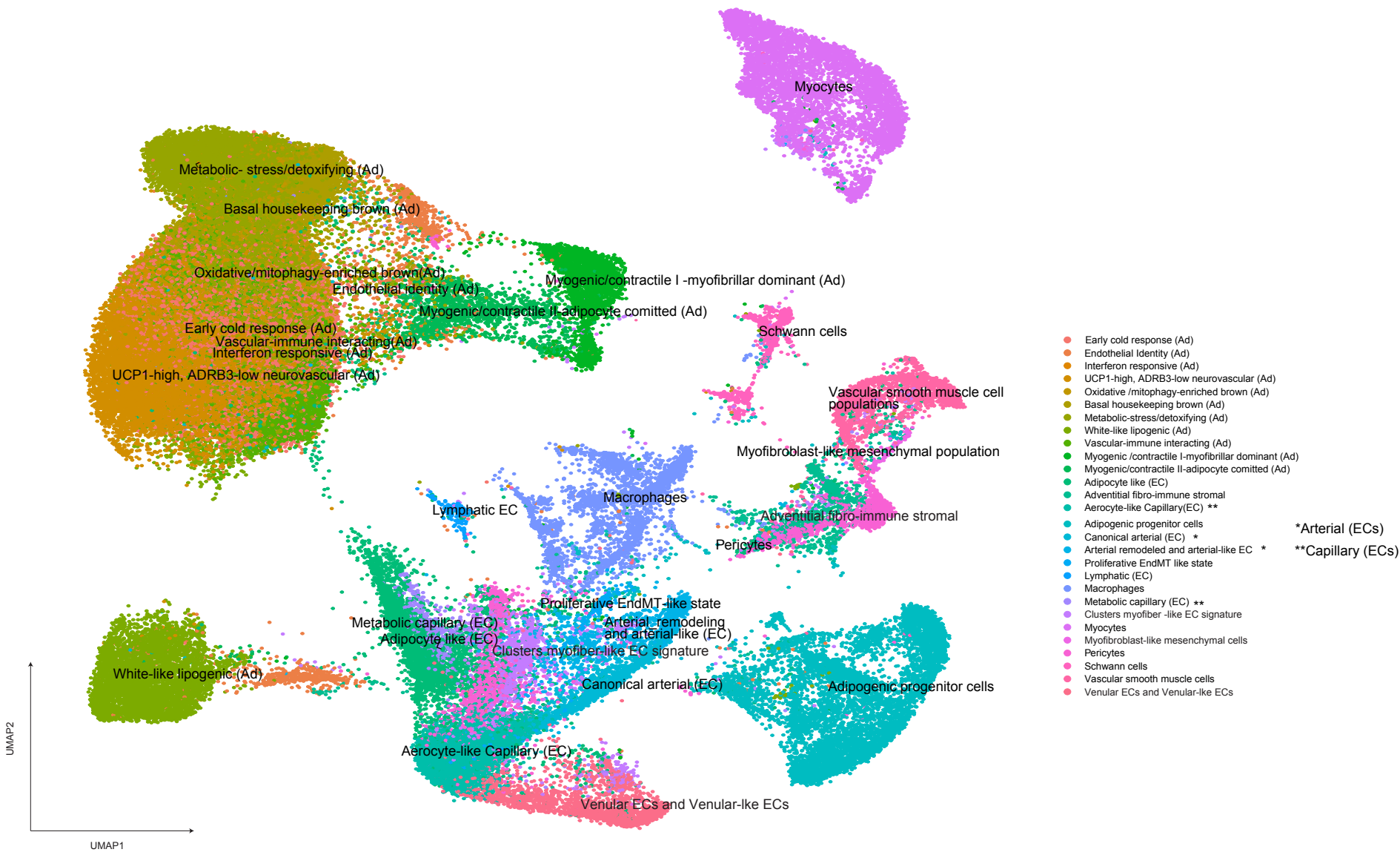

Supplement: Supplementary file 3 — Multimedia component 11Supplementary Figure 10, related toFigure 7: Integrated single-nucleus transcriptomic map of adipocyte and vascular cell subclusters in brown adipose tissue. UMAP representation of the integrated single-nucleus RNA-seq dataset comprising adipocyte, endothelial, mural, stromal, and immune cell populations from mouse BAT. Distinct adipocyte subclusters and vascular subclusters (including are shown and annotated based on established marker gene expression. This integrated visualization highlights the transcriptional proximity and continuum between adipocyte and vascular compartments, providing a reference framework for interpreting cell–cell interactions and niche remodeling described in the main figures. [file mmc11.pdf]

A

## Predicted target genes- Cold driven

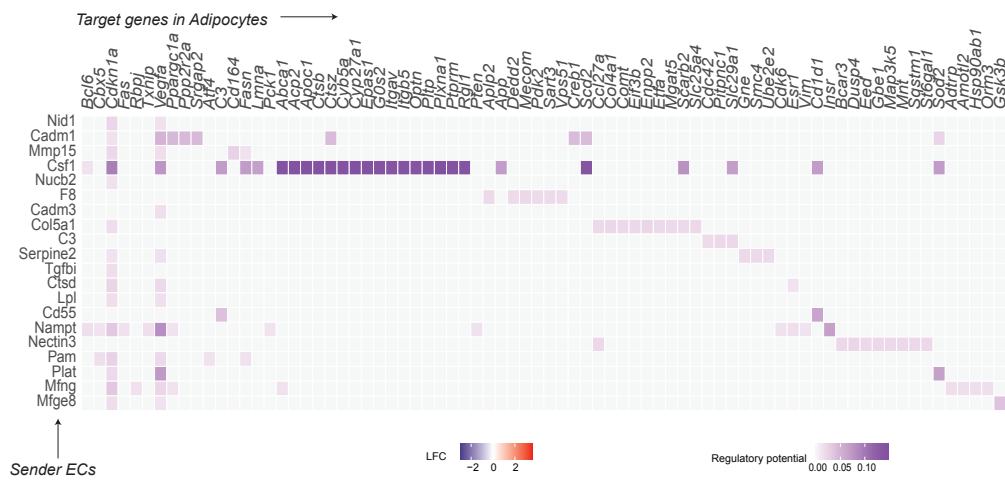

B

## Predicted target genes- Genotype driven

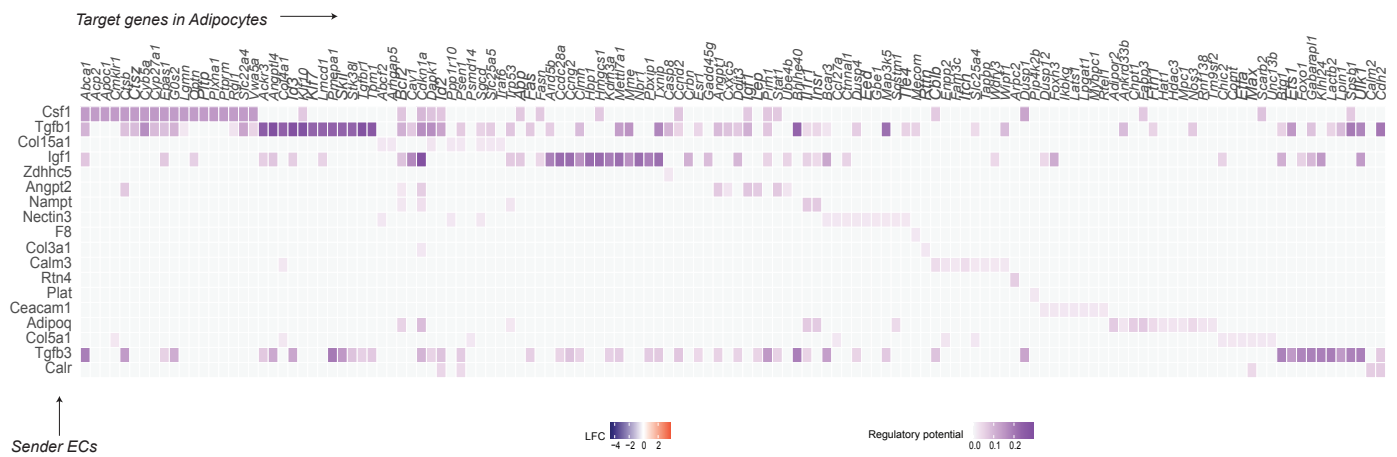

Supplement: Multimedia component 12 — Supplementary Figure 11, related to Figure7: Extended NicheNet analysis of endothelial–adipocyte communication under cold exposure and upon ADGRF5(GPR116) deletion. (A) Cold-driven endothelial–adipocyte interactions. Heatmap showing predicted adipocyte target genes regulated by endothelial-derived ligands identified by NicheNet analysis under cold exposure (WT 8 °C vs WT 22 °C). Rows represent sender endothelial cell–expressed ligands, and columns represent predicted target genes in adipocytes. Color intensity indicates regulatory potential, with accompanying log fold change (LFC) values reflecting cold-induced gene regulation. (B) Genotype-driven endothelial–adipocyte interactions. Heatmap showing predicted adipocyte target genes regulated by endothelial-derived ligands identified by NicheNet analysis comparing WT and ADGRF5(GPR116)KO conditions after cold exposure (8 °C, 14 days) (KO 8 °C vs WT 8 °C). Rows represent sender endothelial cell–expressed ligands, and columns represent predicted adipocyte target genes. Color intensity indicates regulatory potential. Predicted interactions are based on established ligand–target prior knowledge implemented in the NicheNet framework. Heatmaps display scaled regulatory potential scores. [file mmc12.pdf]

**Supplementary Figure 12**

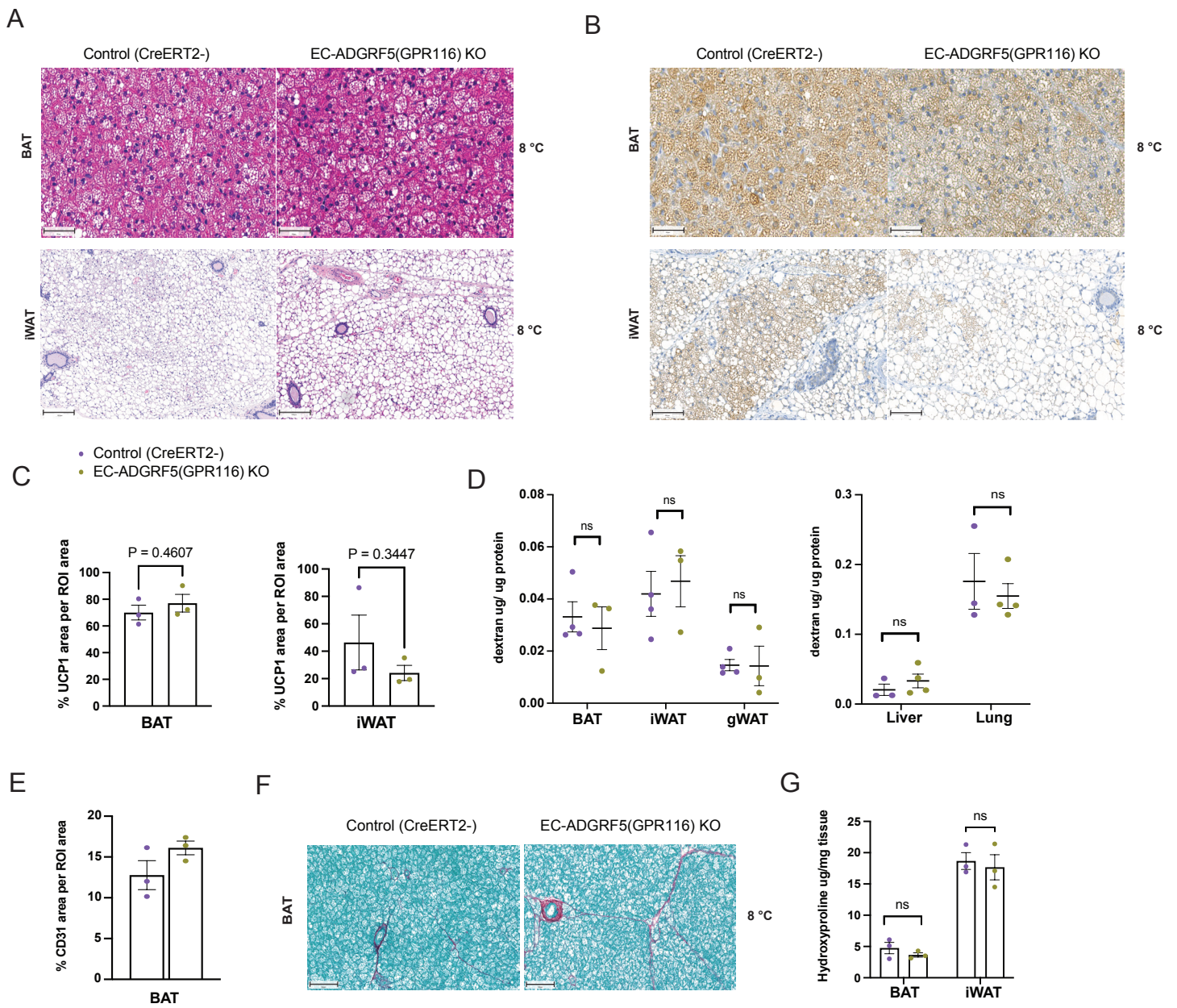

Supplement: Supplementary file 5 — Multimedia component 13Supplementary Figure 12, related toFigure 8: Endothelial-specific ADGRF5(GPR116) deletion does not markedly alter vascular permeability, collagen deposition, or UCP1 staining after cold exposure. (A) Representative H&E-stained sections of BAT and iWAT from control (Cdh5-CreERT2-) and endothelial-specific knockout (EC-ADGRF5(GPR116)KO) mice following cold exposure (8 °C, 14 days). (B) Representative UCP1 immunohistochemistry in BAT and iWAT from control and EC-ADGRF5(GPR116)KO mice after cold exposure (8 °C, 14 days). (C) Quantification of UCP1-positive area normalized to total region of interest (ROI) area in BAT and iWAT, from control and EC-ADGRF5(GPR116)KO mice after cold exposure (8 °C, 14 days). (D) Quantification of FITC–dextran extravasation in BAT, iWAT, gWAT, liver, and lung from control and EC- ADGRF5(GPR116)KO mice following cold exposure. (E) Quantification of CD31-positive area per ROI area in BAT from control and EC-ADGRF5(GPR116)KO mice. (F) Representative Sirius Red/Fast Green–stained BAT sections from control and EC-ADGRF5(GPR116)KO mice following cold exposure. (G) Quantification of hydroxyproline content in BAT and iWAT. All measurements were done after 14 days at 8 °C, at indicated genotypes. For (D), experiments were conducted in male mice; all other panels were generated using female mice. Data are shown as mean ± SEM. Each dot represents one mouse. Statistical significance is indicated where applicable; comparisons without annotation are not significant (ns). [file mmc13.pdf]

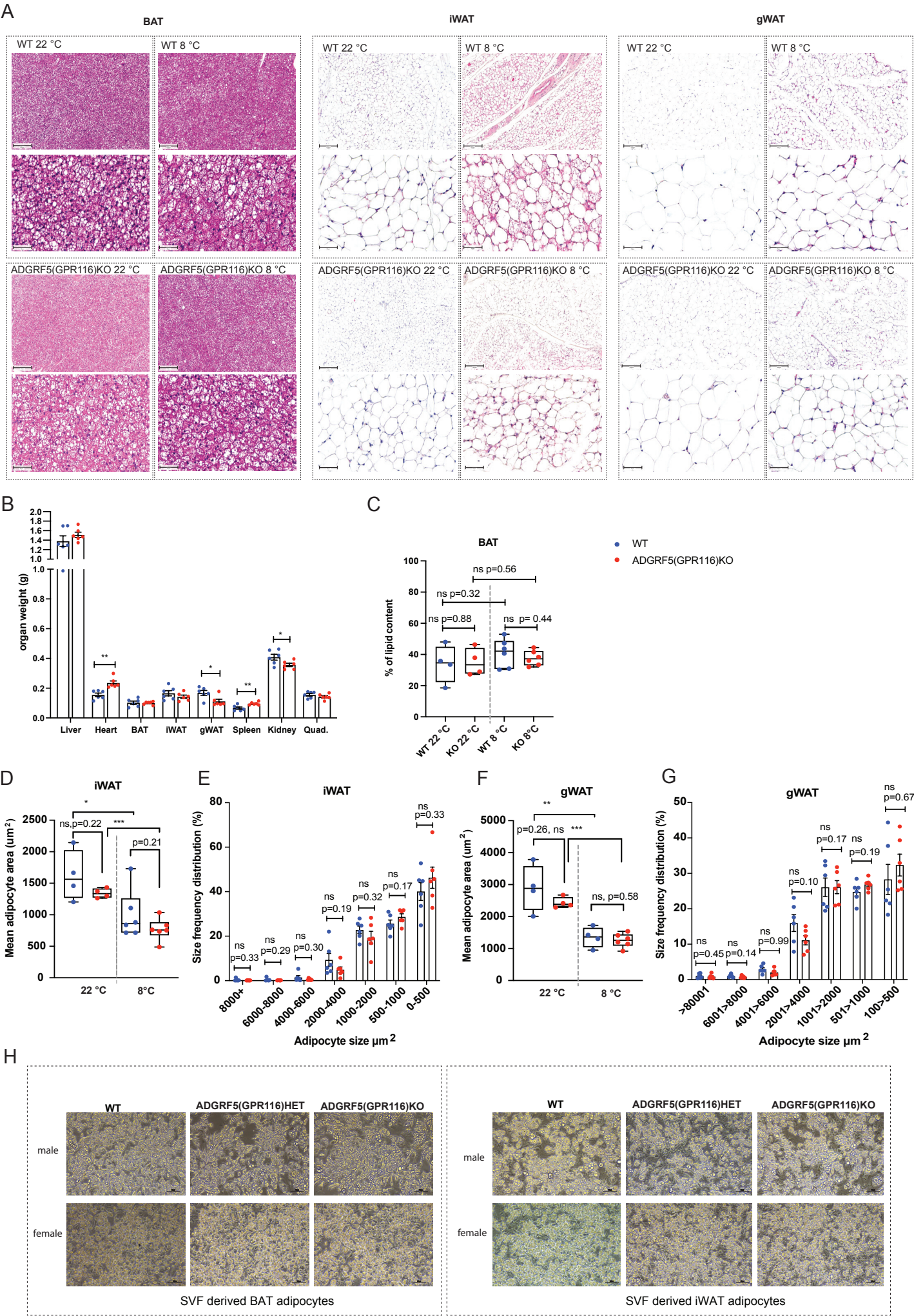

Supplement: Multimedia component 6 [file mmc6.pdf]

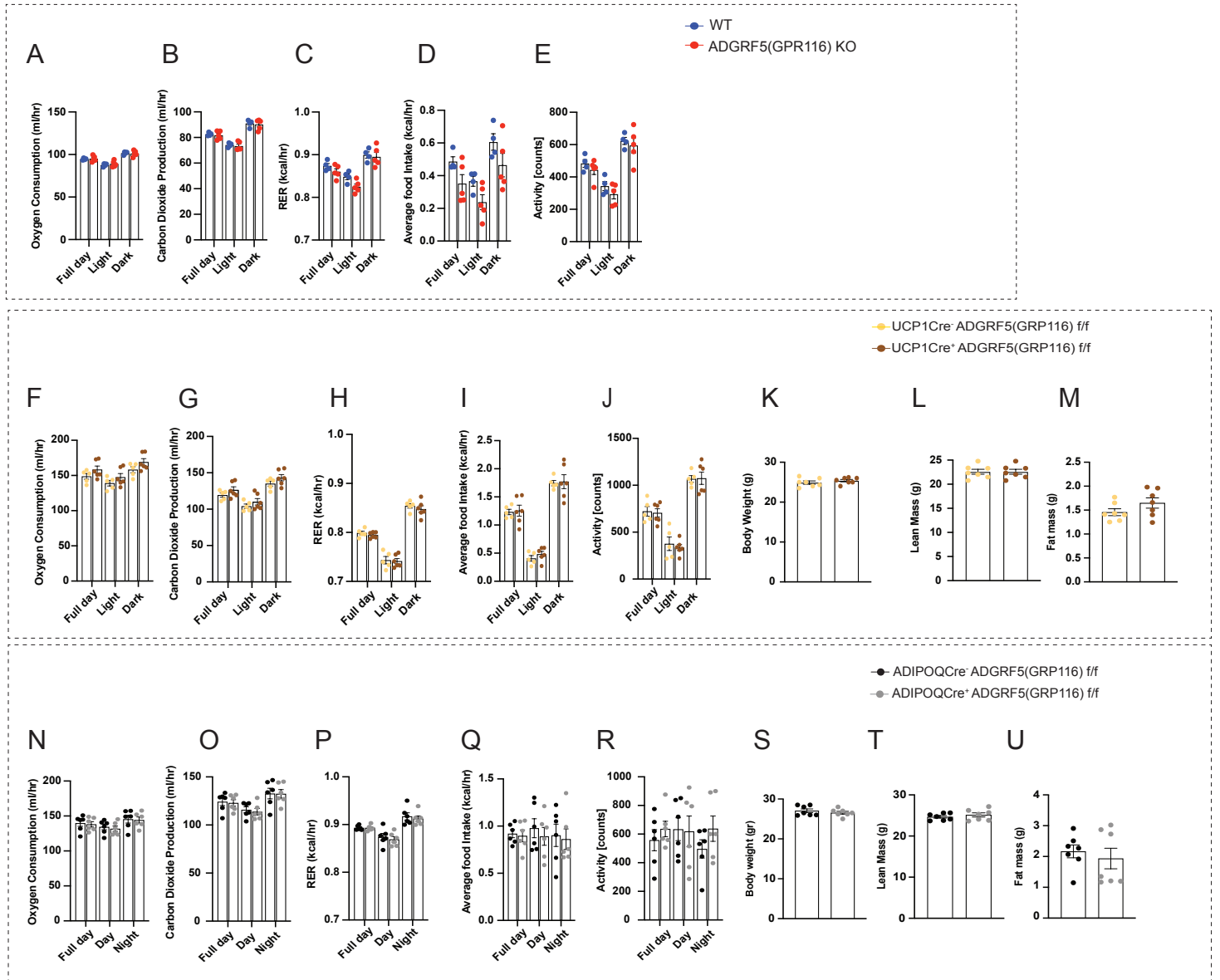

Supplement: Multimedia component 7 [file mmc7.pdf]

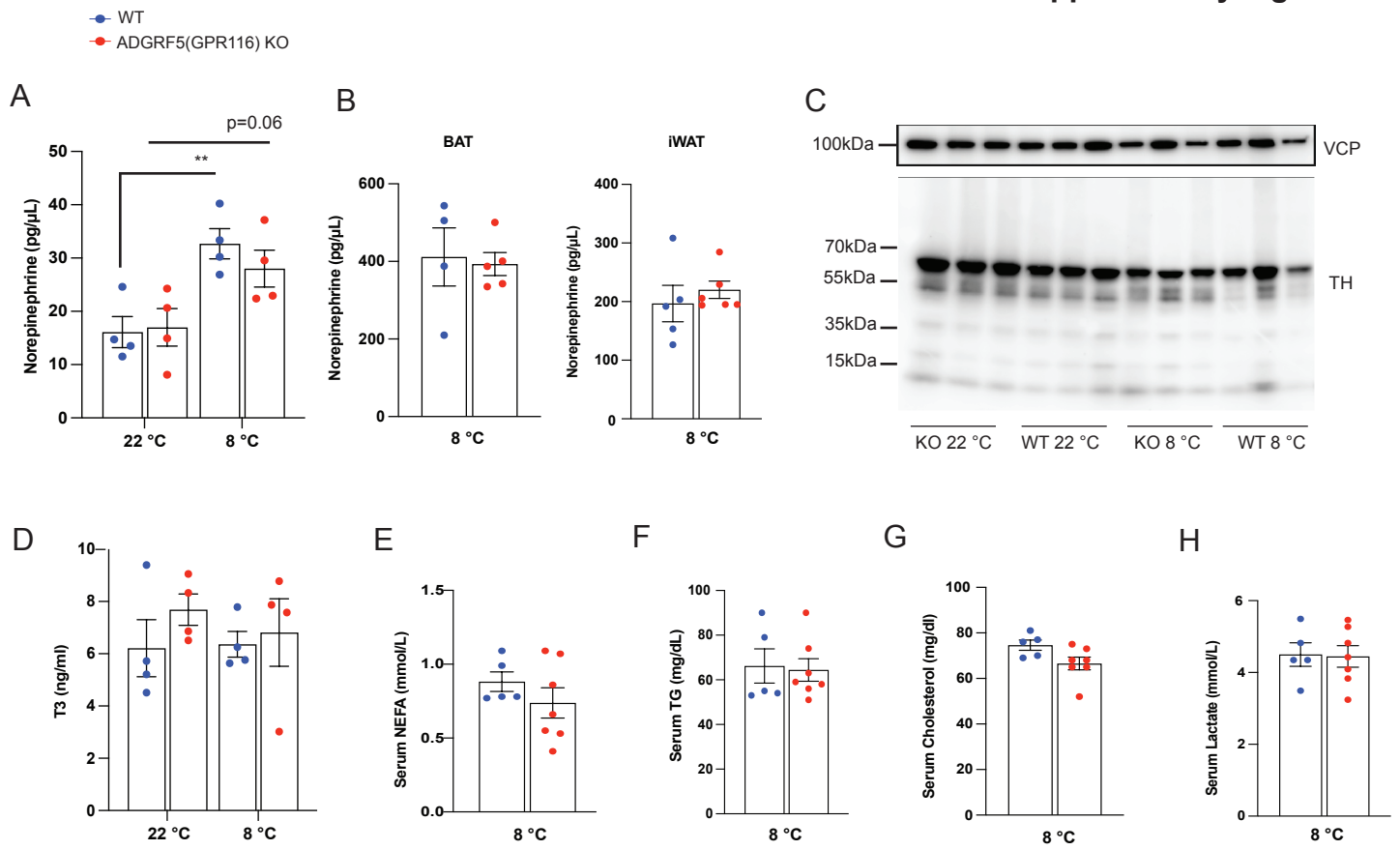

Supplement: Multimedia component 8 [file mmc8.pdf]

A

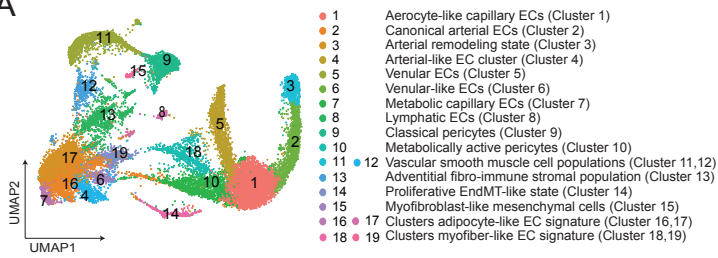

B

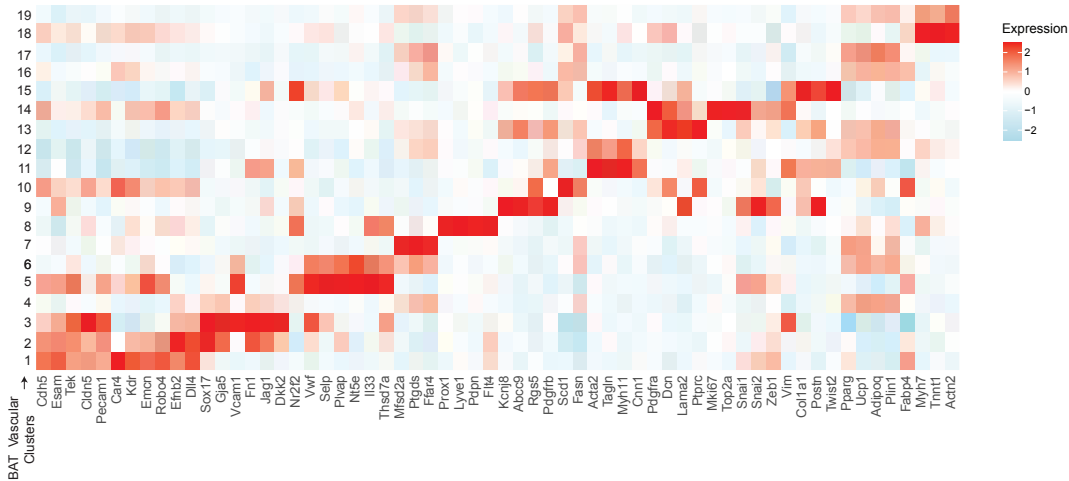

C

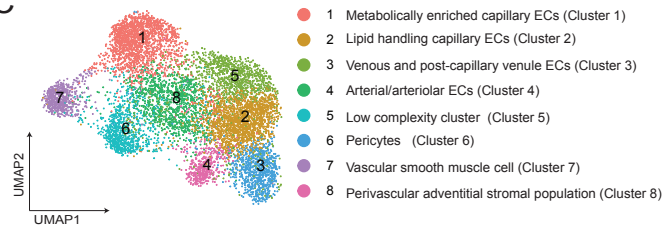

D

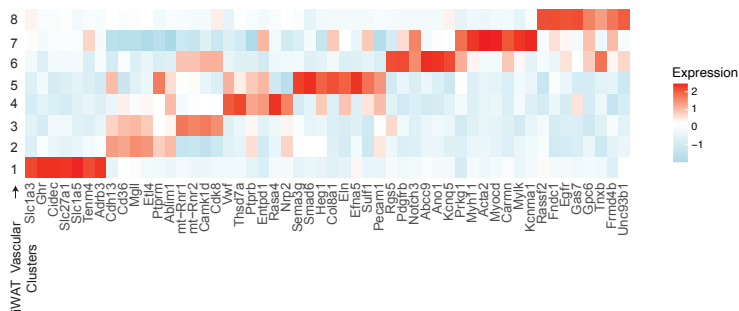

Supplement: Multimedia component 9 [file mmc9.pdf]

Supplementary Figure 1

A

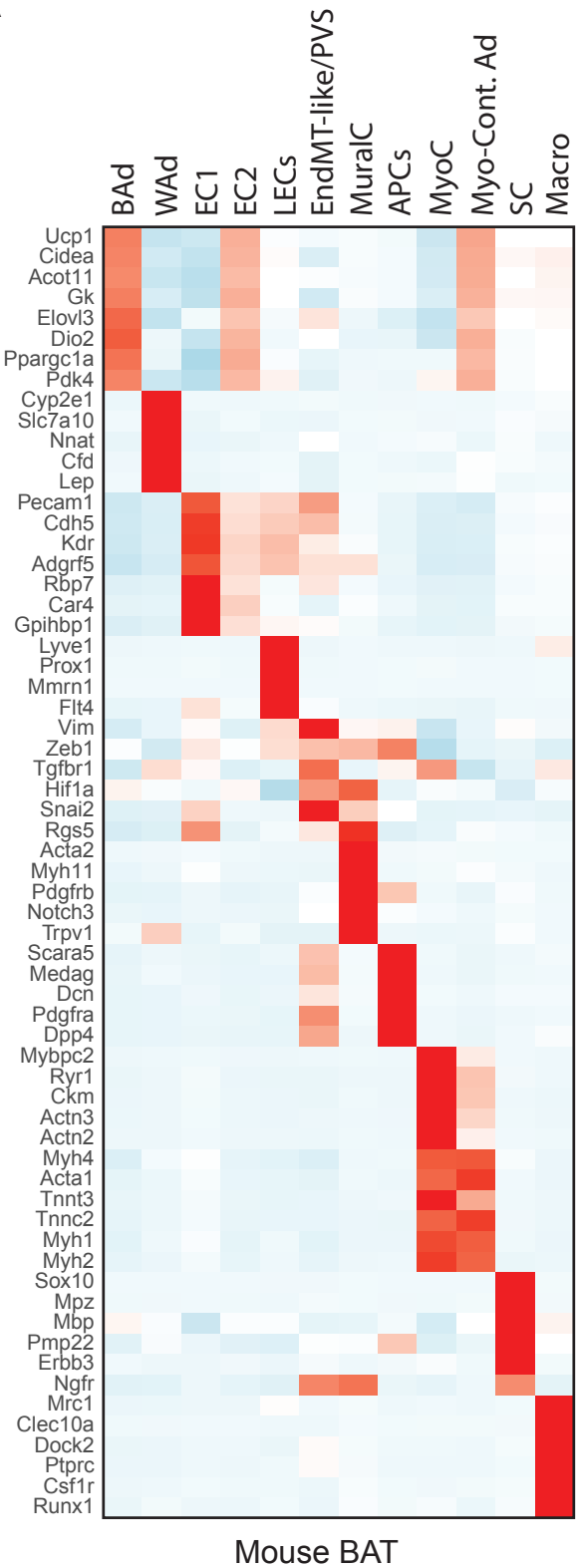

B

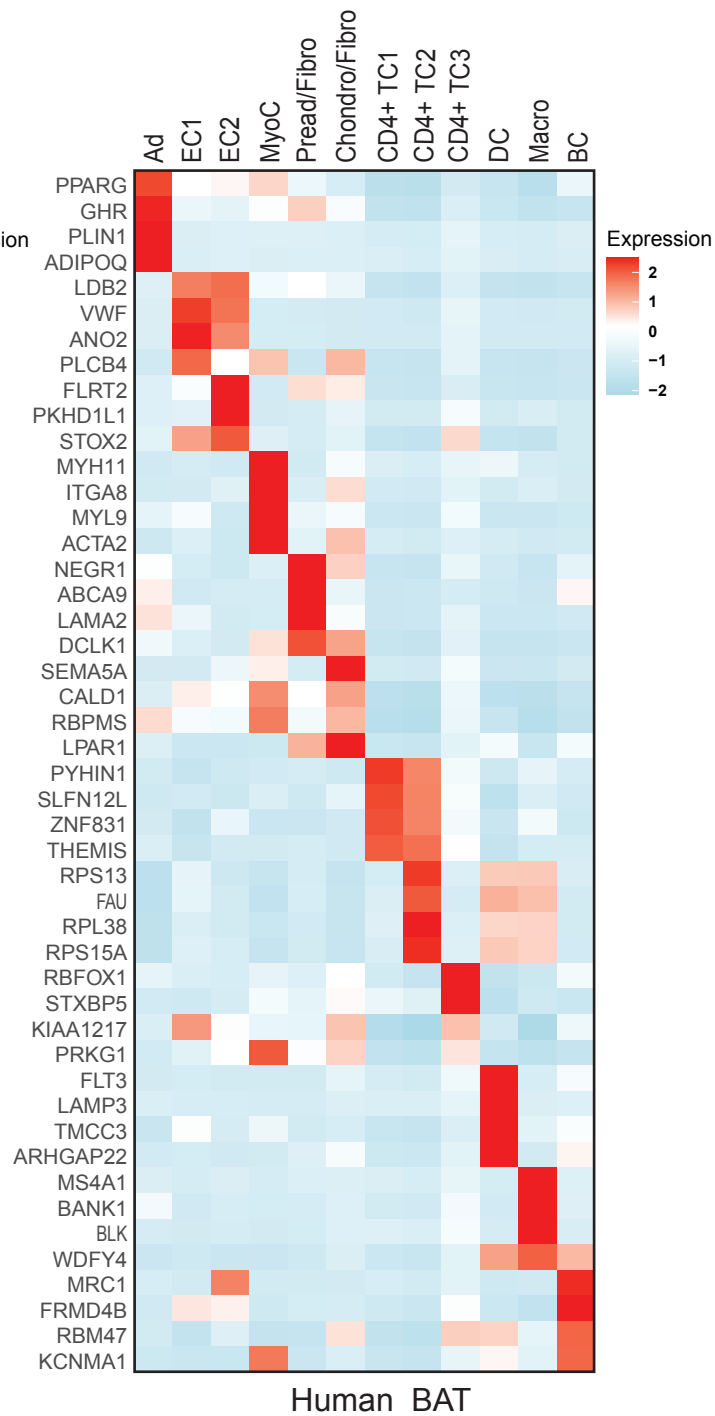

Supplement: Multimedia component 2 — Supplementary Figure 1, related toFigure 1: Heatmaps showing expression levels of canonical marker genes across cell clusters in (A) mouse and (B) human BAT. Mouse BAT data were generated in this study. Human BAT data were obtained from a previously published study by Sun W. et al. [38]. [file mmc2.pdf]

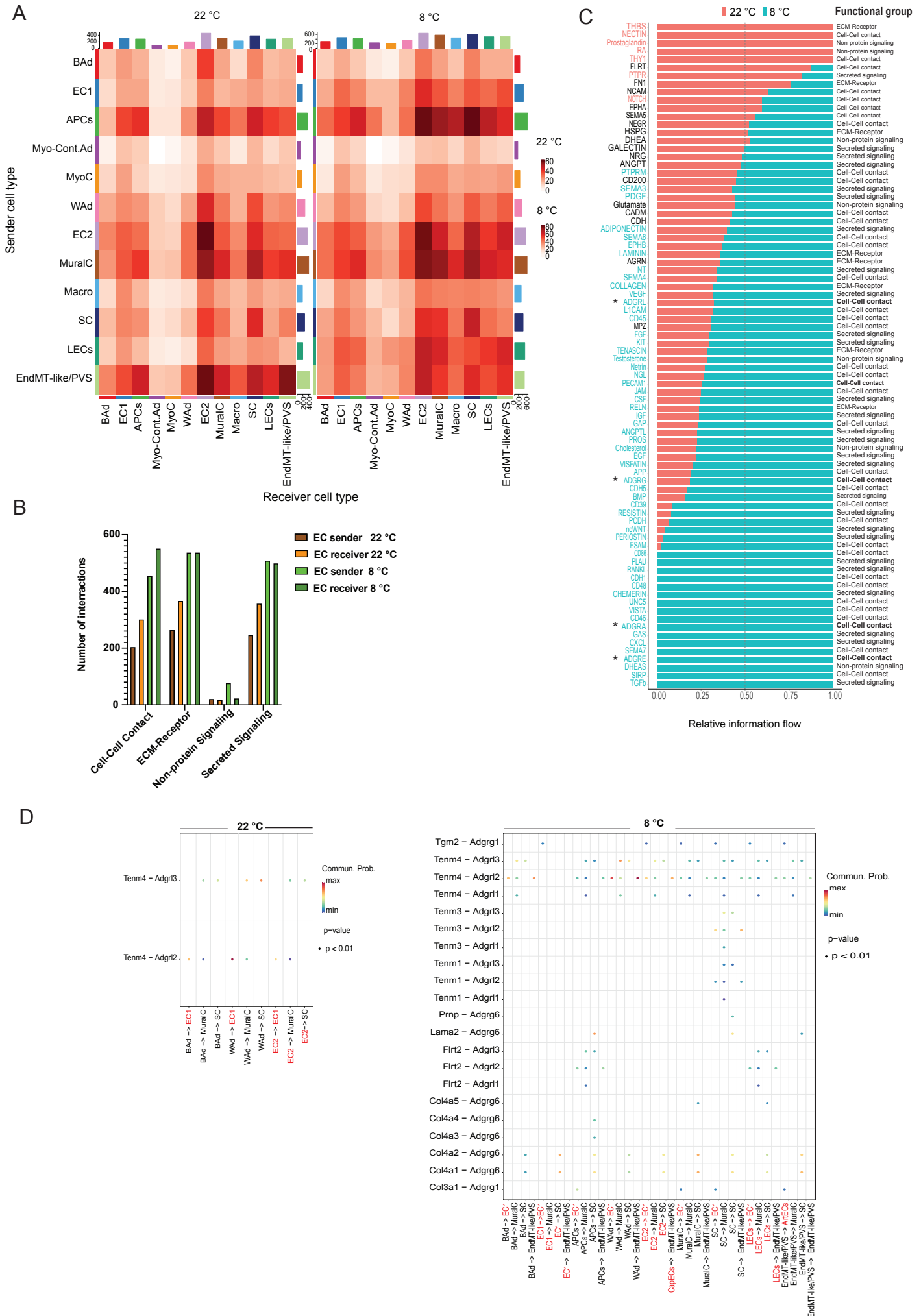

Supplement: Multimedia component 3 — Supplementrary Figure 2, related toFigure 2: Temperature-related interactions between cell types in BAT. (A) Heatmaps showing the interaction score between indicating cell types, as senders or receivers. (B) Number of interactions of different categories (cell–cell contact, ECM-receptor, non-protein signaling, and secreted signaling involving endothelial cell (EC) as sender or receiver at indicated ambient temperatures. (C) Bar plots showing the relative information flow for significant communication pathways between all BAT cell types.∗ indicates the aGPCRs pathways. (D) Dot plots presenting significant ligand-aGPCRs receptors predicted to participate in the communication of indicated cell types. The colours of the circles correspond to communication probability. ECs are highlighted in red colour. [file mmc3.pdf]

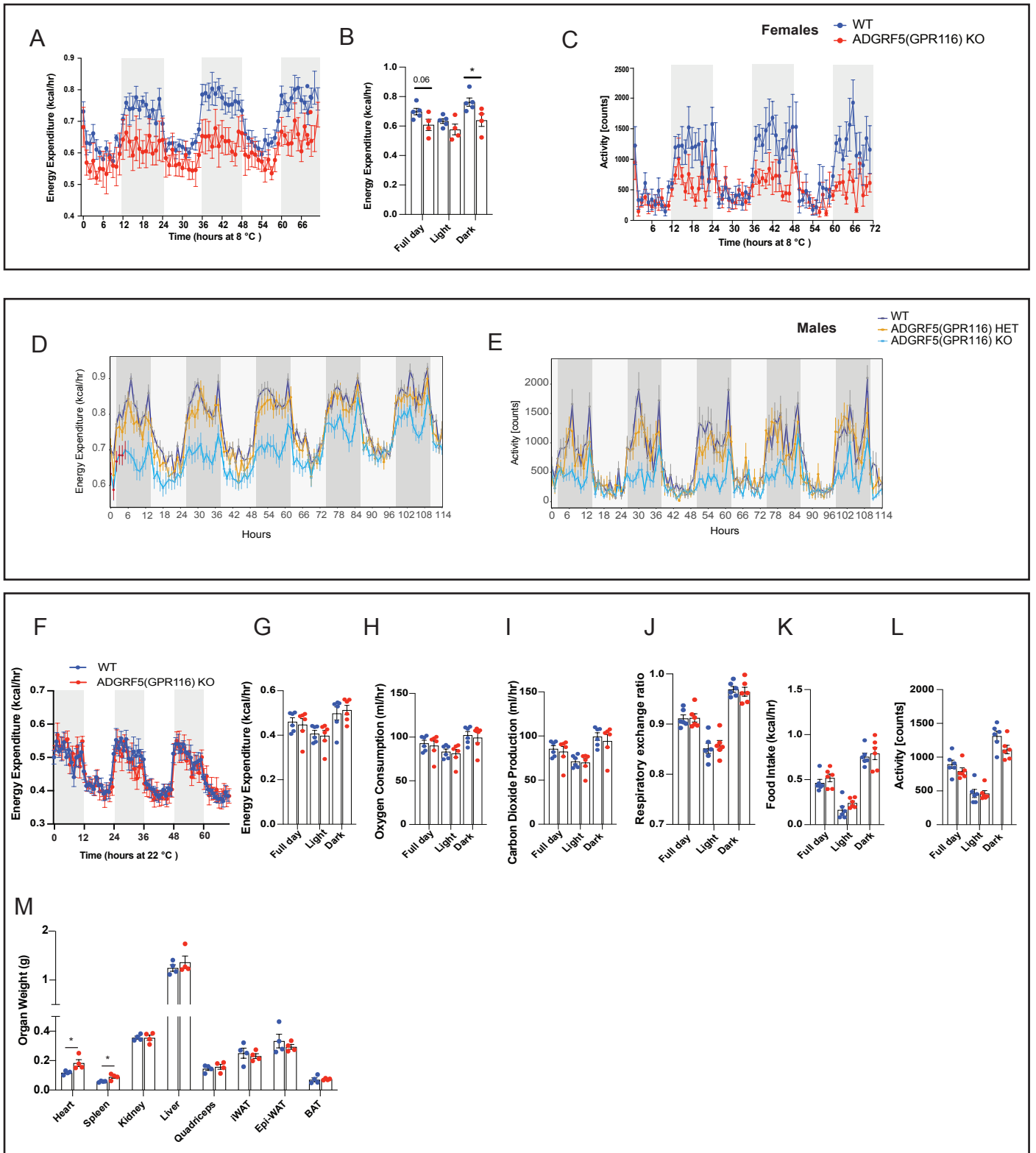

Supplement: Multimedia component 4 — Supplementary Figure 3, related toFigure 3: Metabolic characterization of ADGRF5(GPR116) KO mice at room temperature (22 °C), including cold challenged female and heterozygous cohorts. (A-C) Female mice. Time-resolved energy expenditure (A) and mean energy expenditure (B) measured by indirect calorimetry in female WT and ADGRF5(GPR116) KO mice housed at 8 °C for 14 days. The last 3 days are shown. (D–E) Male mice. Time-resolved energy expenditure (D) and locomotor activity (E) in WT, ADGRF5(GPR116) heterozygous (HET), and ADGRF5(GPR116) KO male mice housed at 8 °C for 14 days. The last 5 days are shown. (F–L) Male mice at room temperature (22 °C). Time-resolved energy expenditure (F) and average metabolic parameters including energy expenditure (G), oxygen consumption (H), carbon dioxide production (I), respiratory exchange ratio (J), food intake (K), and locomotor activity (L), analyzed across full day, light, and dark phases. Grey shaded areas indicate the dark phase. (M) Organ weights from male WT and ADGRF5(GPR116) KO mice at the end of the experimental protocol. Data are shown as mean ± SEM. Each dot represents one mouse. Statistical significance is indicated as ∗ p < 0.05, ∗∗p < 0.01; otherwise, differences are not significant (ns). [file mmc4.pdf]

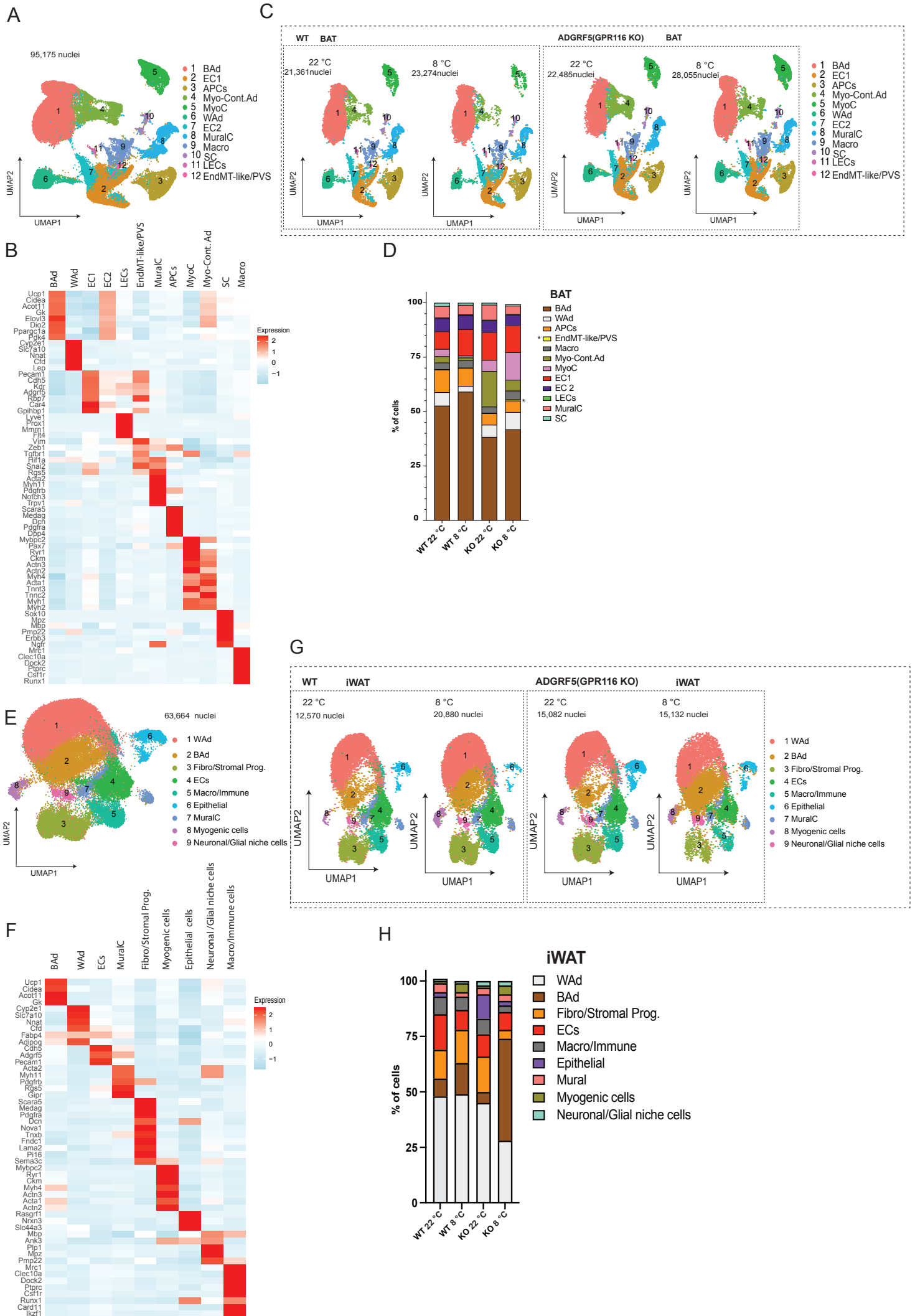

Supplement: Multimedia component 5 — Supplementary Figure 4, related toFigure 4, Figure 6: Single-nucleus transcriptomic atlas of brown and white adipose tissue across genotypes and temperatures. (A) UMAP representation of integrated single-nucleus RNA-seq data from brown adipose tissue (BAT), showing major adipocyte, endothelial, stromal, immune, and mural cell populations. Cell types were annotated based on established marker gene expression. (B) Heatmap showing scaled expression of canonical marker genes used to define the major BAT cell populations shown in (A). (C) UMAP representations of BAT nuclei from WT and ADGRF5(GPR116) KO mice housed at 22 °C or 8 °C, illustrating the distribution of major cell populations across genotypes and temperature conditions. The number of nuclei analyzed per condition is indicated. (D) Stacked bar plot showing the relative proportions of major BAT cell populations across genotypes and temperatures. (E) UMAP representation of integrated single-nucleus RNA-seq data from inguinal white adipose tissue (iWAT), showing major adipocyte, endothelial, stromal, immune, epithelial, mural, and niche-associated cell populations. (F) Heatmap showing scaled expression of canonical marker genes used to annotate iWAT cell populations shown in (E). (G) UMAP representations of iWAT nuclei from WT and ADGRF5(GPR116) KO mice housed at 22 °C or 8 °C, demonstrating comparable cellular organization across genotypes and thermal conditions. The number of nuclei analyzed per condition is indicated. (H) Stacked bar plot showing the relative proportions of major iWAT cell populations across genotypes and temperatures. [file mmc5.pdf]
