# Supplementary material for: Endothelial ADGRF5(GPR116) governs vascular adaptation required for sustained thermogenic remodeling of brown adipose tissue
Source: Mol Metab. 2026 Mar 6;107:102346. doi: 10.1016/j.molmet.2026.102346 (PMC13053757; doi:10.1016/j.molmet.2026.102346)
Supplement: Multimedia component 14 [file mmc14.docx]

Supplementary Tables

**Supplementary Table 1 (Methods) Software and algorithms details**

| Seurat | Hao, Y., et al.2023 | 5.2.1 | <https://satijalab.org/seurat/> |
| --- | --- | --- | --- |
| dplyr | Wickham H, François R, Henry L, Müller K, Vaughan D,2023 | 1.1.4 | <https://dplyr.tidyverse.org/authors.html> |
| Matrix | Bates D, Maechler M, Jagan M (2024) | 1.6-5 | <https://github.com/cran/Matrix> |
| ggplot2 | H. Wickham  (2016) | 3.5.1 | <https://github.com/tidyverse/ggplot2> |
| igraph | Csardi G, Nepusz T (2006) | 2.1.4 | <https://github.com/igraph/rigraph> |
| leiden | S. Thomas Kelly (2023),  Traag, 2019 | 0.4.3.1 | <https://github.com/TomKellyGenetics/leiden> |
| MAST | McDavid A, Finak G, Yajima M (2023) | 1.28.0 | <https://github.com/RGLab/MAST> |
| CellChat | Jin S (2024) | 2.1.2 | <https://github.com/jinworks/CellChat> |
| decoupleR | Badia-i-Mompel P., Vélez Santiago J., Braunger J., Geiss C., Dimitrov D., Müller-Dott S.,  Taus P., Dugourd A., Holland C.H., Ramirez Flores R.O. and Saez-Rodriguez J. 2022. | 2.8.0 | <https://github.com/saezlab/decoupleR_manuscript> |
| tibble | Müller K, Wickham H (2023) | 3.2.1 | <https://github.com/tidyverse/tibble> |
| tidyr | Wickham H, Vaughan D, Girlich M (2024) | 1.3.1 | <https://github.com/tidyverse/tidyr> |
| patchwork | Pedersen T (2024) | 1.3.0 | <https://github.com/thomasp85/patchwork> |
| pheatmap | Kolde R (2019) | **1.0.12** | <https://github.com/raivokolde/pheatmap> |
| UpSetR | Conway, Lex et al. 2017 |  | <https://github.com/hms-dbmi/UpSetR> |
| readxl | Hadley Wickham and Jennifer Bryan,2023 | version 1.4.3 | <https://CRAN.R-project.org/package=readxl> |
| tidyverse | Wickham H et al,2019 | version 2.0.0 | <https://doi.org/10.21105/joss.01686> |
| clusterProfiler | (29) | **version 4.10.01** | https://github.com/YuLab-SMU/clusterProfiler |
| org.Mm.eg.db | Carlson M,2023 | Version 3.18.0 | <https://github.com/ruby-on-bioc/org.Mm.eg.db> |
| DOSE | Yu, 2015 | Version 3.28.2 | <https://github.com/YuLab-SMU/DOSE> |
| Complexheatmap | Gu, Z. (2016)  Gu, Z. (2022) | 2.18.0 | <https://github.com/jokergoo/ComplexHeatmap> |
| scCustomize | Marsh S (2024) | 3.0.1 | <https://github.com/samuel-marsh/scCustomize> |
| R Studio | R core Team,2023 | version 4.3.1 | <https://www.R-project.org/> |
| YouTubeTutorials  (Single Cell analysis tutorials) | Patel K. (2021) |  | <https://github.com/kpatel427/YouTubeTutorials> |
| NicheNet | Nichenetr Browaeys, R., et al. (2020) | Version 2.2.1 | <https://github.com/saeyslab/nichenetr>  [ttps://github.com/saeyslab/nichenetr](https://github.com/saeyslab/nichenetr) |

**Supplementary Table 2: Number of nuclei and cell frequencies for BAT clusters per genotypes and ambient temperature**

| **Cluster name** | **Number of nuclei** | | | | **% of nuclei** | | | |
| --- | --- | --- | --- | --- | --- | --- | --- | --- |
|  | **BAT WT RT** | **BAT WT CE** | **BAT KO RT** | **BAT KO CE** | **BAT**  **WT RT** | **BAT WT CE** | **BAT**  **KO RT** | **BAT KO CE** |
| Brown adipocytes (BAd) | 11254 | 13758 | 8620 | 11735 | 52.7 | 59.1 | 38.3 | 41.8 |
| White adipocytes (WAd) | 1333 | 609 | 1267 | 2235 | 6.2 | 2.6 | 5.6 | 8.0 |
| Endothelial cells 1 (EC1) | 1733 | 2833 | 2891 | 3471 | 8.1 | 12.2 | 12.9 | 12.4 |
| Endothelial cells 2 (EC2) | 1278 | 1505 | 1197 | 1373 | 6.0 | 6.5 | 5.3 | 4.9 |
| Endothelial-to mesenchymal-like /perivascular stromal (mixed) (EndMT/PVS) | 71 | 69 | 88 | 86 | 0.3 | 0.3 | 0.4 | 0.3 |
| Lymphatic endothelial cells (LECs) | 11 | 30 | 34 | 167 | 0.1 | 0.1 | 0.2 | 0.6 |
| Mural cells (MuralC) | 1122 | 1018 | 1546 | 1069 | 5.3 | 4.4 | 6.9 | 3.8 |
| Adipogenic progenitor cells (APCs) | 2219 | 1935 | 1181 | 1664 | 10.4 | 8.3 | 5.3 | 5.9 |
| Myocytes (MyoC) | 716 | 211 | 1132 | 3534 | 3.4 | 0.9 | 5.0 | 12.6 |
| Myogenic-contractile adipocytes (Myo-Cont.Ad) | 615 | 304 | 3671 | 1407 | 2.9 | 1.3 | 16.3 | 5.0 |
| Schwann cells (SC) | 327 | 223 | 201 | 193 | 1.5 | 1.0 | 0.9 | 0.7 |
| Macrophages (Macro) | 682 | 779 | 657 | 1121 | 3.2 | 3.3 | 2.9 | 4.0 |

**Supplementary Table 3: Number of nuclei and cell frequencies for iWAT clusters per genotypes and ambient temperature**

| **Cluster name** | **Number of nuclei** | | | | **% of nuclei** | | | |
| --- | --- | --- | --- | --- | --- | --- | --- | --- |
|  | **iWAT WT RT** | **iWAT WT CE** | **iWAT KO RT** | **iWAT KO CE** | **iWAT WT RT** | **iWAT WT CE** | **iWAT KO RT** | **iWAT KO CE** |
| Beige adipocytes (BAd) | 986 | 2936 | 772 | 6954 | 8 | 14 | 5 | 46 |
| White-like/lipogenic adipocytes (WAd) | 5990 | 10156 | 6762 | 4238 | 48 | 49 | 45 | 28 |
| Endothelial cells (ECs) | 2008 | 1860 | 1450 | 1160 | 16 | 9 | 10 | 8 |
| Mural cells (MuralC) | 523 | 433 | 415 | 484 | 4 | 2 | 3 | 3 |
| Fibroblasts-Stromal progenitor cells (Fibro/Stromal Prog) | 1617 | 3225 | 2488 | 569 | 13 | 15 | 16 | 4 |
| Myocytes (MyoC) | 87 | 735 | 165 | 541 | 1 | 4 | 1 | 4 |
| Epithelial cells | 291 | 99 | 1649 | 365 | 2 | 0 | 11 | 2 |
| Neuronal/ Glial niche cells | 111 | 128 | 294 | 369 | 1 | 1 | 2 | 2 |
| Macrophages-Immune cells (Macro/Immune) | 957 | 1308 | 1087 | 452 | 8 | 6 | 7 | 3 |

**Supplementary Table 4: Module descriptions used for scoring adipocyte subclusters**

**BAT**

| Adipocyte identity | Pparg ,Cebpa,Plin1, Fabp4, Adipoq, Lpl, Cd36, Dgat1, Mgll, Lipe, Slc2a4 |
| --- | --- |
| Ucp1 | Ucp1 |
| Adrb3 | Adrb3 |
| Acute cold response (adrenergic) | Nr4a1, Btc, Sik1, Heyl, Irf4, Irs2, Ppargc1a, Ppara, Decr2, Steap4, Adcy9, Pnpla7. Impdh1. Rnf182, Sh2d4a, Tmem140 |
| Futile cycling | Acta1, Acta2, Tagln, Myh4, Myh7, Mylpf, Tnnc2,  Gpd1, Gpd2. Fasn, Acaca, Ryr2, Atp2a2, Elovl3, Hadhb. Ndufa4l2 |
| Oxidative/mitophagy program | Depp1, Bnip3, Pla2g7, Gmpr, Ugp2, Zbtb16, Sgpl1, Adam12, Ppargc1a, Adcy3, Egln1, Ehhadh, Ephx2, Ncoa7, Mapkapk3 |
| Neurovascular remodelling | Bmp8b, Elovl3, Crybg2, Slc2a5, Sorcs2, Prune2, Hephl1, Sorl1, Als2cl, Mlph, Adcy10, Ryr2, Fgf9, Tmem132b |
| Metabolic stress and detoxification | Cyp2b10, Gfra1, Syn2, Slc7a11, Notch4, Elavl3, Hcn2, Mfge8, Sqle, Ldlr, Npr3, Col12a1, Akr1c14, Gsta3 |
| Slow myogenic program | Myh7, Tnnt1, Myl2, Tnni1, Hspb7, Cox6a2, Flnc, Xirp1, Lmod3, Cacna1s, Jph2, Scn4a, Myom1 |
| Fast contractile myogenic program | Myh4, Mybpc2, Tnni2, Mylpf, Tnnt3, Tnnc2, Ckm, Atp2a1, Myh1, Myh2, Smtnl1, Myom2, Eef1a2, Pvalb, Tnnt3, Pvalb, Actn3 |
| Housekeeping brown adipocyte process | Nat8l, Tmem79, Cirbp, Pet100, Tmem132b, Lctl, Rcc2, Pabpn1, Mettl24, Vat1l, Tmem259, Pex6, Acbd4, Dffb, Coq10b |
| White-like lipogenic program | Aldh1a1, Retn, Nnat, Cyp2f2, Ffar2, Gsta3, Apcdd1, Gata6, Fads1, Slc25a10, Gda, Lhfpl2, C4b, Ucp2, Scd1 |
| Vascular immune interaction | Igfbp7, Adamtsl3, Svep1, Igf1, Cish, Il15, Pik3r1,  Resf1, Acvr1, Acvr1c, Ext1, Slc1a3, Flvcr1, Nampt, Csf1, Lgals3bp, Parp9, Parp12, Il6ra |
| Endothelial identity | Pik3r6, Adgrl4, Flt1, Eng, Erg, Meox2, Adgrf5, Fgd5, Cxcl12, Arhgef15, Bmp6, Rftn1, Cd200, Ushbp1 |
| Interferon signaling | Iigp1, Oasl1, Gbp10, Gbp2, Gbp5, Rsad2, Mx2, Slfn8, Ddx60, Igtp, Irgm2, Ifit1, Ifit3, Xaf1, Parp9, Parp12, Lgals9, Csf1 |

**iWAT**

| Adipocyte identity | Pparg, Cebpa, Plin1, Fabp4, Adipoq, Lpl, Cd36, Dgat1, Mgll, Lipe, Slc2a4 |
| --- | --- |
| Ucp1 | Ucp1 |
| Adrb3 | Adrb3 |
| Futile cycling | Acta1, Acta2, Myh4, Myh7, Mylpf, Tnnc2,  Gpd1, Gpd2, Fasn, Acaca, Ryr2, Atp2a2, Elovl3, Hadhb |
| White to Beige priming | Slc7a10, Nnat, Slc1a3, Cidec, Tmem120a |
| Nutrient stress signaling | Fnip2, Cdk8, Ift74, Fasn, Rgs2 |
| White adipocyte identity | Npr3, Cyp2e1, Fasn, Lpl, Slc27a1 |
| Thermogenic beige program | Ucp1, Ppargc1a, Ppargc1b, Cox7a1, Cox8b, Elovl3, Dio2, Cidea |
| Beta oxidation | Cpt1b, Acot11, Pdk4, Acadl, Acadvl, Slc25a20 |
| Myogenic Cytoskeletal Stress | Myh4, Dmd, Neb, Ttn, Actn2, Ckm |
| ECM Stromal Interaction | Fndc1, Tnxb, Flrt2, Col6a3 |
| Proliferative Immature adipocytes | Ccnb1ip1, Rpph1, Scarna2. Camk1d |

**Supplementary Table 5: Number of nuclei and cell frequencies for BAT adipocytes subclusters**

| **Cluster name** | **Number of nuclei** | | | | **% of nuclei** | | | |
| --- | --- | --- | --- | --- | --- | --- | --- | --- |
|  | **BAT WT RT** | **BAT WT CE** | **BAT KO RT** | **BAT KO CE** | **BAT**  **WT RT** | **BAT WT CE** | **BAT**  **KO RT** | **BAT KO CE** |
| oxidative/mitophagy-enriched brown adipocytes | 663 | 6214 | 286 | 387 | 5.0 | 42.4 | 2.1 | 2.5 |
| Early cold-response adipocytes | 3816 | 5907 | 2080 | 610 | 28.9 | 40.3 | 15.3 | 4.0 |
| UCP1-high, adrenergic- Adrb3 low neuro-vascular remodeling adipocytes | 1321 | 662 | 113 | 5495 | 10.0 | 4.5 | 0.8 | 35.7 |
| Metabolic-stress/detoxifying adipocytes | 1 | 124 | 0 | 5299 | 0.0 | 0.8 | 0.0 | 34.5 |
| Basal/housekeeping brown adipocytes | 3869 | 810 | 2617 | 153 | 29.3 | 5.5 | 19.3 | 1.0 |
| Vascular–immune interacting adipocytes | 1344 | 98 | 3578 | 82 | 10.2 | 0.7 | 26.4 | 0.5 |
| Myogenic-constractile adipocytes I (myofibrillar dominant) | 157 | 43 | 1868 | 399 | 1.2 | 0.3 | 13.8 | 2.6 |
| Myogenic-contractile adipocytes II (adipocyte-comitted) | 144 | 69 | 939 | 586 | 1.1 | 0.5 | 6.9 | 3.8 |
| Interferon-responsive adipocytes | 30 | 27 | 70 | 17 | 0.2 | 0.2 | 0.5 | 0.1 |
| White-like lipogenic adipocytes | 1512 | 570 | 1183 | 2050 | 11.5 | 3.9 | 8.7 | 13.3 |
| Endothelial identity adipocytes | 345 | 147 | 824 | 299 | 2.6 | 1.0 | 6.1 | 1.9 |

**Supplementary Table 6: Number of nuclei and cell frequencies for iWAT adipocytes subclusters**

| **Cluster name** | **Number of nuclei** | | | | **% of nuclei** | | | |
| --- | --- | --- | --- | --- | --- | --- | --- | --- |
|  | **BAT WT RT** | **BAT WT CE** | **BAT KO RT** | **BAT KO CE** | **BAT**  **WT RT** | **BAT WT CE** | **BAT**  **KO RT** | **BAT KO CE** |
| Classical oxidative beige adipocytes | 827 | 2665 | 766 | 5169 | 11.9 | 20.4 | 10.2 | 46.2 |
| Futile-cycle, myogenic pseudo-beige adipocytes | 178 | 313 | 166 | 2158 | 2.6 | 2.4 | 2.2 | 19.3 |
| White-to-beige primed adipocytes | 2838 | 3672 | 2884 | 2543 | 40.7 | 28.0 | 38.3 | 22.7 |
| ECM-interacting transitional population | 708 | 1052 | 829 | 392 | 10.1 | 8.0 | 11.0 | 3.5 |
| White lipid-storage adipocytes | 1828 | 4716 | 2362 | 752 | 26.2 | 36.0 | 31.4 | 6.7 |
| Stress-responsive adipocytes | 370 | 96 | 108 | 144 | 5.3 | 0.7 | 1.4 | 1.3 |
| Cycling and preadipocyte-like adipocytes | 227 | 578 | 419 | 34 | 3.3 | 4.4 | 5.6 | 0.3 |

**Supplementary Table 7: Number of nuclei and cell frequencies for BAT vascular subclusters**

| **Cluster name** | **Number of nuclei** | | | | **% of nuclei** | | | | |
| --- | --- | --- | --- | --- | --- | --- | --- | --- | --- |
|  | BAT WT RT | BAT WT CE | BAT KO RT | BAT KO CE | BAT  WT RT | BAT WT CE | BAT  KO RT | BAT KO CE |  |
| Aerocyte-like capillary ECs | 749 | 1289 | 737 | 1271 | 17.8 | 23.6 | 12.8 | 20.6 |  |
| Canonical arterial ECs | 189 | 462 | 250 | 528 | 4.5 | 8.5 | 4.3 | 8.6 |  |
| Arterial remodeling state | 132 | 235 | 173 | 177 | 3.1 | 4.3 | 3.0 | 2.9 |  |
| Arterial-like EC cluster | 142 | 209 | 153 | 129 | 3.4 | 3.8 | 2.7 | 2.1 |  |
| Venular ECs | 355 | 396 | 348 | 619 | 8.4 | 7.3 | 6.0 | 10.0 |  |
| Venular-like cluster | 73 | 79 | 66 | 111 | 1.7 | 1.4 | 1.1 | 1.8 |  |
| Metabolic capillary ECs | 0 | 8 | 0 | 316 | 0.0 | 0.1 | 0.0 | 5.1 |  |
| Lymphatic ECs | 65 | 70 | 75 | 85 | 1.5 | 1.3 | 1.3 | 1.4 |  |
| Classical pericytes | 248 | 349 | 274 | 407 | 5.9 | 6.4 | 4.8 | 6.6 |  |
| Metabolically active pericytes | 193 | 314 | 585 | 271 | 4.6 | 5.8 | 10.2 | 4.4 |  |
| Vascular smooth muscle cells (cluster 11) | 95 | 93 | 199 | 78 | 2.3 | 1.7 | 3.5 | 1.3 |  |
| Vascular smooth muscle cells (cluster 12) | 339 | 316 | 593 | 286 | 8.0 | 5.8 | 10.3 | 4.6 |  |
| Adventitial fibro-immune stromal | 403 | 286 | 396 | 275 | 9.6 | 5.2 | 6.9 | 4.5 |  |
| Proliferative EndMT-like state | 12 | 33 | 31 | 212 | 0.3 | 0.6 | 0.5 | 3.4 |  |
| Myofibroblast-like mesenchymal cells | 35 | 35 | 34 | 24 | 0.8 | 0.6 | 0.6 | 0.4 |  |
| Adipocyte-like EC signature (Cluster 16) | 572 | 593 | 732 | 318 | 13.6 | 10.9 | 12.7 | 5.2 |  |
| Adipocyte-like EC signature (Cluster 17) | 554 | 643 | 490 | 519 | 13.1 | 11.8 | 8.5 | 8.4 |  |
| Myofiber-like EC signature (Cluster 18) | 25 | 17 | 240 | 182 | 0.6 | 0.3 | 4.2 | 3.0 |  |
| Myofiber-like EC signature (Cluster 19) | 34 | 28 | 380 | 358 | 0.8 | 0.5 | 6.6 | 5.8 |  |

**Supplementary Table 8: Number of nuclei and cell frequencies for iWAT vascular subclusters**

| **Cluster name** | **Number of nuclei** | | | | **% of nuclei** | | | |
| --- | --- | --- | --- | --- | --- | --- | --- | --- |
|  | **BAT WT RT** | **BAT WT CE** | **BAT KO RT** | **BAT KO CE** | **BAT**  **WT RT** | **BAT WT CE** | **BAT**  **KO RT** | **BAT KO CE** |
| Metabolically-enriched capillary ECs | 499 | 381 | 383 | 468 | 19.7 | 16.6 | 20.5 | 28.5 |
| Lipid handling capillary ECs | 502 | 477 | 306 | 387 | 19.8 | 20.8 | 16.4 | 23.5 |
| Venous and post-capillary venule ECs | 183 | 347 | 210 | 93 | 7.2 | 15.1 | 11.3 | 5.7 |
| Arterial/arteriolar ECs | 95 | 164 | 119 | 66 | 3.8 | 7.2 | 6.4 | 4.0 |
| Low-complexity cluster | 441 | 249 | 233 | 209 | 17.4 | 10.9 | 12.5 | 12.7 |
| Pericytes | 356 | 190 | 198 | 90 | 14.1 | 8.3 | 10.6 | 5.5 |
| Vascular smooth muscle cell | 122 | 191 | 164 | 115 | 4.8 | 8.3 | 8.8 | 7.0 |
| Perivascular adventitial stromal population | 333 | 294 | 252 | 216 | 13.2 | 12.8 | 13.5 | 13.1 |

**Supplementary Table 9: Module descriptions used for scoring endothelial subclusters, combined across experimental conditions**

**BAT**

| Flow shear response | Klf2, Klf4, Dkk2, Fos, Junl, Erg, Thbd, Kdr, Nos3 |
| --- | --- |
| Tight junctions | Cldn5, Ocln, Tjp1, Tjp2, Jam2, Jam3, Amotl2 |
| Junction cytoskeleton anchoring | Actn1, Vcl, Add1, Add3, Flna, Ctnna1 |
| EndMT regulator | Snai1, Snai2, Zeb1, Serpine1, Tgfbr1 |
| ECM remodeling fibro-inflammatory niche | Col5a3, Col9a3, Col17a1, Col9a1, Cxcl1, Adamts2, Mmp2, Col26a1, Thbs1, Sparc, Lox, Col22a1, Ccl2, Mmp27, Mmp24, Mmp12 |
| EC paracrine remodeling | Rspo4, Angpt2, Serpine2, Cxcl12, Csf1, Inhba |
| EC cytoskeleton Rho signaling | Ripor2, Dock7, Arhgap35, Ppp3ca, Rock1, Arhgap1, Arhgap29, Dlc1, Ripor2, Arhgap5, Arhgap17 |

**iWAT**

| Flow shear response | Klf2, Dkk2, Erg, Kdr, Nos3 |
| --- | --- |
| Tight junction | Cldn5, Tjp1, Tjp2, Jam2, Amotl2 |
| Junction cytoskeleton anchoring | Actn1, Vcl, Add1, Add3, Flna, Ctnna1 |
| ECM remodeling fibro-inflammatory niche | Col4a3, Col4a4, Col4a5, Col4a6, Col11a1, Col12a1, Col19a1, Col22a1, Col24a1, Col25a1, Col27a1, Col28a1, Postn, Fn1 |
| EC paracrine remodeling | Nrg4, Vegfa, Bmp6, Fgf1, Lpl, Sema3a, Sema3g, Nt4, Tgfb3 |
| EC cytoskeleton Rho signaling | Itga5, Itgb1, Pxn, Tln1, Vcl, Cav1, Ripor2, Dock7, Arhgap35, Ppp3ca, Rock1, Arhgap1, Arhgap29, Dlc1, Ripor2, Arhgap5, Arhgap17 |
